# Individual hematotoxicity prediction of further chemotherapy cycles by dynamic mathematical models in patients with gastrointestinal tumors

**DOI:** 10.1007/s00432-023-04601-9

**Published:** 2023-02-28

**Authors:** Vivien Topf, Yuri Kheifetz, Severin Daum, Alexej Ballhausen, Andreas Schwarzer, Kien Vu Trung, Gertraud Stocker, Achim Aigner, Florian Lordick, Markus Scholz, Maren Knödler

**Affiliations:** 1 University Cancer Center Leipzig (UCCL), University of Leipzig Medical Center, Leipzig, Germany; 2 Department of Medicine (Oncology, Gastroenterology, Hepatology, Pulmonology and Infectiology), Medical Center, University of Leipzig, Leipzig, Germany; 3 Institute for Medical Informatics, Statistics and Epidemiology (IMISE), University of Leipzig, Leipzig, Germany; 4 Medical Department, Division of Gastroenterology, Infectious Diseases and Rheumatology, Charité University Medicine Berlin, Campus Benjamin Franklin (CBF), Berlin, Germany; 5 Medical Department, Division of Hematology, Oncology and Tumor Immunology, Charité University Medicine Berlin, Campus Virchow Hospital (CVK), Berlin, Germany; 6 Onkopraxis Probstheida, Leipzig, Germany; 7 Rudolf-Boehm-Institute for Pharmacology and Toxicology, Clinical Pharmacology, University of Leipzig, Leipzig, Germany; 8 Charité Comprehensive Cancer Center (CCCC), Charité University Medicine Berlin, Campus Charité Mitte (CCM), Virchowweg 23, 10117 Berlin, Germany

**Keywords:** Biomathematical model, Gastrointestinal cancer, Cytotoxic chemotherapy, Hematotoxicity, Pharmacodynamics, Precision medicine

## Abstract

**Purpose:**

Hematotoxicity is a common side-effect of cytotoxic gastrointestinal (GI) cancer therapies. An unsolved problem is to predict the individual risk therefore to decide on treatment adaptions. We applied an established biomathematical prediction model and primarily evaluated its predictive value in patients undergoing chemotherapy for GI cancers in curative intent.

**Methods:**

In a prospective, observational multicenter study on patients with gastro-esophageal or pancreatic cancer (*n* = 28) receiving myelosuppressive adjuvant or neoadjuvant chemotherapy (FLO(T) or FOLFIRINOX), individual model parameters were learned based on patients’ observed laboratory values during the first chemotherapy cycle and further external data resources. Grades of hematotoxicity of subsequent cycles were predicted by model simulation and compared with observed data.

**Results:**

The most common high-grade hematological toxicity was neutropenia [19/28 patients (68%)]. For the FLO(T) regimen, individual grades of thrombocytopenia and leukopenia could be well predicted for cycles 2–4, as well as grades of neutropenia for cycle 2. Prediction accuracy for neutropenia in the third and fourth cycle differed by one toxicity grade on average. For the FOLFIRINOX-regimen, thrombocytopenia predictions showed a maximum deviation of one toxicity grade up to the end of therapy (8 cycles). Deviations of predictions were less than one degree on average up to cycle 4 for neutropenia, and up to cycle 6 for leukopenia.

**Conclusion:**

The biomathematical model showed excellent short-term and decent long-term prediction performance for all relevant hematological side effects associated with FLO(T)/FOLFIRINOX. Clinical utility of this precision-medicine approach needs to be further investigated in a larger cohort.

**Supplementary Information:**

The online version contains supplementary material available at 10.1007/s00432-023-04601-9.

## Introduction

Neoadjuvant and adjuvant chemotherapy in combination with radical surgery is a standard approach to improve the prognosis of gastrointestinal (GI) cancer. The FLOT regimen (5-fluorouracil, leucovorin, oxaliplatin, and docetaxel) was established as perioperative concept for gastric and gastro-esophageal cancer (Al-Batran et al. 2019; Giommoni et al. 2021). Likewise, FOLFIRINOX (5-fluorouracil, leucovorin, oxaliplatin, irinotecan) is used as adjuvant or neoadjuvant chemotherapy for pancreatic cancer (Conroy et al. 2018). Both regimens are associated with high toxicity, in particular hematotoxicity. This can lead to treatment delays which eventually may worsen treatment efficacy and patients’ survival chances. Chemotherapy-related toxicities are heterogeneous among patients and difficult to predict, based on clinical characteristics of the patient. Except for the observed correlation between a deficiency in the enzyme dihydropyrimidine dehydrogenase and an increased incidence of side effects during 5-fluorouracil therapy (Wigle et al. 2019), it is still largely unclear which factors contribute to the observed inter-individual heterogeneity. Predictive information on the individual’s risk is of high clinical relevance, since it would allow for individually optimized treatment delivery. Biomathematical models of hematopoiesis under chemotherapy were proposed to aid this task (Schirm et al. 2014; Kheifetz and Scholz 2019, 2021), but their performance was not yet tested in prospective real-world settings, and only to a small extent, for solid cancer therapies including a few adjuvant breast and lung cancer therapies (Schirm et al. 2014). We designed a prospective non-interventional trial to evaluate this modeling approach for patients with GI cancers undergoing chemotherapy in curative intent. The main question was whether the biomathematical model would be able to predict individual toxicities occurring during the course of chemotherapy pursuing toward a model-based precision-medicine approach.

## Methods

### Patients

Patients with locally advanced gastric or gastro-esophageal cancer (AEG I–III according to Siewert) as well as patients with pancreatic cancer participated in this study. Inclusion criteria comprised planned neoadjuvant or adjuvant chemotherapy with the FLO(T) or FOLFIRINOX-regimen. Furthermore, study participants had to be at least 18 years old at study entry. Signed informed consent was required. Minors, pregnant, and lactating patients as well as patients with marked anemia (hemoglobin < 4.6 mmol/l) were excluded from the study.

### Treatment

Chemotherapy was administered according to national and international recommendations (Lorenzen et al. 2013; Lordick et al. 2022; https://www.onkopedia.com; https://www.leitlinienprogramm-onkologie.de). In the FLO(T) regimen, patients received four preoperative cycles of (50 mg/m^2^ docetaxel), 85 mg/m^2^ oxaliplatin, 200 mg/m^2^ leucovorin, and 2600 mg/m^2^ fluorouracil as a 24-h infusion on day 1, repeated every 2 weeks (Al-Batran et al. 2019). In the FOLFIRINOX-regimen, 85 mg/m^2^ oxaliplatin, 180 mg/m^2^ irinotecan, 400 mg/m^2^ leucovorin, and 400 mg/m^2^ fluorouracil, followed by 2400 mg/m^2^ fluorouracil as a 46-h continuous infusion, were given every 2 weeks (Conroy et al. 2018).

### Clinical data collection

The study was initiated at the University Cancer Center Leipzig (UCCL) at University of Leipzig Medical Center (UKL), Germany. This center also acted as the main recruitment and study coordinating center. Additional German centers that supported recruitment were: Medical Department, Division of Gastroenterology, Infectious Diseases and Rheumatology, Charité University Medicine Berlin, Campus Benjamin Franklin (CBF), Medical Department, Division of Hematology, Oncology and Tumor Immunology, Charité University Medicine Berlin, Campus Virchow Hospital (CVK), and Onkopraxis Probstheida Leipzig. The recruitment period was from 01/2020 to 03/2022. A total of 28 evaluable study participants were recruited during this time period.

### Trial design

This trial was designed as a multicenter clinical prospective observational study. During the first two cycles of systemic therapy, blood was collected at close time intervals, i.e., at days 1, 3, 5, 8, 10, and 12 during cycles 1 and 2, and at days 1 and 10 from cycle 3 on. These frequent blood draws were required to assess hematopoietic dynamics as precisely as possible, in particular regarding the nadir phase of the different blood cell lines. At each blood drawing, differential blood counts were assessed. Toxicities were documented according to Common Terminology Criteria for Adverse Events (CTCAE) Version 5 for each cycle (Cancer Therapy Evaluation Program, Common Terminology Criteria for Adverse Events 2017).

### Modeling concepts

The hematopoietic computational model used here is a mechanistic biomathematical model of bone-marrow hematopoiesis, chemotherapy, and hematopoietic growth-factor effects (Kheifetz and Scholz 7/1/2022), integrating the following previously developed sub-models:

1. Model of thrombopoiesis under chemotherapy including a bone-marrow niche model of Kheifetz and Scholz (2019) and Komarova et al. (2003).
2. Model of granulopoiesis under chemotherapy (Schirm et al. 2014) including pharmacokinetics and -dynamics of G-CSF derivatives (Scholz et al. 2012).
3. Pharmacokinetic models of relevant cytotoxic drugs:
  - Linear 3-compartmental PK model of oxaliplatin (Delord et al. 2003).
  - Linear 2-compartmental PK model of 5-fluorouracil (Arshad et al. 2020).
  - Linear 3-compartmental PK model of irinotecan (Younis et al. 2009).
4. Model of non-granulopoietic leukocytes (lymphocytes, monocytes) comprising
  - Model of lymphopoiesis based on a model of Wuestermann and Cronkite (Wuestermann and Cronkite 1995).
  - Simple empirical model of monocytes

**Fig. 1:**
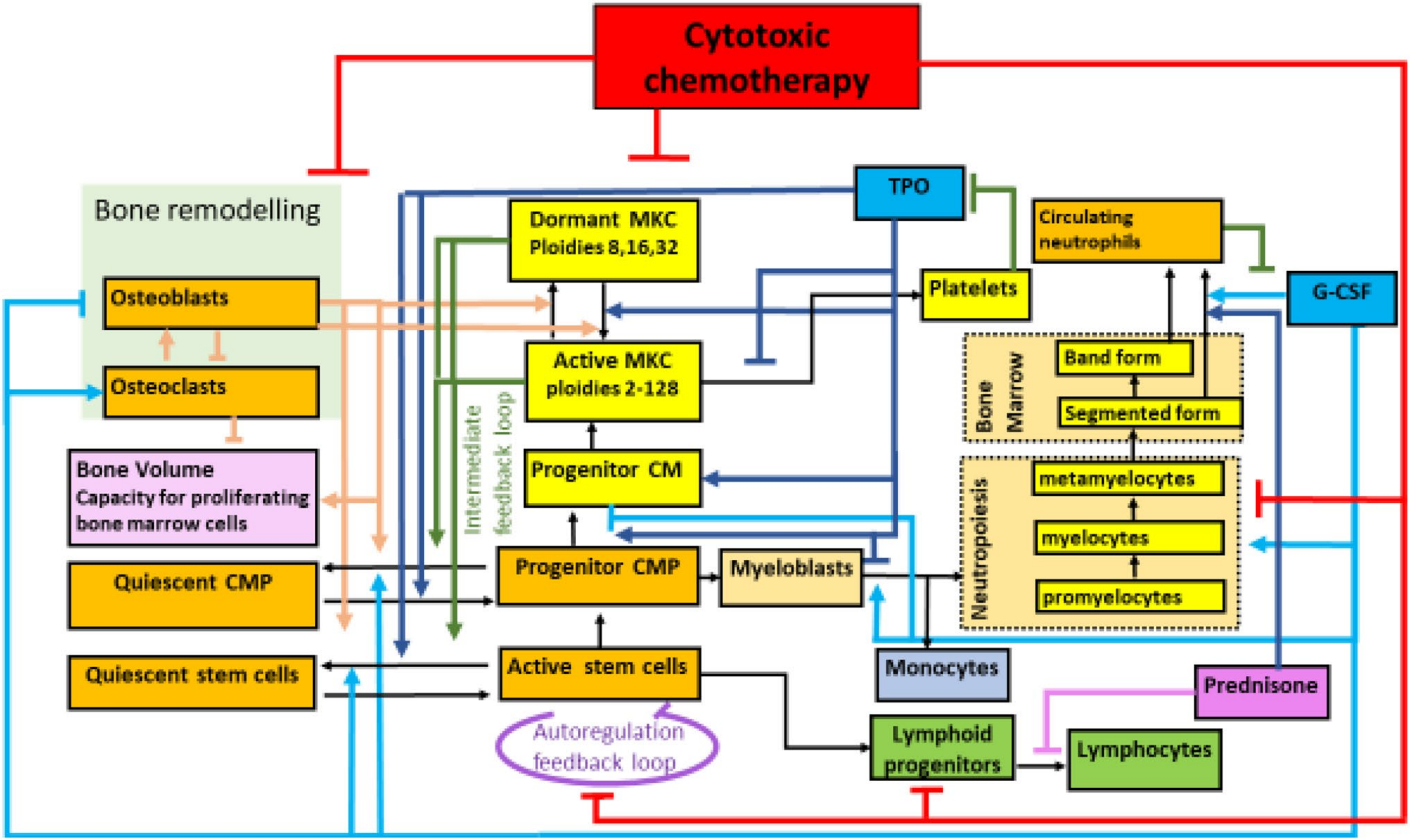
Basic structure of our integrated biomathematical model of hematopoiesis under chemotherapy as described in (Kheifetz and Scholz 7/1/2022). *CMP* active common myeloid progenitor cells, *CM* megakaryocyte precursors, *MKC* megakaryocytes. Thin black lines display transitions between different cell types. Thick colored lines represent different feedback mechanisms. Arrows imply stimulations; perpendicular lines imply suppressions

Figure 1 depicts this model schematically.

Individual and population parameters of the model were learned by the method of *virtual participation* assuming that a patient also took part on other experiments and studies penalizing deviations from respective observations (Kheifetz and Scholz 2019). This approach effectively copes with the problem of sparse data available for a single patient. In more detail, the likelihood function to be optimized contains both, terms for fitting individual patient data and for fitting other data sets (typically population averages). The penalties for deviations from the averaged biological data are determined by respective standard deviations of measurements. These penalties are considerably smaller comparing to those of deviations from real individual data. The approach not only avoids overfitting by applying information criteria, but also makes individual fits quantitatively consistent with the more and more accumulating biological knowledge of hematopoiesis such as the role of osteoblasts in explaining long-term toxic effects, megakaryocyte-mediated feedback on stem cells, bi-phasic stimulation of thrombopoiesis by TPO, dynamics of megakaryocyte ploidies, and non-exponential platelet degradation. We considered 25 different virtual experiments from ten studies, considering a total of 758 additional data points. More detailed information about the biological studies considered and the corresponding fitting results can be found in supporting information S1 and Supporting Figures S1–S23. Individual parameters were fitted using data of the first treatment cycle only. Fitted parameters of the model are listed in Table S1 of supporting information.

### Assessment of prediction performance

We assessed the predictive potential of the integrated model using a method proposed in a previous study (Kheifetz and Scholz 2021). In detail, we determined absolute deviations of predicted and observed degrees of hematotoxicity in all cycles except for the first one which was used for individual parameter estimation. We applied CTCAE degrees of toxicity (Cancer Therapy Evaluation Program, Common Terminology Criteria for Adverse Events 2017) for platelets, neutrophils, and leukocytes. The time points with lowest observed cell counts for respective cell types were considered for assessing toxicity degrees and for comparing model and data. We also analyzed the correlation between individual parameter estimates and clinical risk factors [age, body mass index (BMI), and body surface area (BSA)] by Spearman’s rank correlation coefficients.

## Results

### Characteristics of the patients

A total of 28 evaluable patients treated at four sites were recruited for this study. It was noticeable that the majority of study participants (*n* = 19, 67.9%) were overweight or obese at the time of study inclusion. The study population consisted of 14 patients (50.0%) with gastro-esophageal and 9 patients (32.1%) with gastric cancer treated with FLO(T) (*n* = 23, 82.1%) and five patients with pancreatic cancer receiving FOLFIRINOX therapy (17.9%).

These and further patient and tumor characteristics are summarized in Table 1.

**Table 1:** Baseline patient and tumor characteristics

Baseline patient characteristics	*n*	%	Baseline tumor characteristics	*n*	%
Patients	28	100	Carcinoma	28	100
Study site			Tumor entities		
University Cancer Center Leipzig	23	82.1	Gastro-esophageal cancer	14	50.0
Onkopraxis Probstheida, Leipzig	2	7.1	Gastric cancer	9	32.1
Charité Berlin, CBF	2	7.1	Pancreatic cancer	5	17.9
Charité Berlin, CVK	1	3.6	T-stage		
Sex			1	1	4.2
Male	20	71.4	2	2	8.3
Female	8	28.6	3	18	75.0
Age			4	3	12.5
Mean/minimum–maximum	63.1/43–83		Not available	4	–
< 65	15	53.6	Grading		
≥ 65	13	46.4	G1	1	5.3
35–44	1	3.6	G2	7	36.8
45–54	5	17.9	G3	10	52.6
55–64	9	32.1	Mixed	1	5.3
65–74	10	35.7	Not available	9	–
75–84	3	10.7	Laurens classification		
BMI			Intestinal	8	61.5
Mean/minimum–maximum	27.0/17.8–44.9		Diffuse	5	38.5
Underweight (<18.5)	3	10.7	Not available	10	–
Normal weight (18.5–24.9)	6	21.4	Not applicable^b^	5	–
Overweight (25.0–29.9)	12	42.9	Therapy regimes		
Obesity grade 1 (30.0–34.9)	5	17.9	FLOT	20	71.4
Obesity grade 2 (35.0–39.9)	1	3.6	FLO/mFOLFOX	3	10.7
Obesity grade 3 (≥40.0)	1	3.6	FOLFIRINOX	5	17.9
ECOG Performance Status			Number of treatment cycles		
ECOG 0	24	85.7	Gastro-esophageal cancer/gastric cancer		
ECOG 1	4	14.3			
Hurria score^a^			Neoadjuvant (preoperative)		
Mean/minimum–maximum	8.3/6–12		1	0	0.0
1–5	0	0.0	2	3	13.0
6–10	9	75.0	3	2	8.6
11–15	3	25.0	4	18	78.3
Not available	1	–	Pancreatic cancer		
Comorbidities			Neoadjuvant (preoperative)		
Charlson Comorbidity Index			1	0	0.0
0	11	39.3	2	0	0.0
1	14	50.0	3	0	0.0
2	0	0.0	4	0	0.0
3	2	7.1	5	0	0.0
4	1	3.6	6	1	3.6
Number of comorbidities			7	1	3.6
Mean/minimum–maximum	3.8/0–12		8	1	3.6
0–2	10	35.7	Adjuvant		
3–5	11	39.3	1	0	0.0
6–8	6	21.4	2	1	3.6
9–12	1	3.6	3	0	0.0
Medication			4	1	3.6
Mean/minimum–maximum	4.5/0–14				
0–5	17	63.0			
6–10	6	22.2			
11–15	4	14.8			
Not available	1	–			

^a^Only for patients with an age ≥ 65 years

^b^For patients with pancreatic cancer

### Adverse events

Hematologic toxicity was the most frequent adverse event in this patient cohort. In all study patients (75.0%), at least one hematological side-effect occurred that corresponded to at least a toxicity grade of 1 (Table 2). Accordingly, 16 out of 20 (80.0%) patients treated with FLOT experienced a high-grade (grade 3/4) hematological adverse event (AE). In contrast, none of the three patients treated with FLO/mFOLFOX, but all of the five patients treated with FOLFIRINOX experienced a grade 3/4 hematological AE. Among the high-grade toxicity events, high-grade neutropenia was the most frequent event.

**Table 2:** Hematologic adverse events of study participants according to CTCAE V5

	FLOT (*n* = 20 patients)	FLO/mFOLFOX (*n* = 3 patients)	FOLFIRINOX (*n* = 5 patients)
Grade	1/2/3/4	1/2/3/4	1/2/3/4
**Hematological**	**0/4/8/8**	**2/1/0/0**	**0/0/4/1**
Anemia	10/4/1/0	1/1/0/0	3/0/2/0
Thrombocytopenia	7/0/0/0	1/1/0/0	1/0/1/0
Leukocytopenia	0/10/5/1	2/1/0/0	0/3/1/0
Neutropenia	1/2/7/8	1/0/0/0	0/0/3/1

A total of 17 patients (85.0%) on FLOT therapy and three patients (100.0%) on FLO/mFOLFOX as well as five patients (100.0%) on FOLFIRINOX regimens developed low-grade non-hematologic adverse events. In the FLOT patient group, polyneuropathy (*n* = 11, 55.0%), diarrhea (*n* = 9, 45.0%), and fatigue (*n* = 9, 45.0%) were the most common low-grade non-hematologic toxicities. Among study participants treated with FLO/mFOLFOX, fatigue (*n* = 2, 66.7%), constipation (*n* = 2, 66.7%), and nausea/vomiting (*n* = 2, 66.7%) occurred most frequently. In the FOLFIRINOX group, all patients developed low-grade polyneuropathy (*n* = 5, 100.0%) and 60.0% (*n* = 3) suffered from nausea/vomiting. Less common non-hematologic adverse events included mucositis (*n* = 4, 20.0% of FLOT patients; *n* = 1, 20.0% of FOLFIRNOX patients). Only one patient in the FLOT group (*n* = 1, 5.0%) developed high-grade diarrhea. No high-grade non-hematologic adverse event occurred in any of the other treatment regimens.

Independent of the treatment regimen, the most common hematologic toxicity was high-grade neutropenia. A detailed overview of observed hematologic adverse events is given in Table 2.

### Parameter fitting on the basis of first-cycle data

To derive individual estimates of model parameters, we fitted the predictions of our model to respective observed blood parameters during the first therapy cycles. Only platelets, neutrophils, and leukocytes were considered for that purpose. Results are shown in Supporting Figs. S24–S51 (Supporting Figures S24-S51 RealFits.pptx). Examples of individual patients are shown in Fig. 2.

**Fig. 2:**
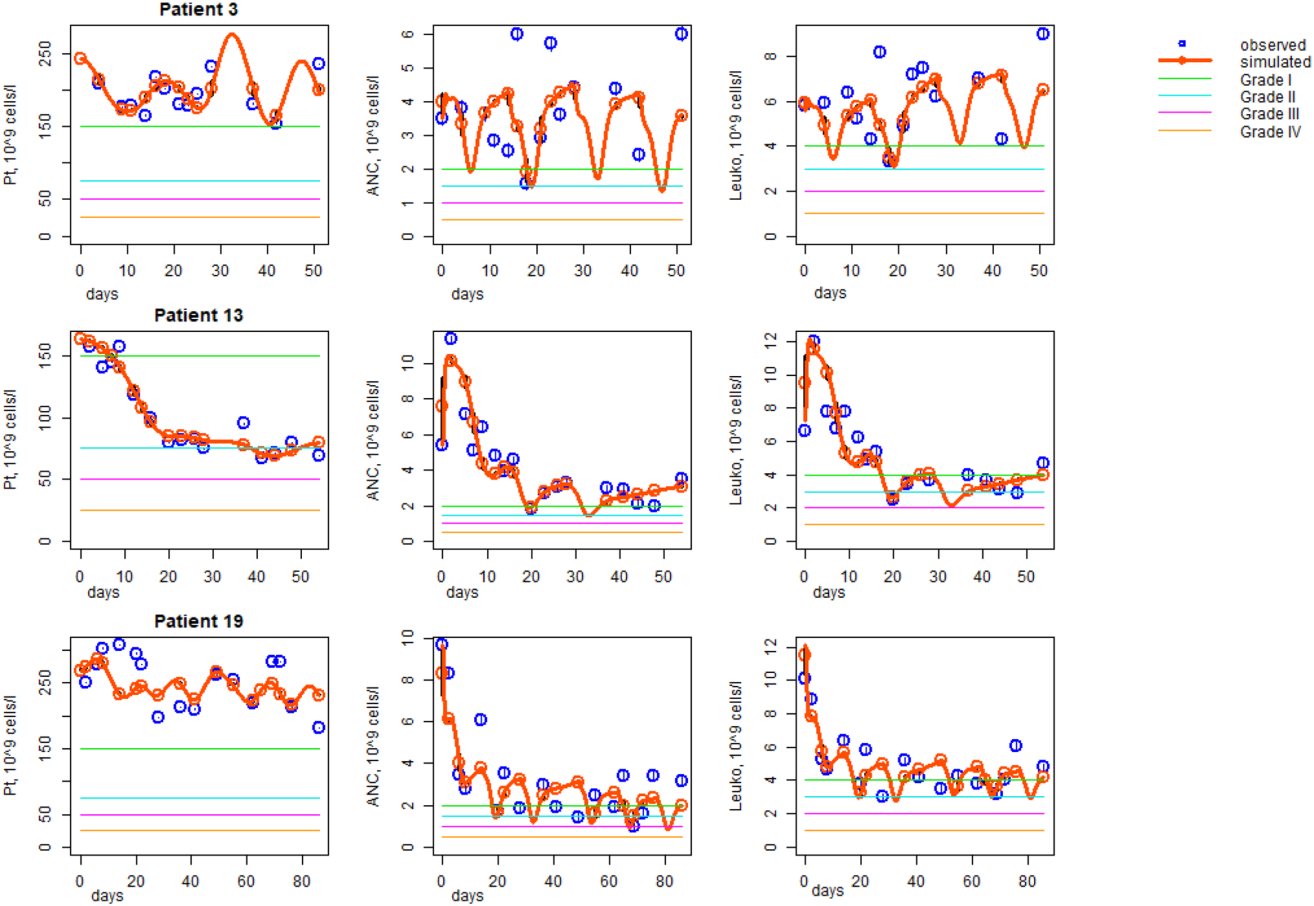
Fitting results of three exemplary patients (no. 3, 13, and 19) treated by FLOT (3), FLOT (13), and FOLFIRINOX (19). Data points (blue dots) and model simulation (solid orange curves) are shown. Grades of respective adverse effects are provided by colored lines. *Pt* Platelets, *ANC* Absolute neutrophil count, *Leuko* Leukocyte count

Parameter estimation resulted in a good fit of model and data for almost all patients. Parameters are listed in the Tables S1 of Supporting information. They were well identifiable with a relative standard error not exceeding 50% (estimated individual parameters are not presented in this paper).

### Agreement of observed and predicted platelet counts, neutrophils, and white blood cells

**Fig. 3:**
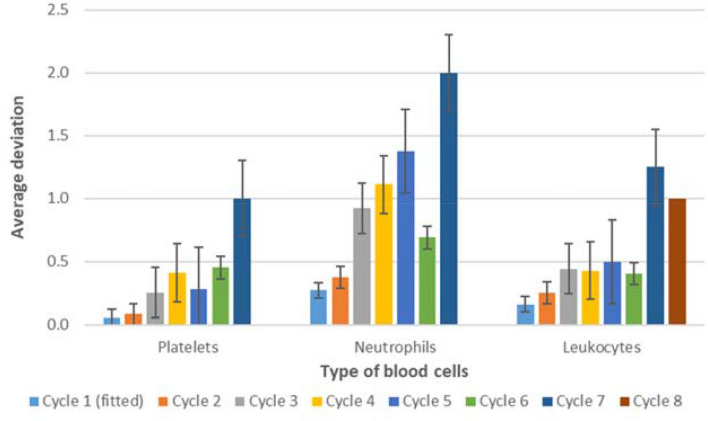
Average deviation of predicted and observed toxicity grades per chemotherapy cycle. We present average values and their standard errors. Cycle 1 was fitted and is shown for comparison only

Figure 3 presents the average deviation of the toxicity level predicted by the biomathematical model from the actually observed toxicity level for each chemotherapy cycle and blood cell type. Prediction agreements for early therapy cycles were excellent throughout. As expected, at later therapy cycles, larger deviations between predicted and observed toxicity levels occurred. Prediction accuracies for the different cell types were not uniform. Platelet toxicity grades were well predicted up to cycle eight. The average deviation over all cycles was 0.32 grades with a maximum deviation of one grade in cycle 7. Predictions regarding neutropenia and leukopenia grades showed larger deviations. For cycles two, three, and six, the average deviation was less than one grade. Larger deviations were observed for cycles four, five, and seven. Leukopenia levels could be predicted up to cycle six with a deviation of less than one toxicity degree on average. Only the predictions for cycles seven and eight deviated by more than one toxicity grade on average. Thus, prediction performances for leukocytes and platelets were superior to that for neutrophils. Of note, neutrophils also showed the largest deviations during parameter fitting of first-cycle data.

**Fig. 4:**
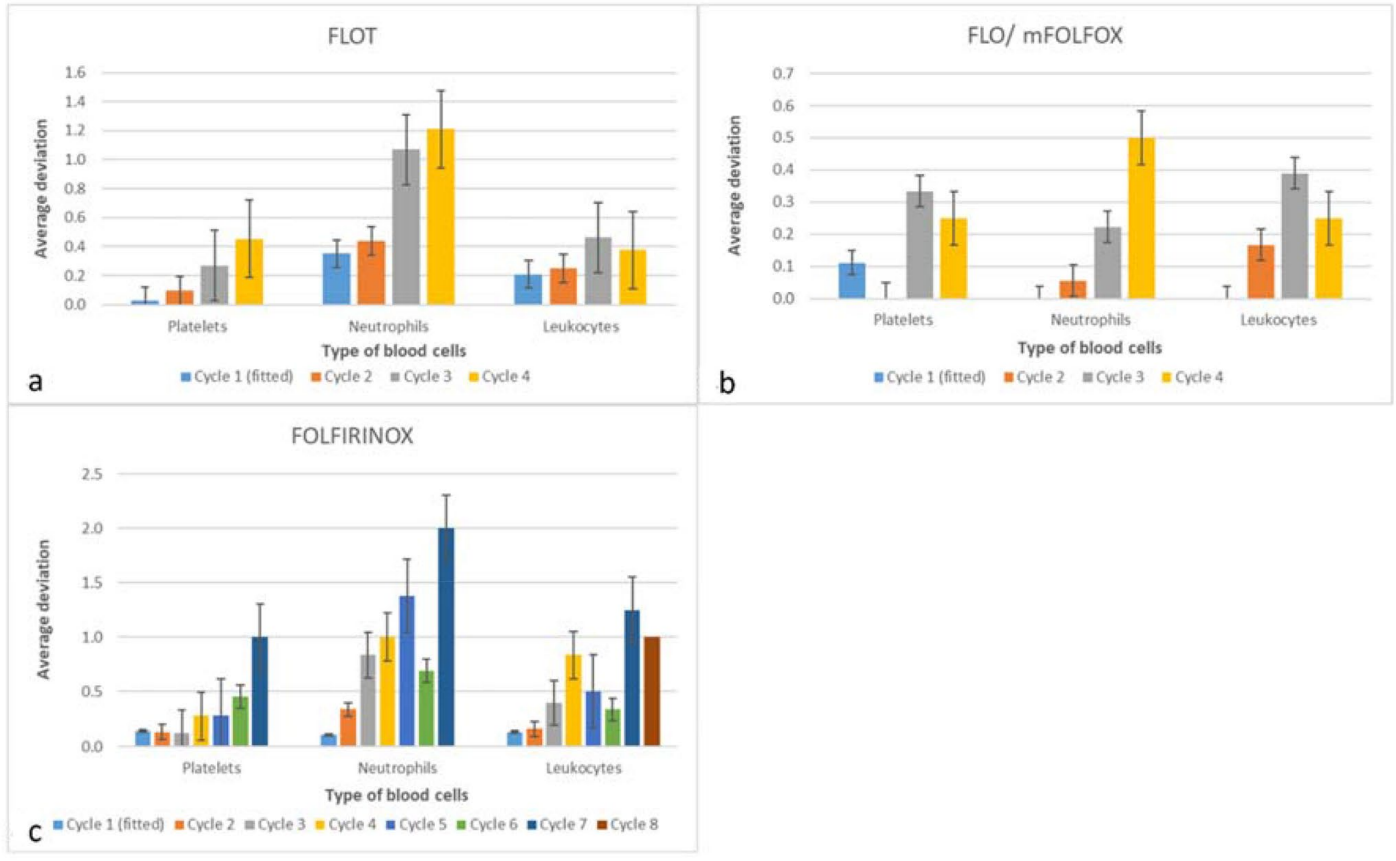
Average deviation of predicted and observed toxicity grades per therapy and treatment cycle. We present average values and their standard errors. Cycle 1 data were used for individual parameter fitting. Note that different ranges of the y-axes were implemented to improve readability of the figure. Predictions for the FLO/mFOLFOX regimen were good for all blood cell types (**b**). However, this treatment group did not develop high-grade hematological adverse events

When comparing prediction accuracy between therapeutic regimens, a few differences were observed (Fig. 4a–c). Figure 4a shows the prediction accuracy for the four preoperative FLOT therapy cycles. Thrombopenia and leukopenia could be predicted with a deviation of less than one toxicity grade on average. The prediction accuracy for neutrophils was also less than one degree on average for the second cycle but was larger for cycles three and four.

In patients with pancreatic cancer treated with FOLFIRINOX (Fig. 4c), thrombocytopenia was accurately predicted up to cycle six, with an average deviation of less than one degree. For cycle seven, the degree of thrombocytopenia was predicted with a one-degree deviation, and in cycle eight, the predicted degree of thrombocytopenia was in perfect agreement with the clinically measured degree, but only one patient was available here. Neutropenia grades were predicted with an accuracy of less than one-degree deviation on average for cycles two, three, and six. For cycles four, five, and seven, the average deviation was between one and two degrees of toxicity. No neutrophil values were available for cycle 8. In the seventh and eighth cycles, the average deviation was one degree or more.

### Correlation between individual model parameter estimates and clinical risk factors

No correlations between individual model parameters and patient’s age or BMI were found. In contrast, significant correlations could be detected between BSA and ${b}_{S\_act}$ (sensitivity parameter of G-CSF action on stem cells, *ρ* = 0.381), *w*_*PLC*_ (maximum TPO elimination rate by circulating platelets, *ρ* = 0.404), ${PD}_{\mathrm{oxali}}$ (PD effect of Oxaliplatin, ρ = -0.443), ${b}_{{A}_{CM}}$ (sensitivity parameter of G-CSF action on CMP and CM progenitor cells, *ρ* = 0.505), ${d}_{{\mathrm{Osteo}}_{\mathrm{loss}}}$ (elimination rate of dormant precursors (other than megakaryocytes) due to lack of osteoblast support, *ρ* = − 0.522), and ${b}_{{MKC}_{p,\mathrm{64,1}}}$ (sensitivity parameter of TPO action on transition from MKC sub-compartment of ploidy 64 to the proplatelets compartment, *ρ* = 0.482). Of note, no correlations between BSA, which is used to normalize chemotherapy dosages, and minimum counts of neutrophils, leukocytes, or thrombocytes were observed.

## Discussion

Neoadjuvant and adjuvant cytotoxic chemotherapy improves the prognosis of patients with GI cancers but causes toxicities at the same time, which affect the hematopoietic system in particular. Hematotoxicity is common but highly heterogeneous across patients. Novel strategies for individualized treatment tailoring are required to optimize the effectiveness of chemotherapy and limit toxicity to an acceptable level. Predicting side effects on individual levels is a difficult task due to the complexity and strong dynamic interactions between cytotoxic drugs and endogenous or applied growth factors combined with the rapid turnover of blood cells. Therefore, intelligent prediction models are required to capture essential biological features of these processes (Kheifetz and Scholz 2019, 2021). To achieve this goal, the predictive potential of our formerly developed modeling approach was investigated in the present study. For the first time, this model was studied in a patient population diagnosed with GI cancers receiving FLO(T) or FOLFIRINOX chemotherapy. Single patient data are too sparse to learn a model. To cope with this issue, we invented the principle of virtual participation reinforcing learning by other data resources by penalizing deviations from these data (Kheifetz and Scholz 2019, 2021). This approach, in principle, allows its implementation in clinical practice where the task is to decide on chemotherapy treatment continuation at the same dose, dose adjustments, or treatment postponement for a specific patient in real time. This decision problem is of great importance for the treatment success and the prognosis of the individual patient.

Despite the inter-individual variability among the patient diagnoses and characteristics for the applied treatment regimens (FLO(T)/FOLFIRINOX), we successfully fitted dynamics of platelets, leukocytes, and neutrophils of first-cycle data with our model. Significant correlations between few model parameters and patient covariates were found, implying a potential to increase the predictive performance of the model in the future by including baseline risk factors.

Short-term (i.e., next or next two cycles) prediction performance of the model was excellent for all considered blood cell lines and therapies. At later cycles, the prediction performances drop, in particular for very late cycles, which is not unexpected, because uncertainties in model parameter estimation result in accumulating prediction errors over multiple cycles. However, this is of minor practical importance, because the prediction of later chemotherapy cycles could easily be improved by considering all accomplished cycles of a patient (i.e., considering cycles 1–3 to predict cycle 4) as shown in our previous publication (Kheifetz and Scholz 2021). This issue was not investigated in the present study to reduce the complexity of analyses but could be considered in the future. Prediction performance of neutrophils was inferior compared to platelets and leukocytes possibly due to stronger dynamic changes of neutrophils compared to other (white) blood cell lines.

Most of the study patients were treated with perioperative FLOT chemotherapy, as a curative intended treatment regimen for localized gastric or gastro-esophageal cancers. This regimen is expected to cause frequent hematological toxicities. For our prediction model, data of the preoperative chemotherapy cycles were considered. The reasons for this are, on one hand, that not all study participants received postoperative chemotherapy cycles, and on the other hand that it is unclear which effects surgery has on the hematopoietic system and how these can be considered in the biomathematical model. Among the five study, patients who received FOLFIRINOX were three patients who were treated with perioperative chemotherapy. Again, the preoperative cycles were considered, analogous to the FLOT patients. Two patients received FOLFIRINOX in the adjuvant setting. Of these two patients, the data from the postoperative cycles were included in the study, for both individual model parametrization and prediction. Analogous to the perioperative setting, the data from the first administered chemotherapy cycle served as the basis for the predictions.

Limitations of this study are the small number of cases and the heterogeneity of our patient population in terms of GI cancer diagnoses and treatment regimens (gastric, gastro-esophageal, and pancreatic cancer; FLO(T)/FOLFIRINOX). However, all patients presented with non-metastatic disease and were treated in a curative setting, which decreases the variability of baseline characteristics. Despite the limitations and with all constraints, we could demonstrate that the application of the biomathematical prognosis model to predict individual hematological toxicity is feasible and leads to reliable results even for this heterogeneous real-world population. In view of these promising observations, we suggest that the suitability of the model should be verified in large prospective studies.

## Conclusion

As a quantitative method, a biomathematical prediction model showed excellent short-term and decent long-term prediction performance for all relevant hematological side effects associated with FLO(T)/FOLFIRINOX chemotherapy in patients with localized and curatively treated GI cancers. The clinical application of this precision-medicine approach is intriguing but needs to be further validated in larger patient cohorts with chemotherapy-treated GI cancers to verify its usefulness in clinical decision-making and precision-medicine applications.

## Supplementary Information

Below is the link to the electronic supplementary material.

Supplementary file1 (PPTX 539 KB)

Supplementary file2 (PPTX 628 KB)

Supplementary file3 (DOCX 68 KB)

## Data Availability

The datasets generated during and/or analysed during the current study are available from the corresponding author on reasonable request.

## Abbreviations

AEG: Adenocarcinomas of the gastro-esophageal junction
ANC: Absolute neutrophil count
BMI: Body Mass Index
BSA: Body Surface Area
CBF: Campus Benjamin Franklin
CCCC: Charité Comprehensive Cancer Center
CCM: Campus Charité Mitte
CM: Megakaryocyte Precursors
CMP: Active Common Myeloid Progenitor Cells
CTCAE: Common Terminology Criteria for Adverse Events
CVK: Campus Virchow Clinical Center
ECOG Performance Status: Eastern Cooperative Oncology Group
GI: Gastrointestinal Cancer
Leuko: Leukocyte count
MKC: Megakaryocytes
Pt: Platelets
TPO: Thrombopoietin
UCCL: University Cancer Center Leipzig
UKL: University Hospital Leipzig
