# Supplementary material for: Individual hematotoxicity prediction of further chemotherapy cycles by dynamic mathematical models in patients with gastrointestinal tumors
Source: J Cancer Res Clin Oncol. 2023 Feb 28;149(10):6989–98. doi: 10.1007/s00432-023-04601-9 (PMC10374676; doi:10.1007/s00432-023-04601-9)
Supplement: Supplementary file 1 — Supplementary file1 (PPTX 539 KB) [file 432_2023_4601_MOESM1_ESM.pptx]

## Slide 1
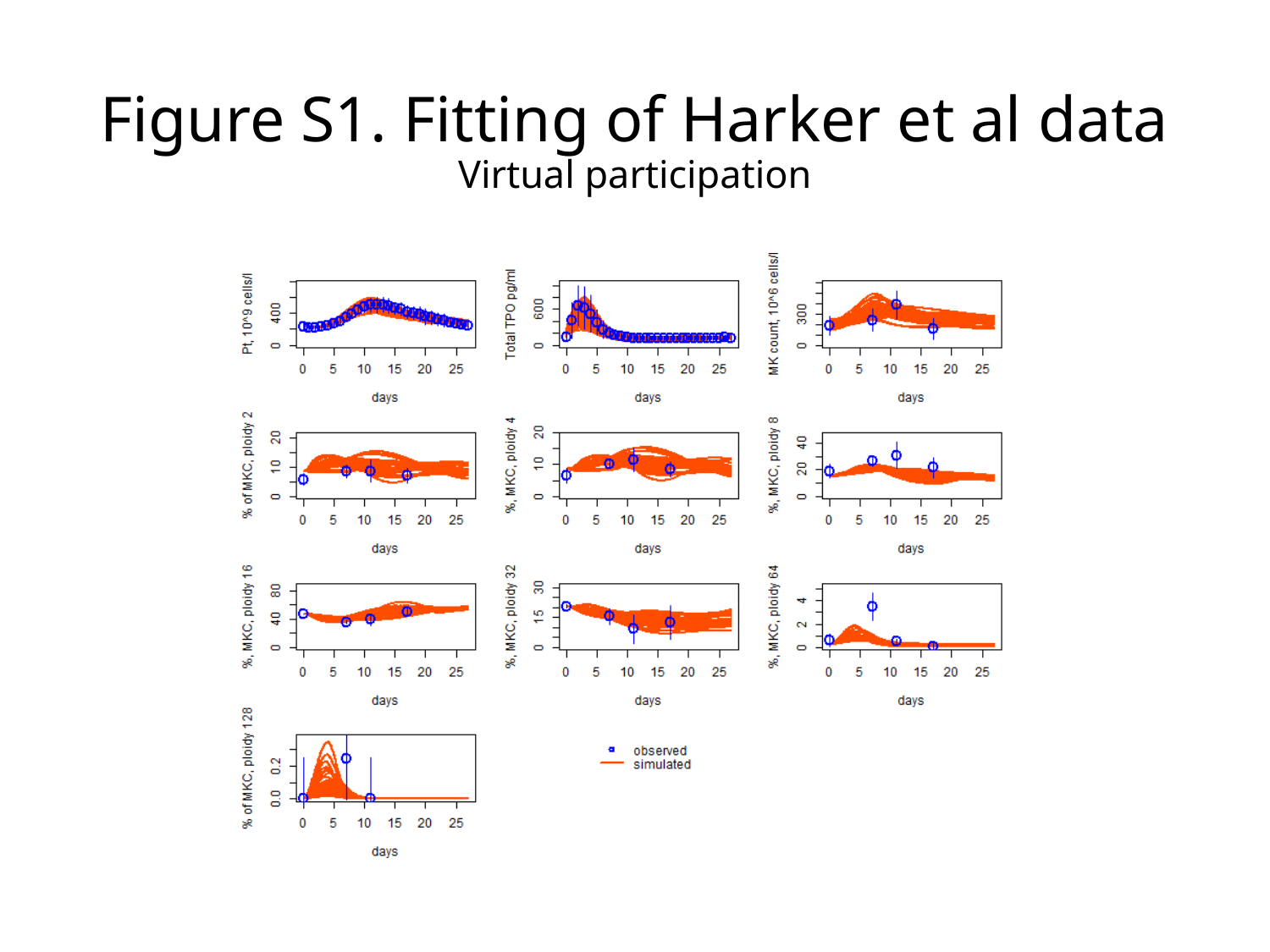

# Figure S1. Fitting of Harker et al data Virtual participation

## Slide 2
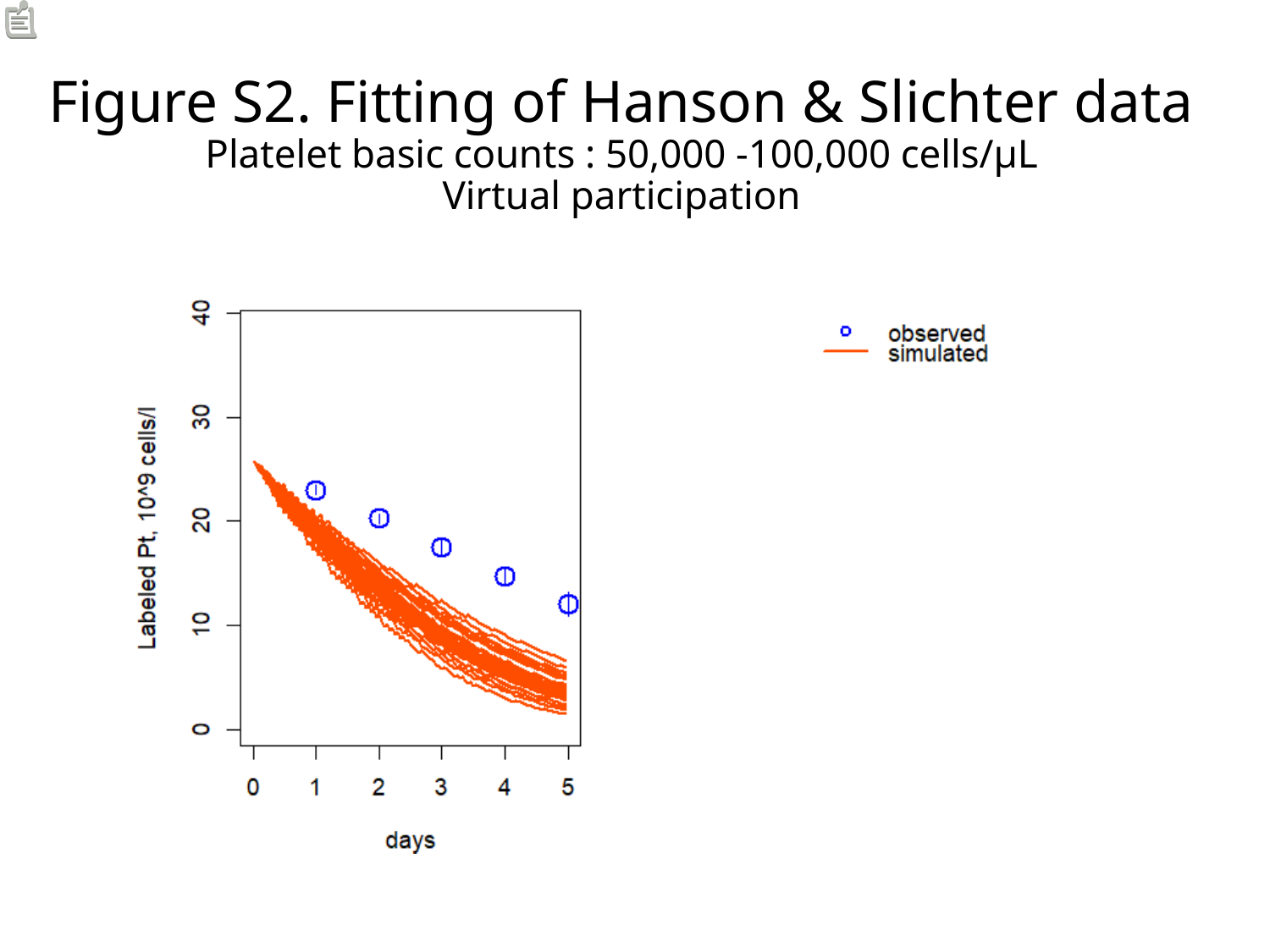

# Figure S2. Fitting of Hanson & Slichter data Platelet basic counts : 50,000 -100,000 cells/µL Virtual participation

## Slide 3
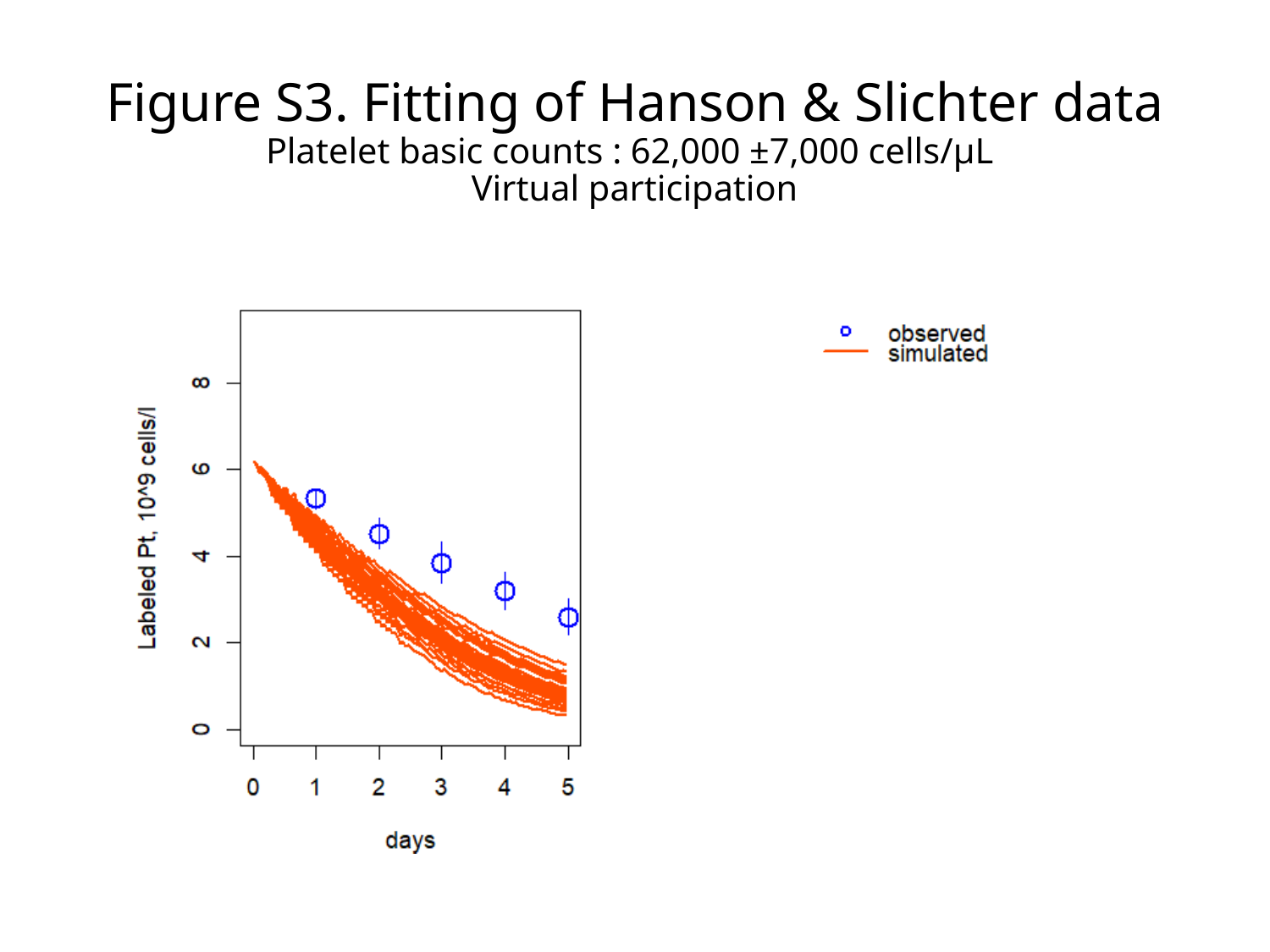

# Figure S3. Fitting of Hanson & Slichter data Platelet basic counts : 62,000 ±7,000 cells/µL Virtual participation

## Slide 4
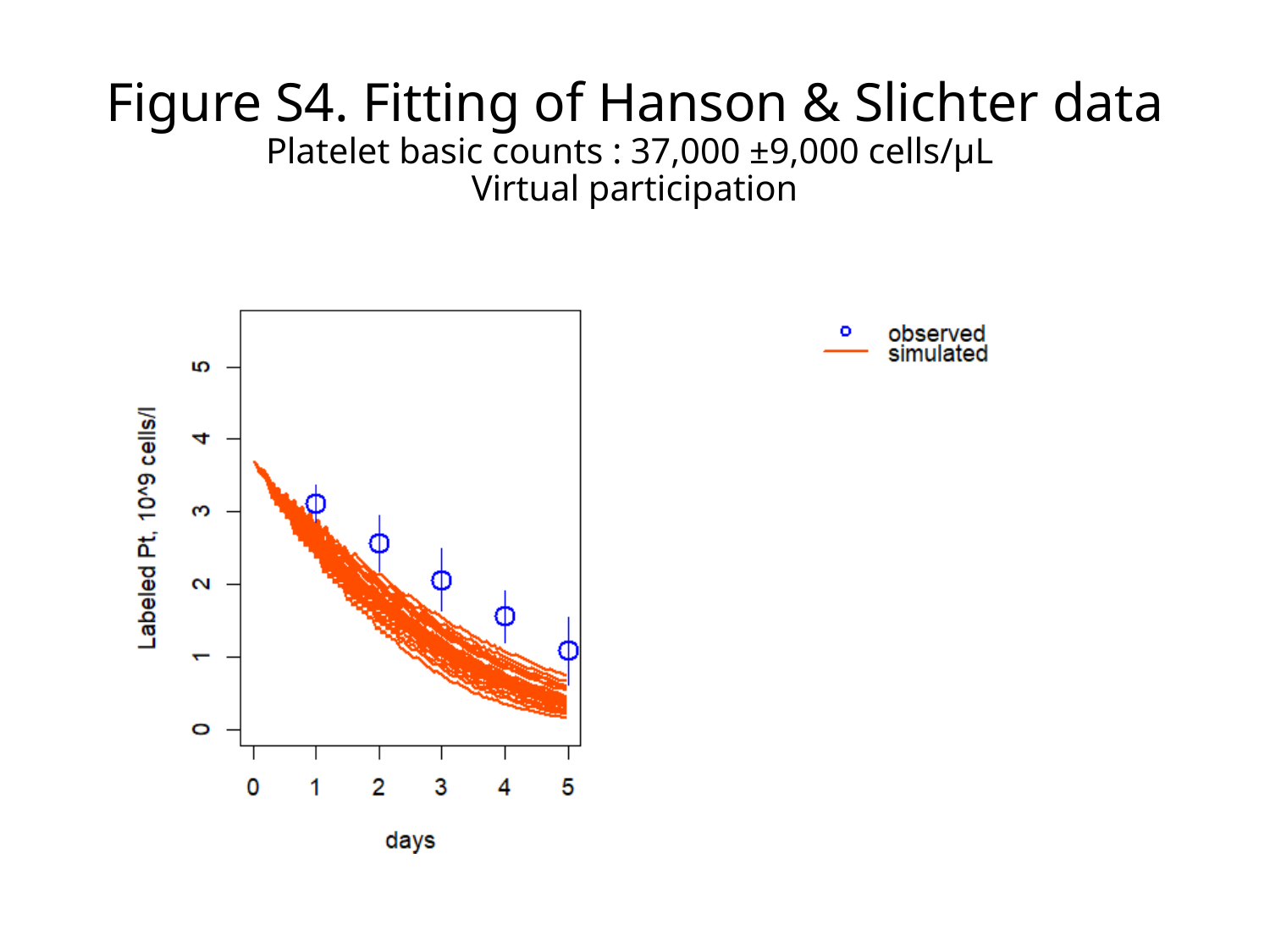

# Figure S4. Fitting of Hanson & Slichter data Platelet basic counts : 37,000 ±9,000 cells/µL Virtual participation

## Slide 5
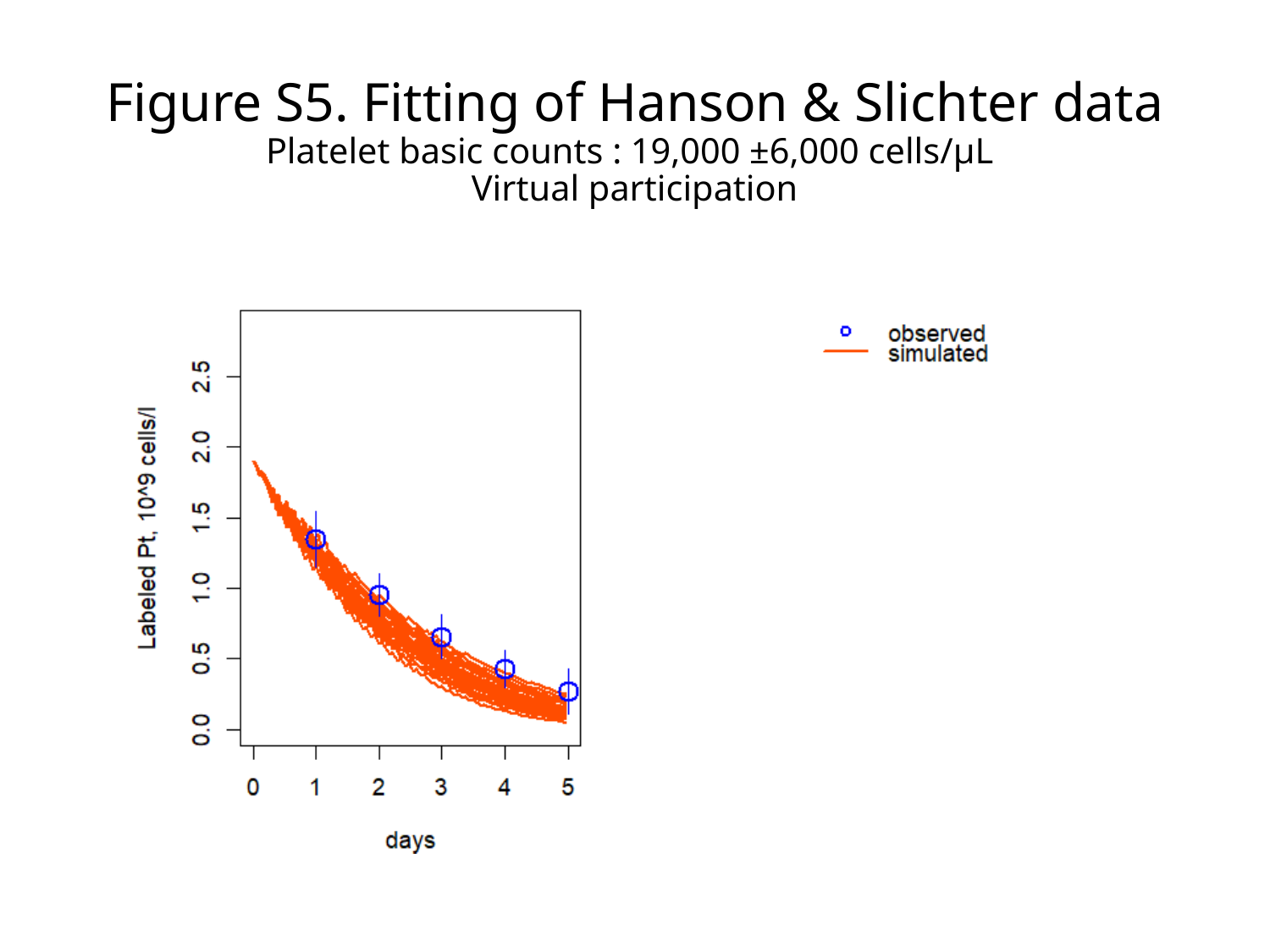

# Figure S5. Fitting of Hanson & Slichter data Platelet basic counts : 19,000 ±6,000 cells/µL Virtual participation

## Slide 6
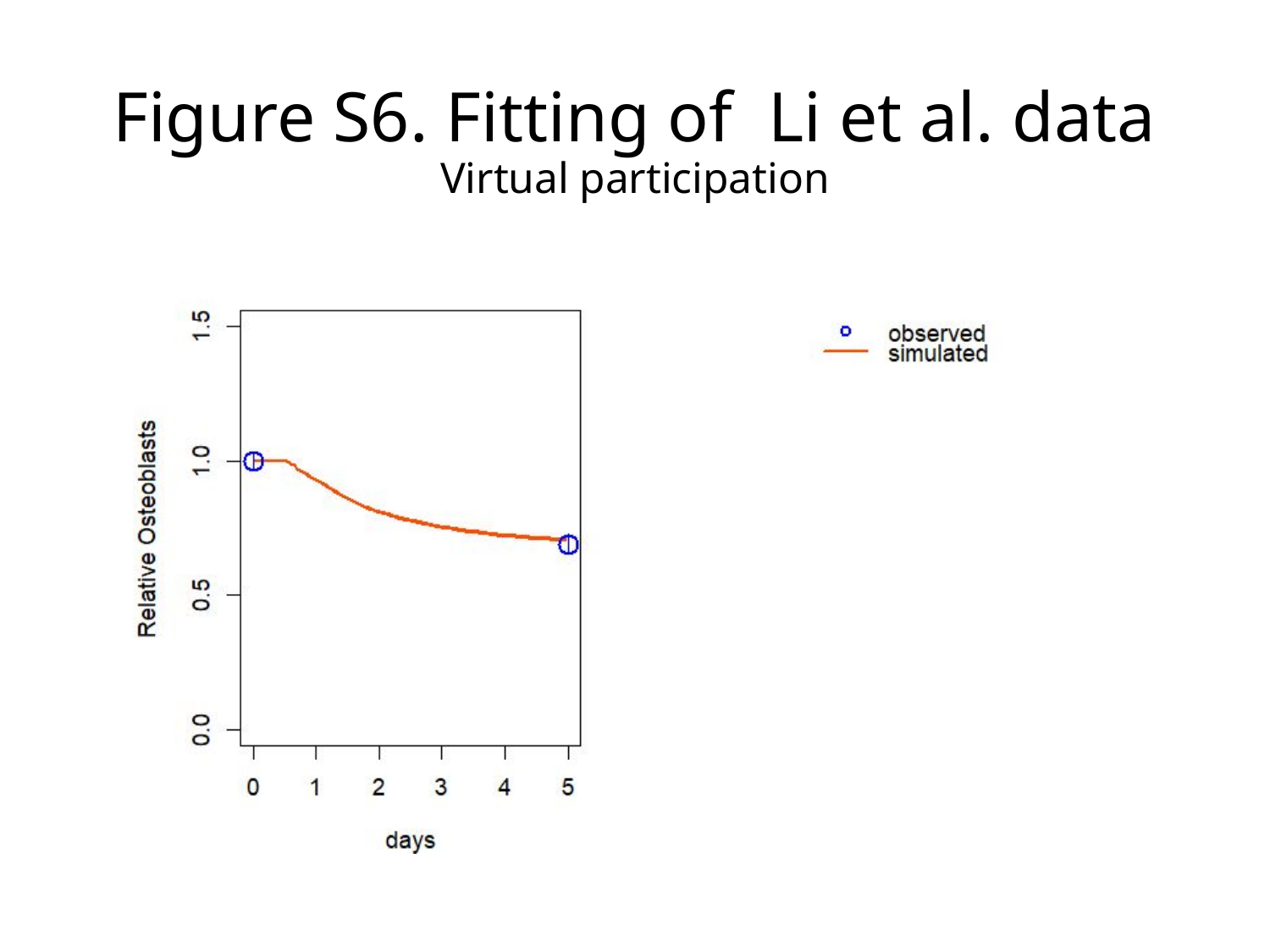

# Figure S6. Fitting of Li et al. data Virtual participation

## Slide 7
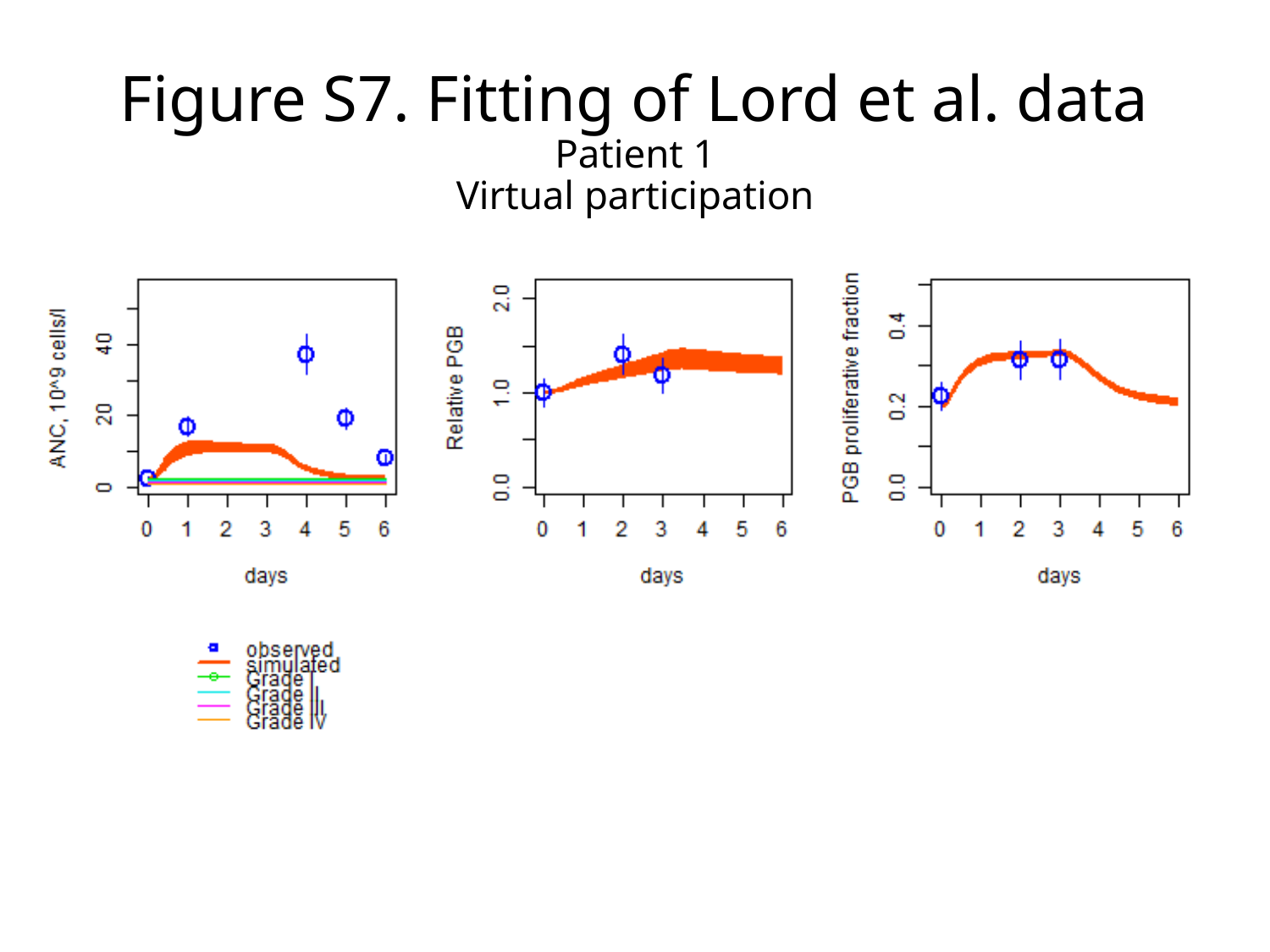

# Figure S7. Fitting of Lord et al. data Patient 1 Virtual participation

## Slide 8
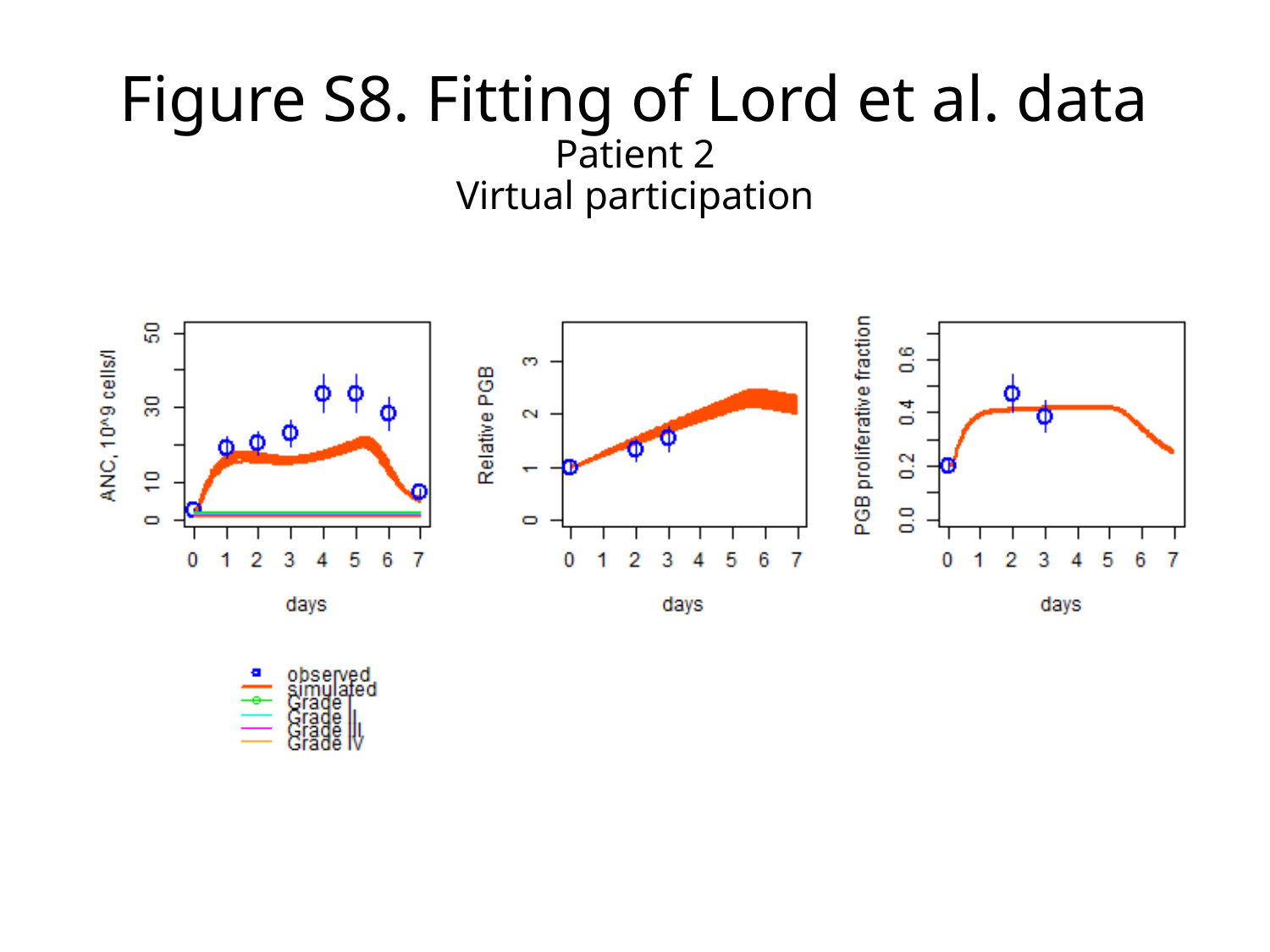

# Figure S8. Fitting of Lord et al. data Patient 2 Virtual participation

## Slide 9
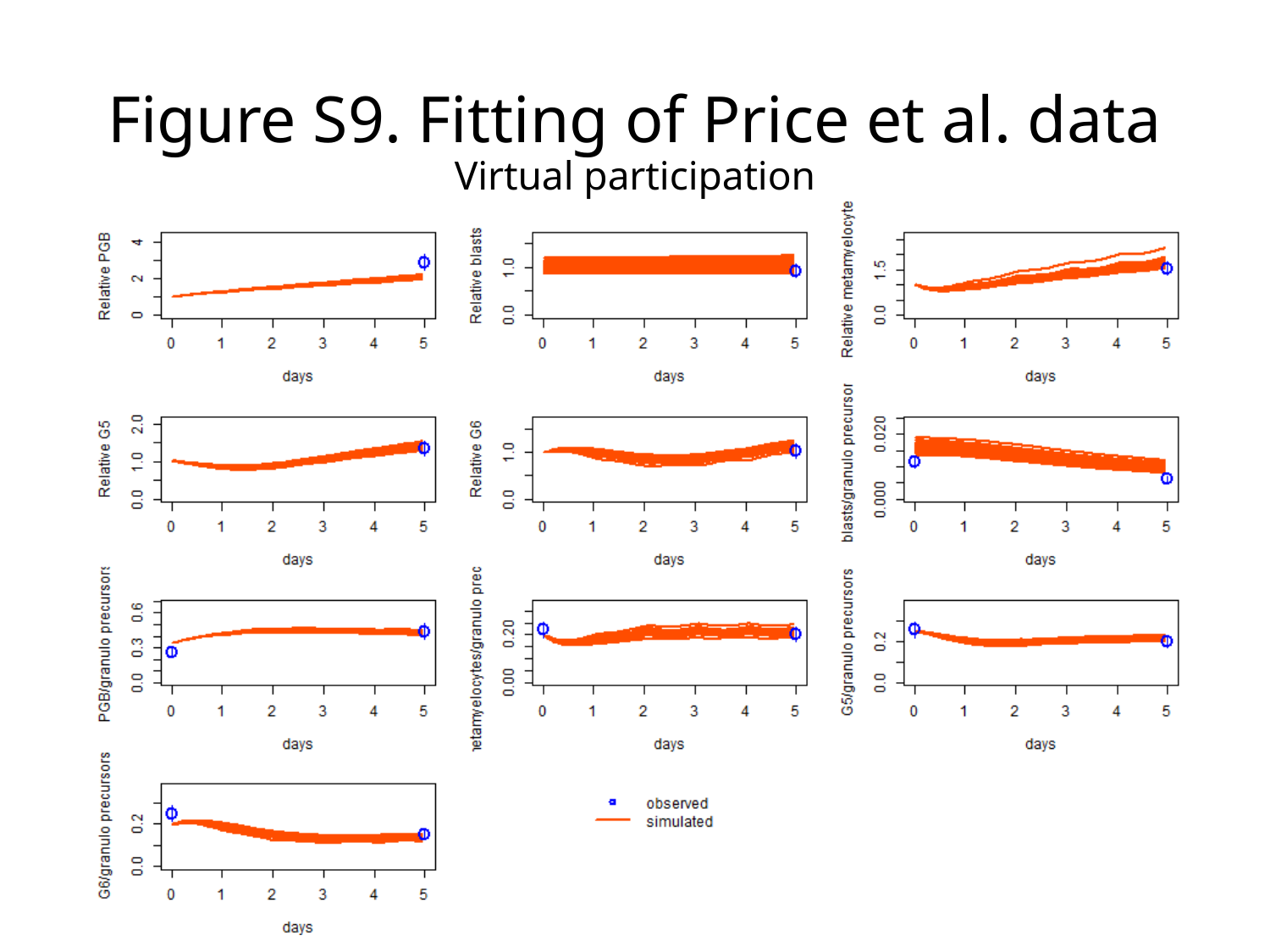

# Figure S9. Fitting of Price et al. data Virtual participation

## Slide 10
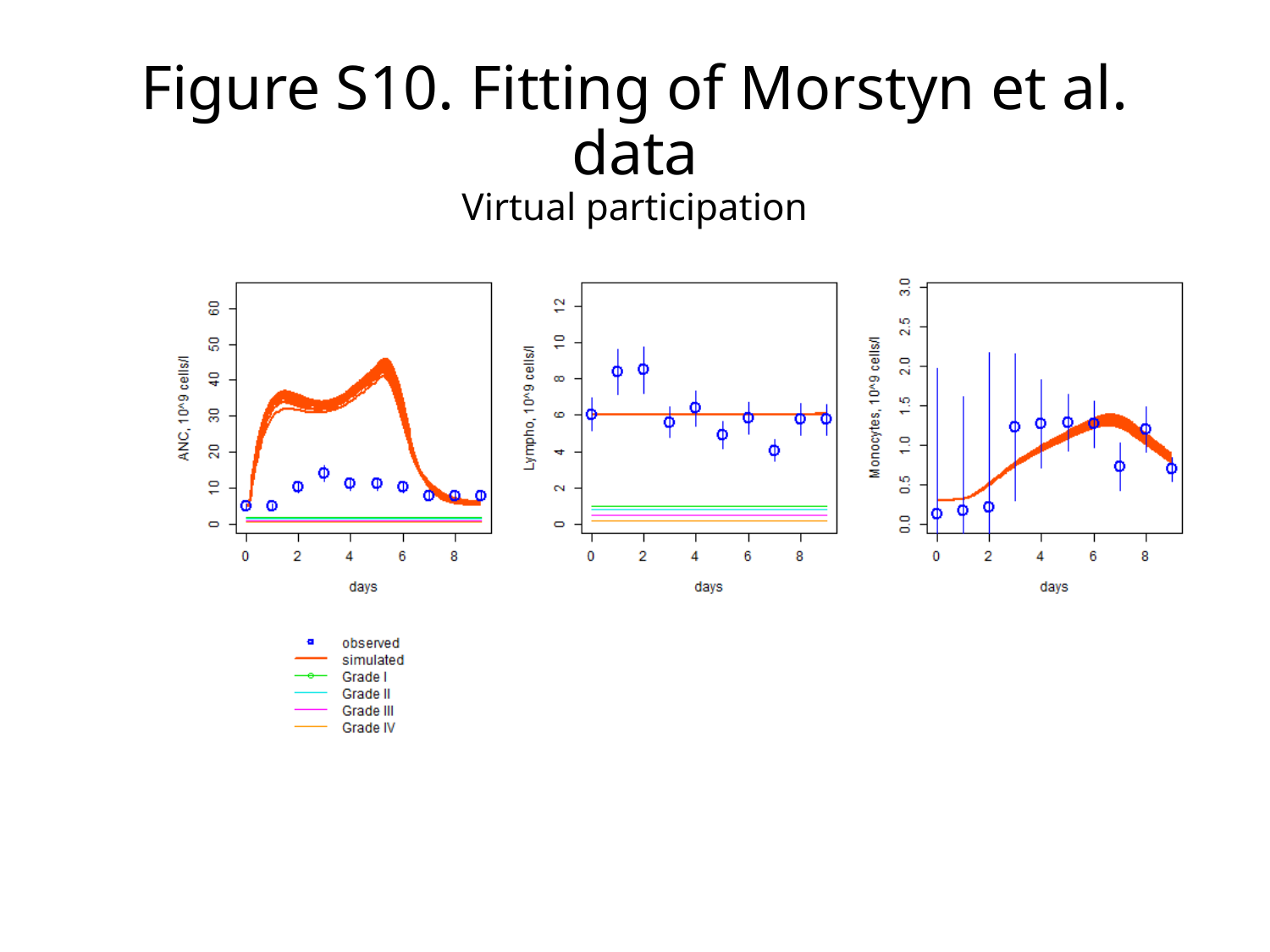

# Figure S10. Fitting of Morstyn et al. data Virtual participation

## Slide 11
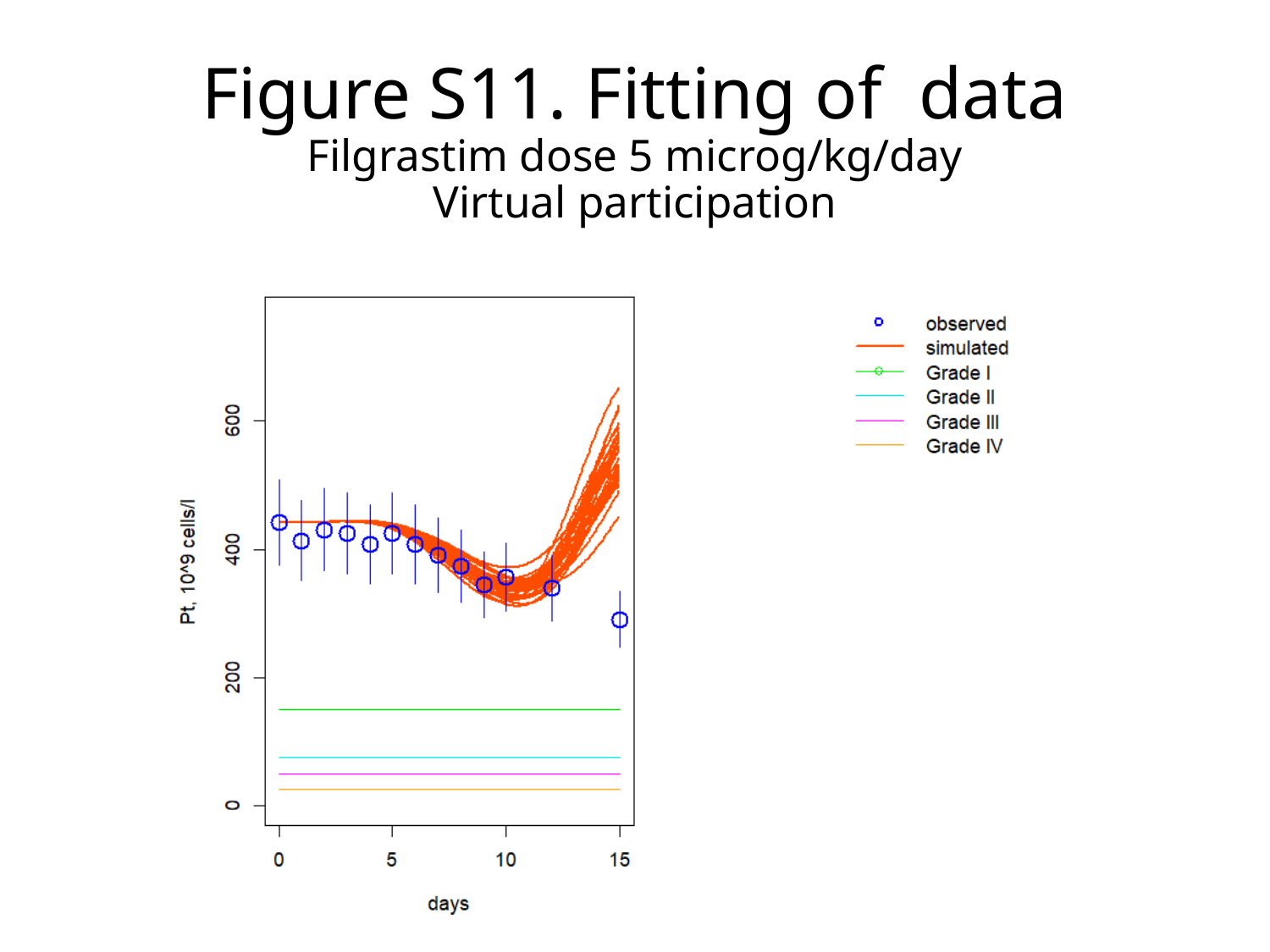

# Figure S11. Fitting of data Filgrastim dose 5 microg/kg/day Virtual participation

## Slide 12
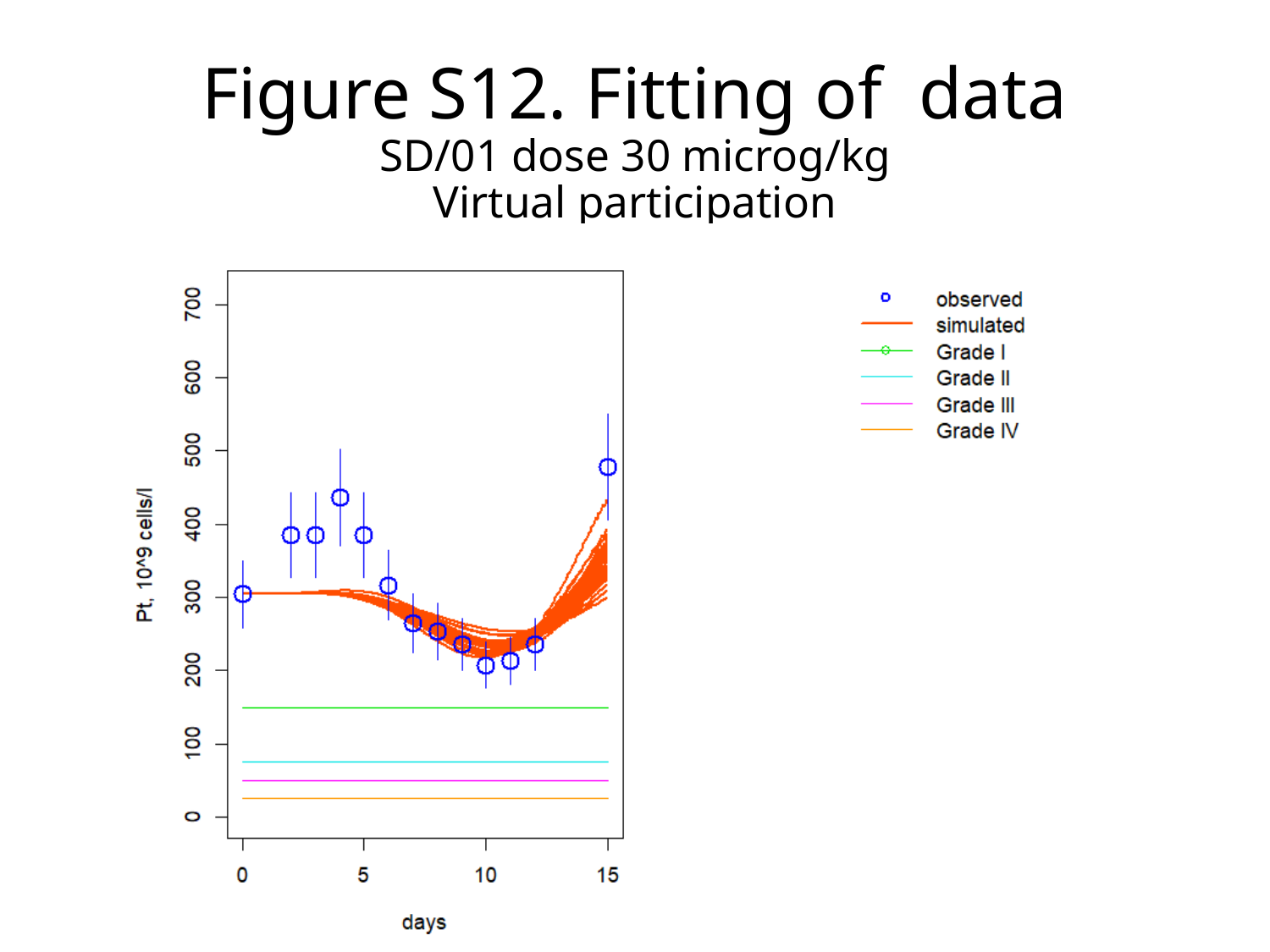

# Figure S12. Fitting of data SD/01 dose 30 microg/kg Virtual participation

## Slide 13
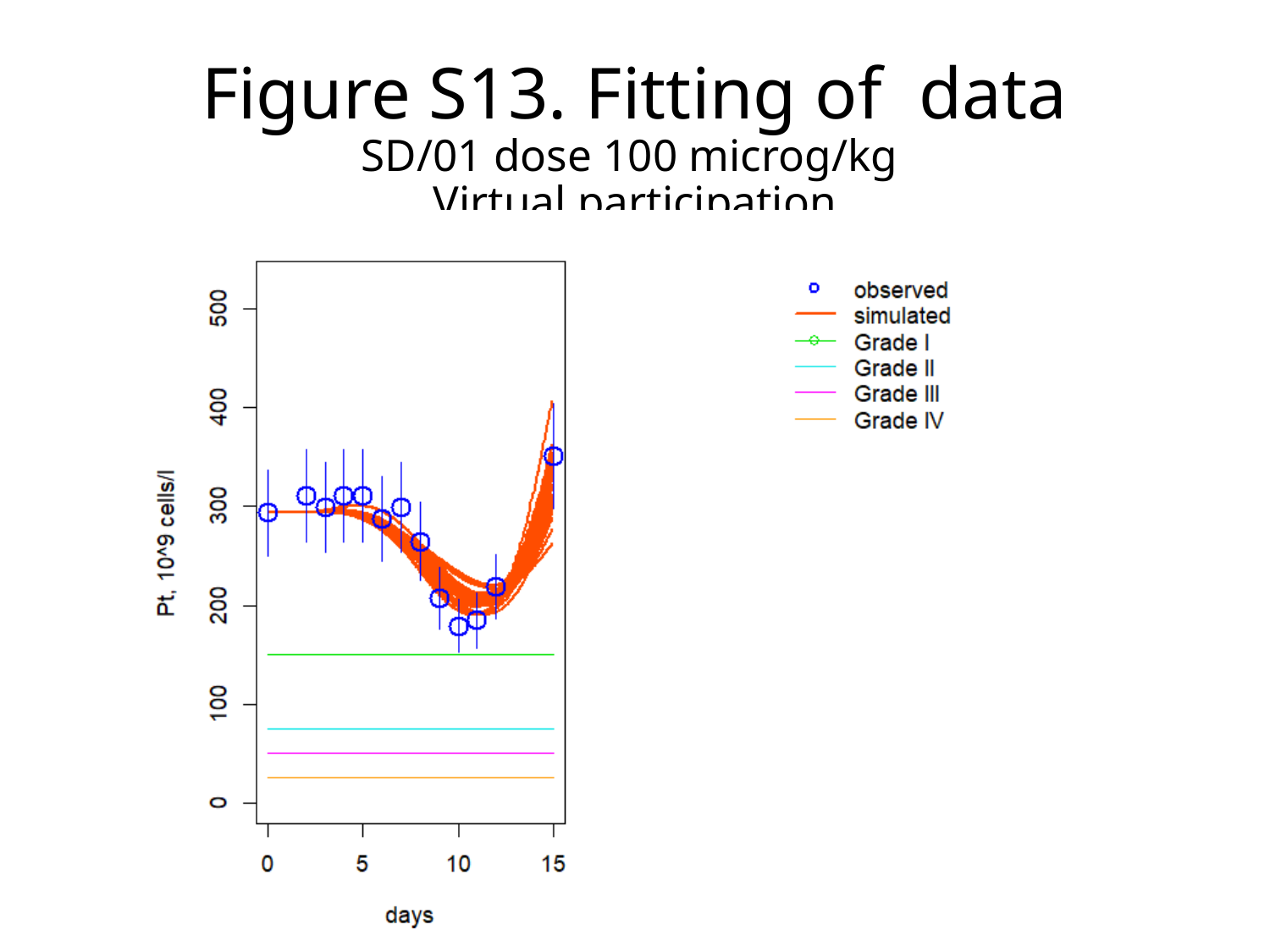

# Figure S13. Fitting of data SD/01 dose 100 microg/kg Virtual participation

## Slide 14
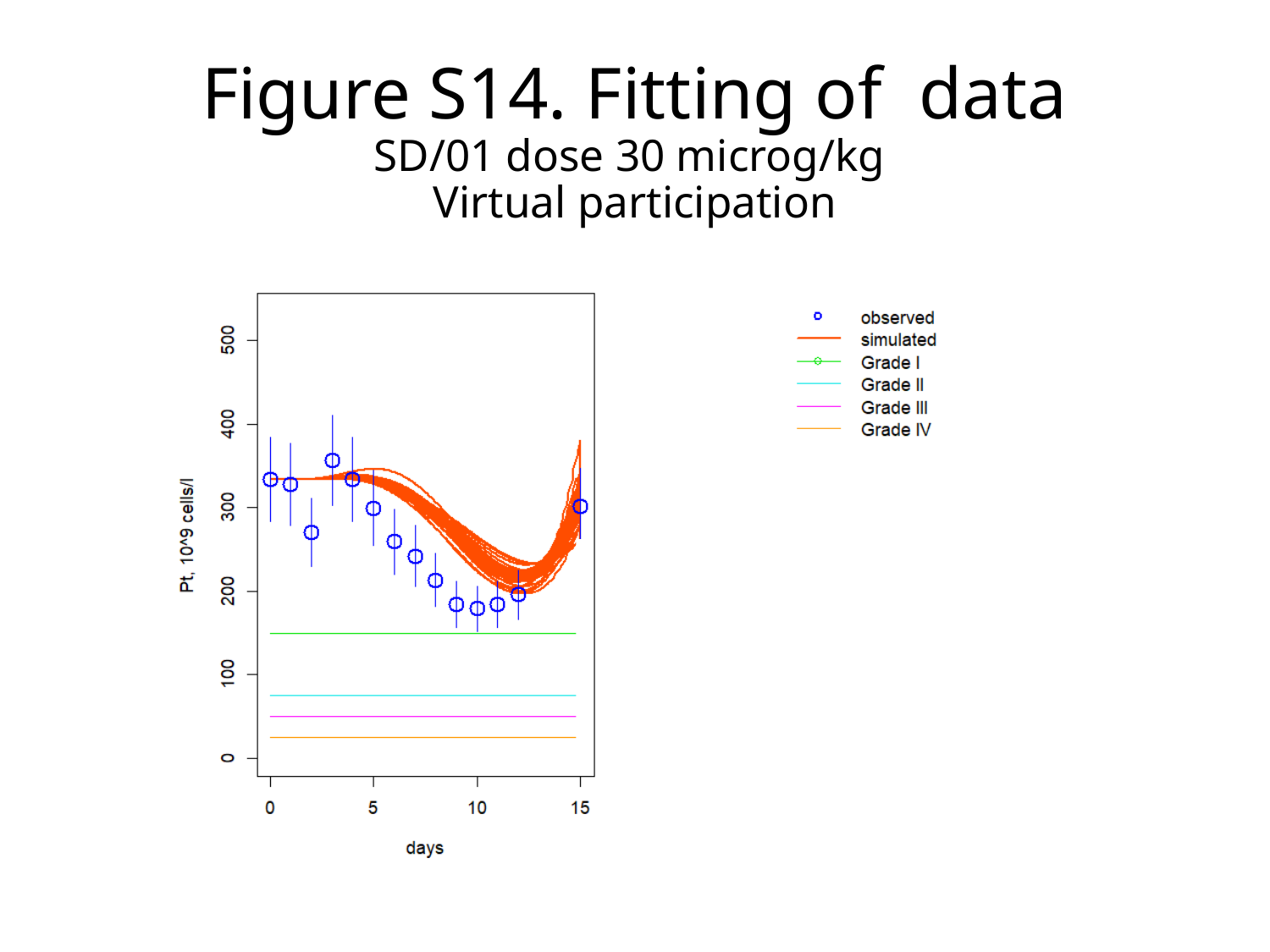

# Figure S14. Fitting of data SD/01 dose 30 microg/kg Virtual participation

## Slide 15
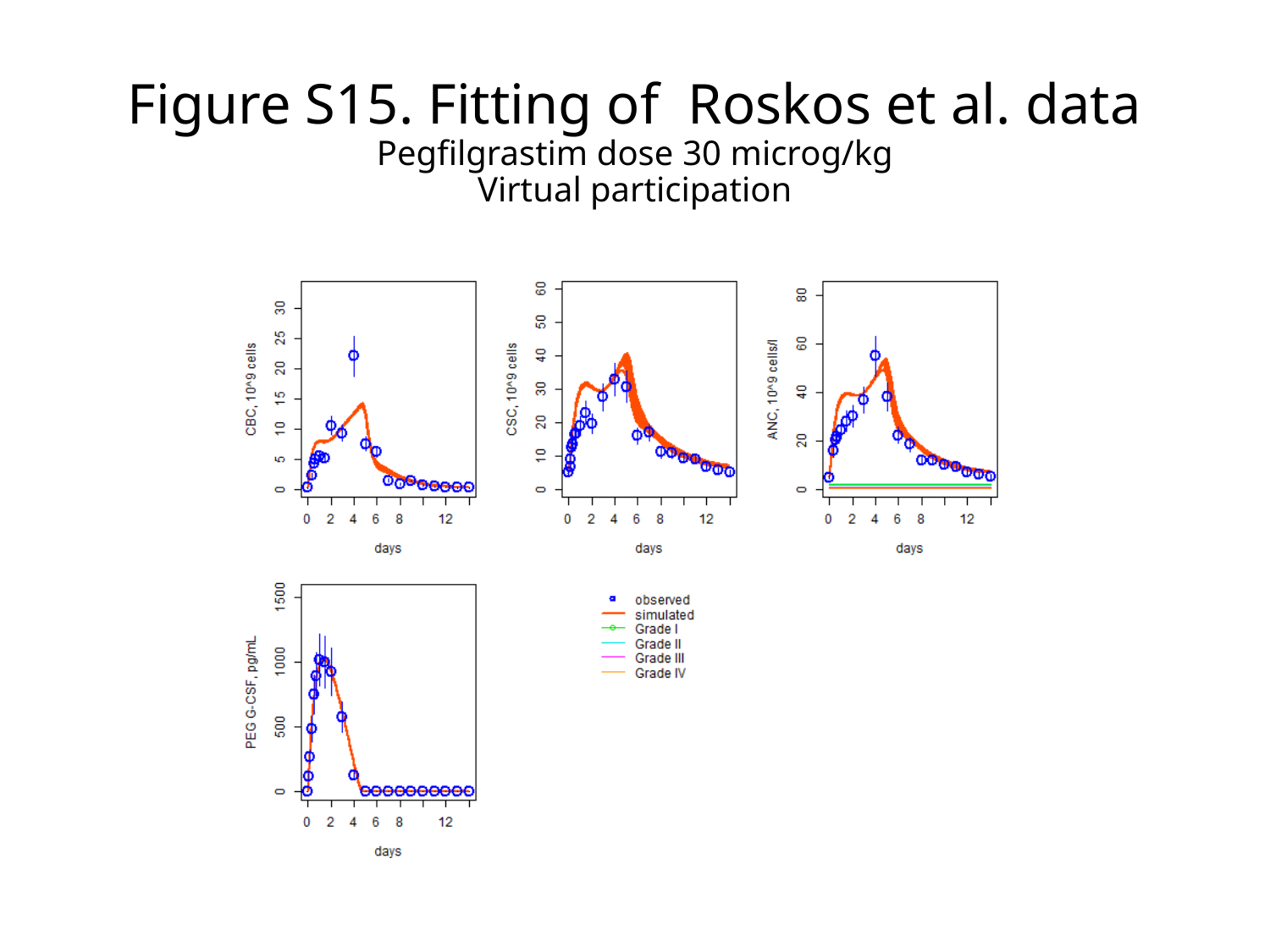

# Figure S15. Fitting of Roskos et al. data Pegfilgrastim dose 30 microg/kg Virtual participation

## Slide 16
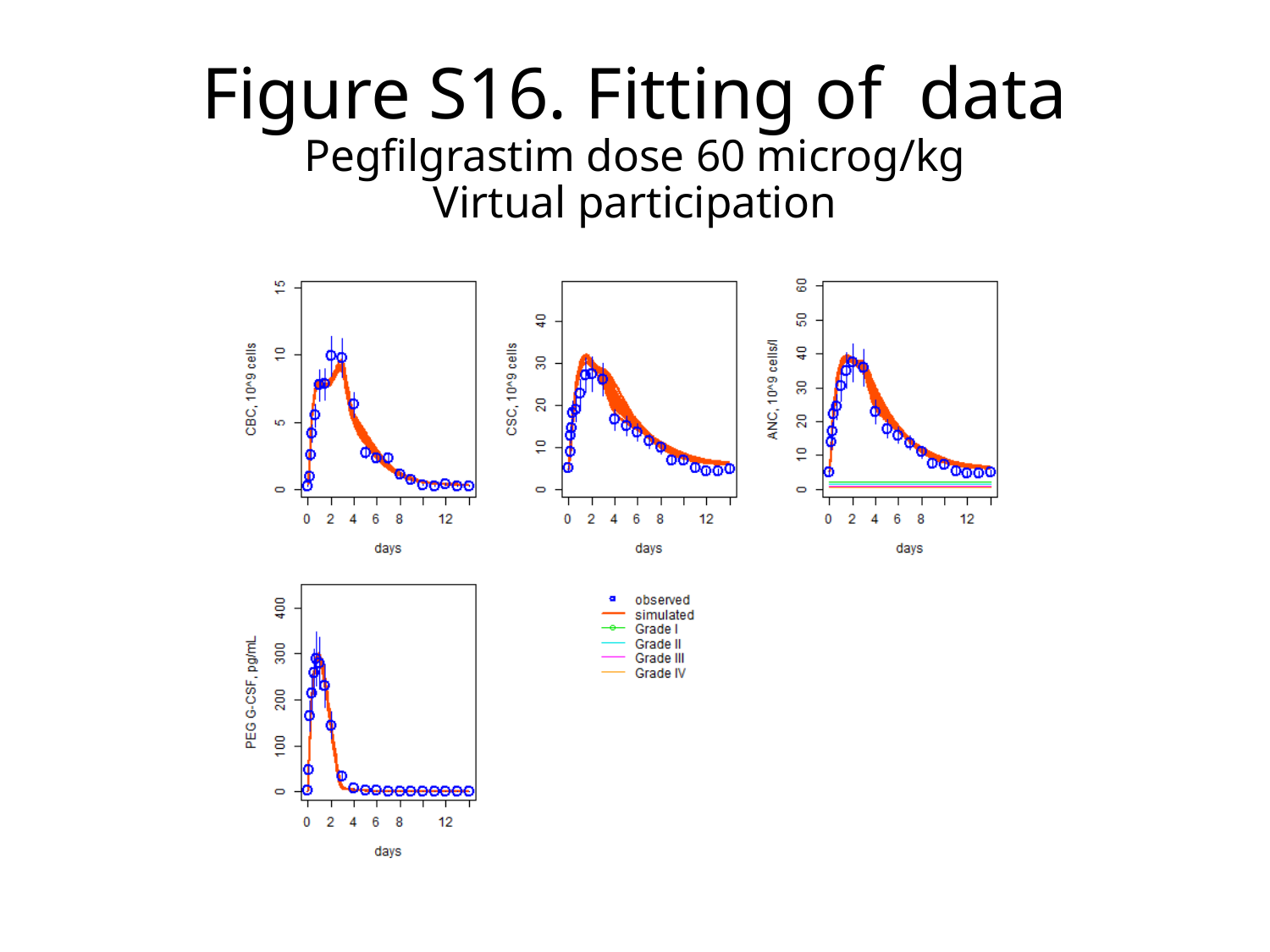

# Figure S16. Fitting of data Pegfilgrastim dose 60 microg/kg Virtual participation

## Slide 17
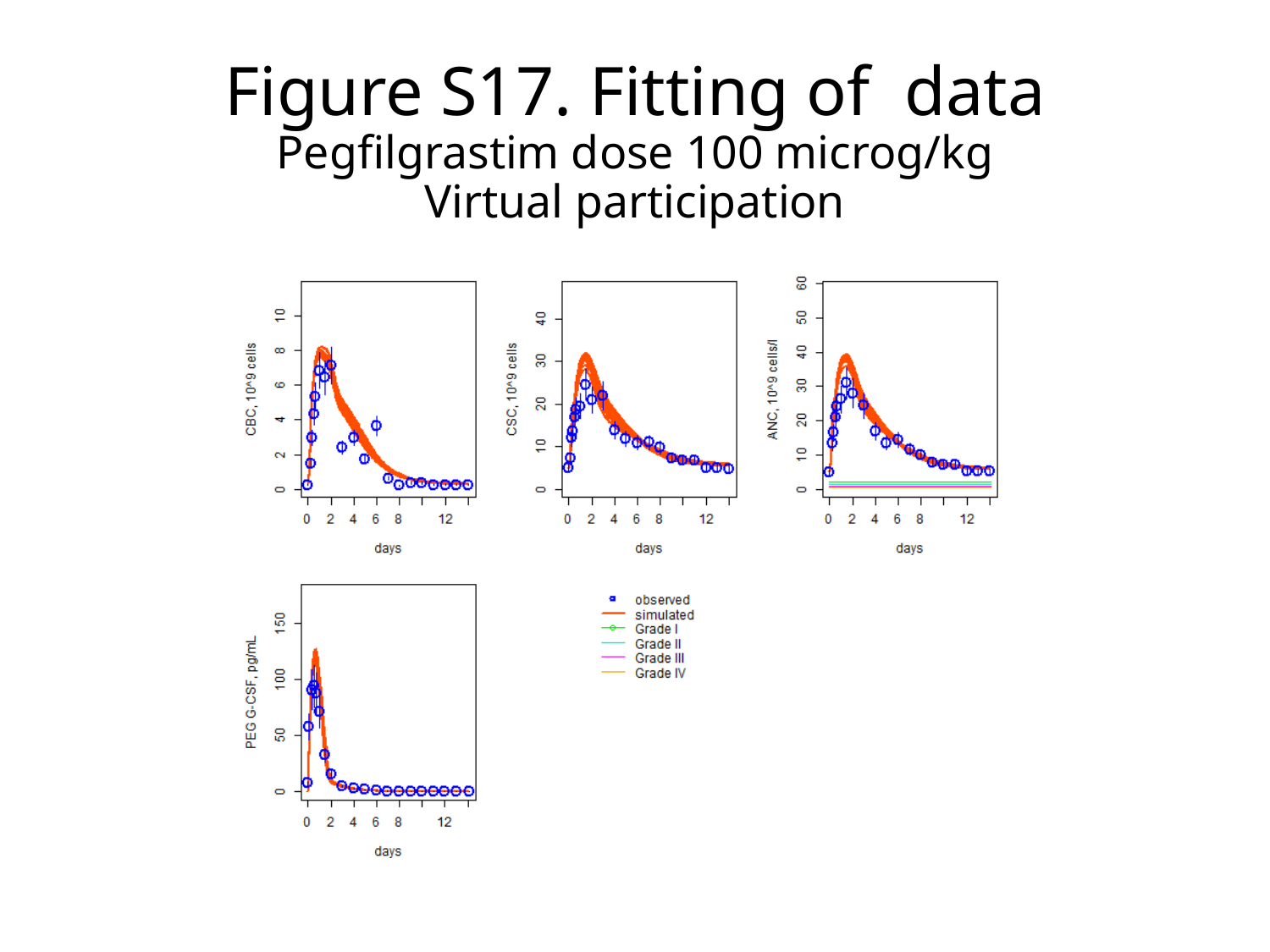

# Figure S17. Fitting of data Pegfilgrastim dose 100 microg/kg Virtual participation

## Slide 18
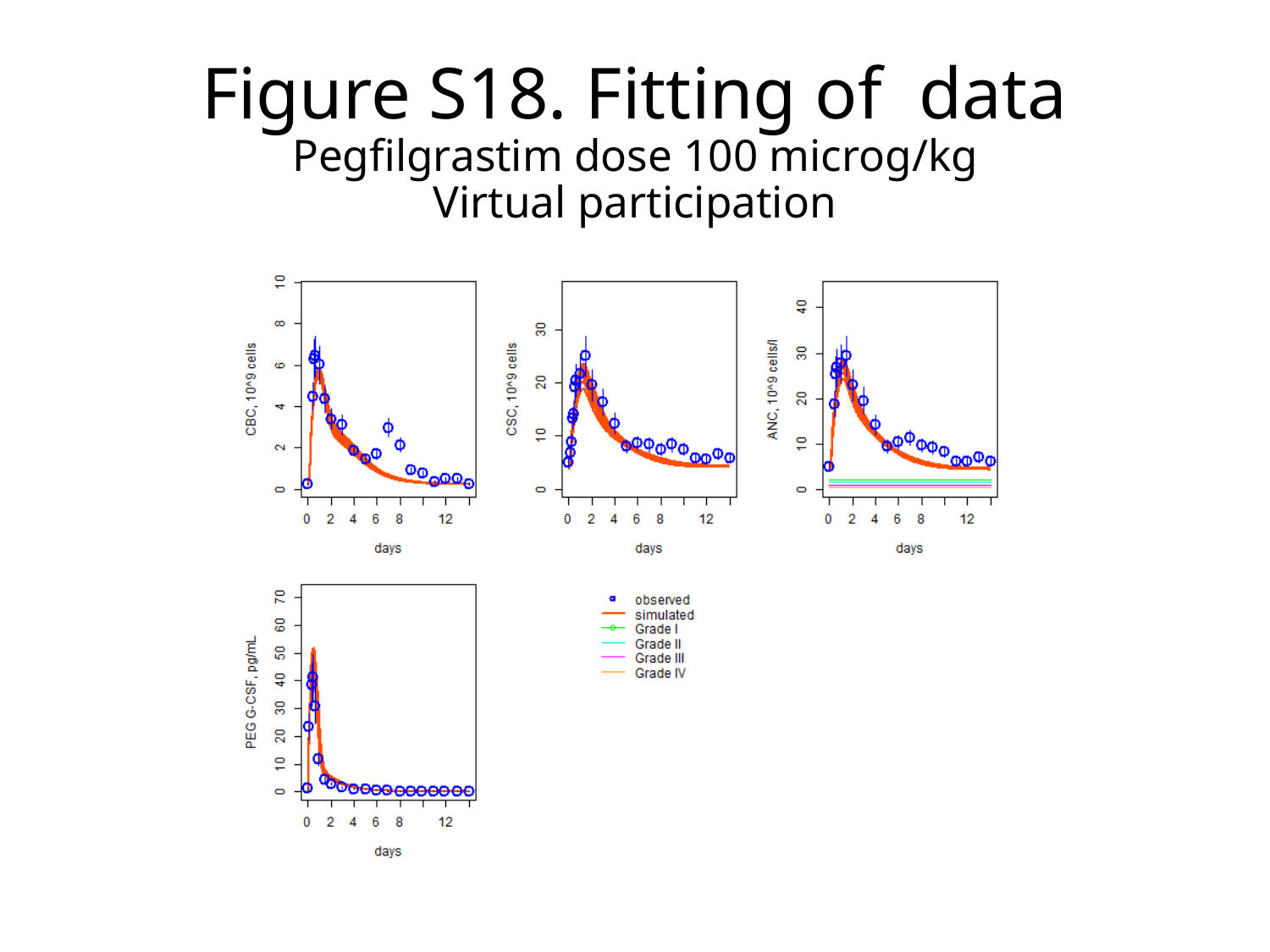

# Figure S18. Fitting of data Pegfilgrastim dose 100 microg/kg Virtual participation

## Slide 19
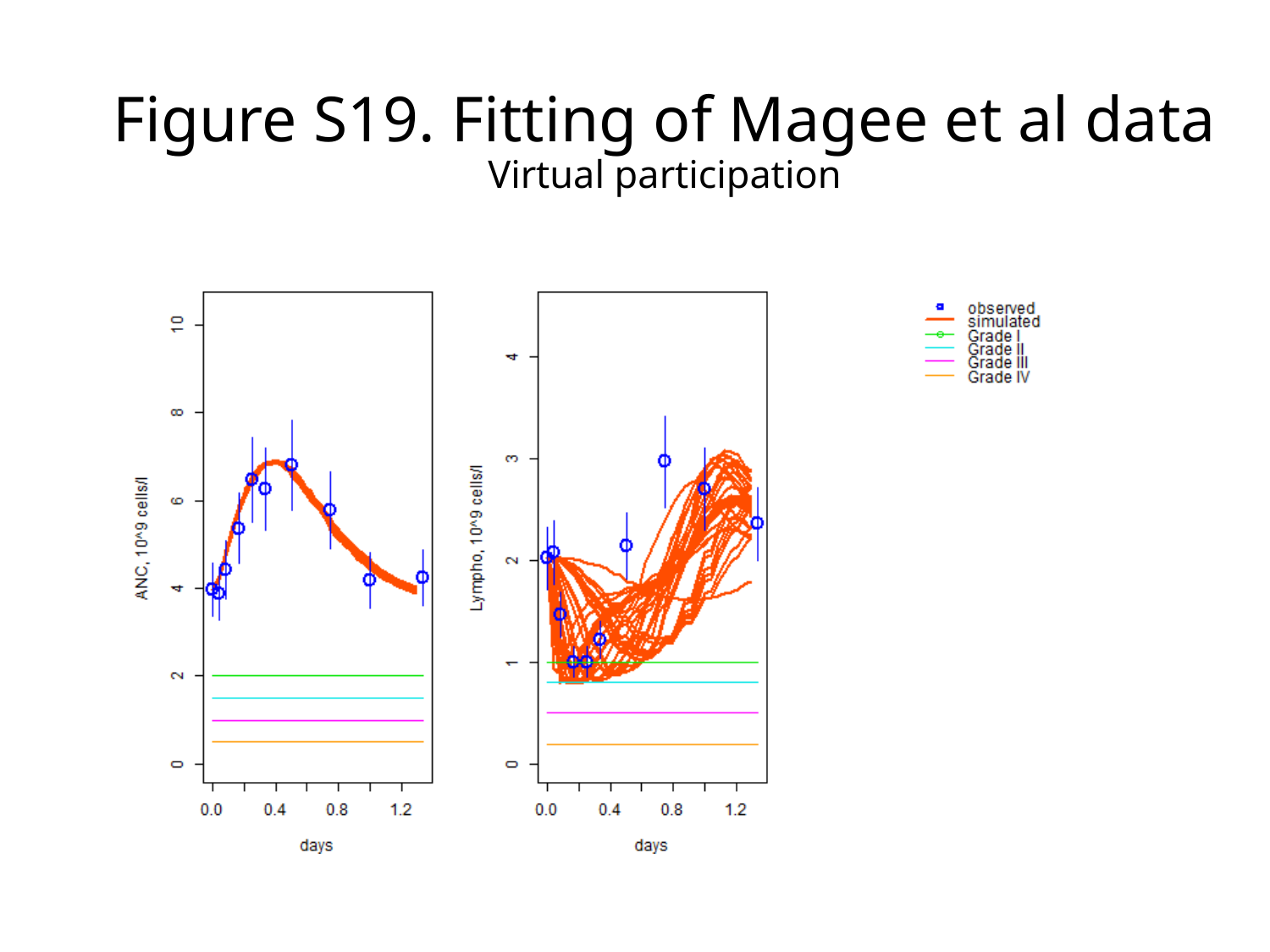

# Figure S19. Fitting of Magee et al data Virtual participation

## Slide 20
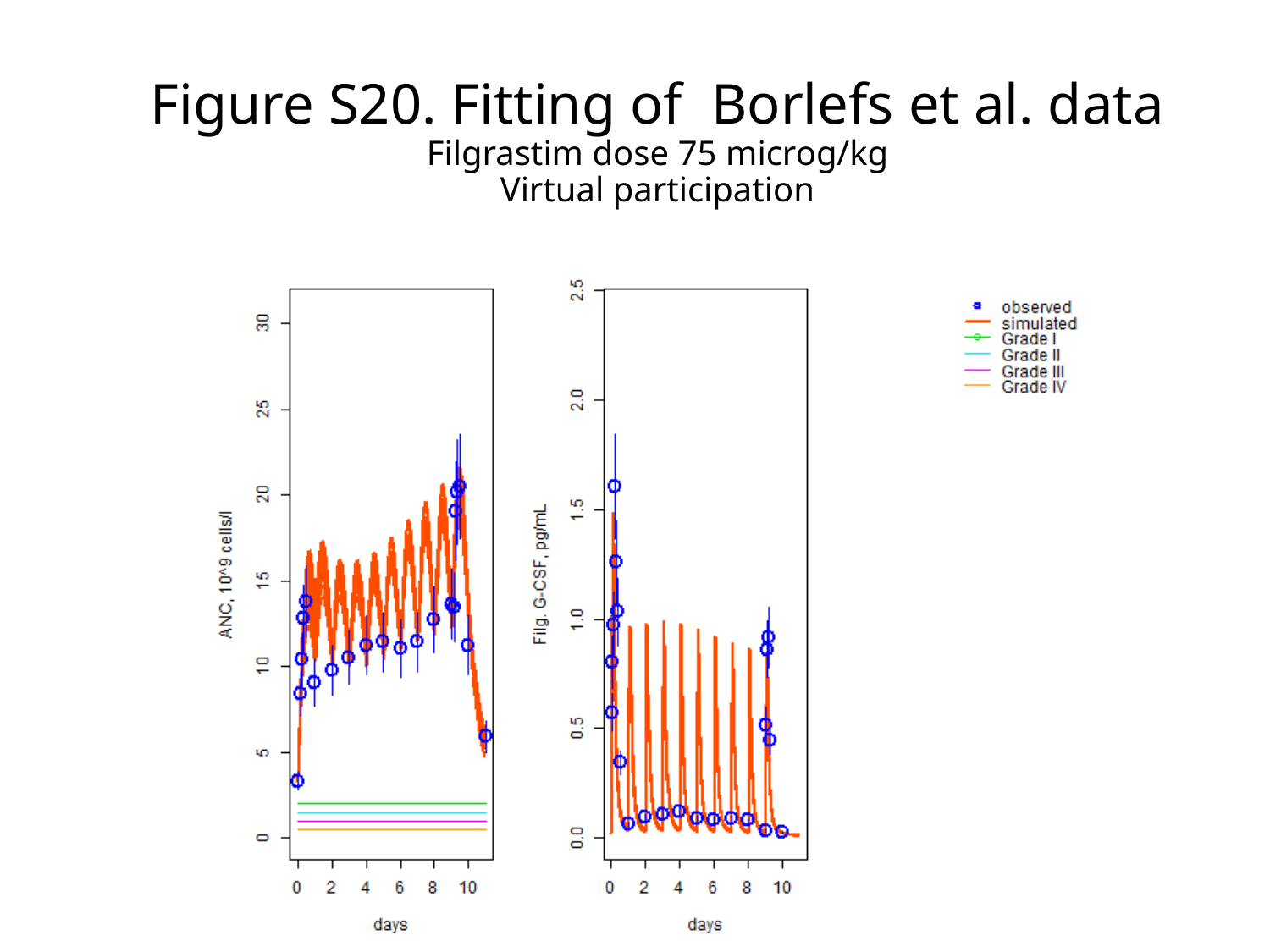

# Figure S20. Fitting of Borlefs et al. data Filgrastim dose 75 microg/kg Virtual participation

## Slide 21
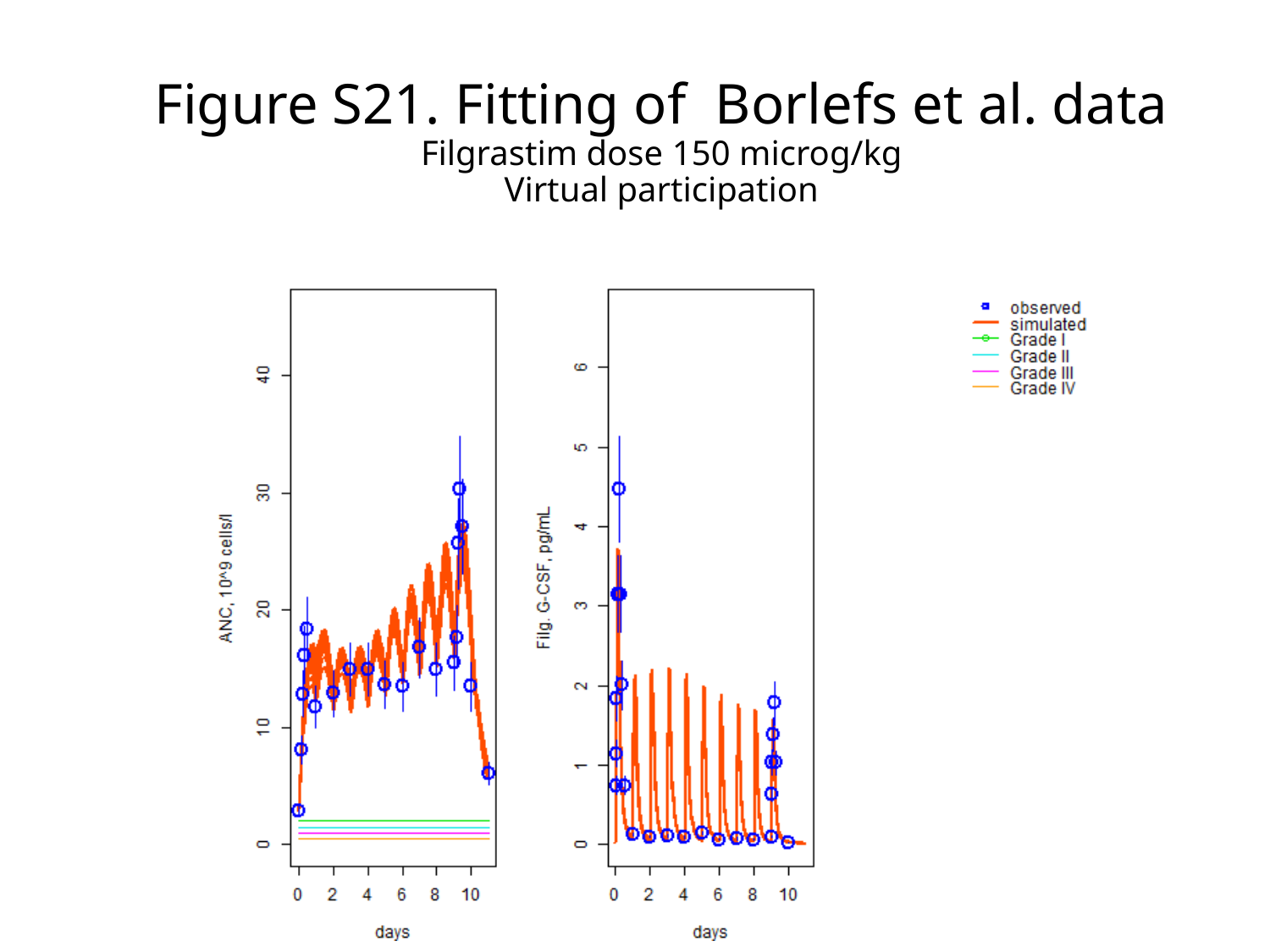

# Figure S21. Fitting of Borlefs et al. data Filgrastim dose 150 microg/kg Virtual participation

## Slide 22
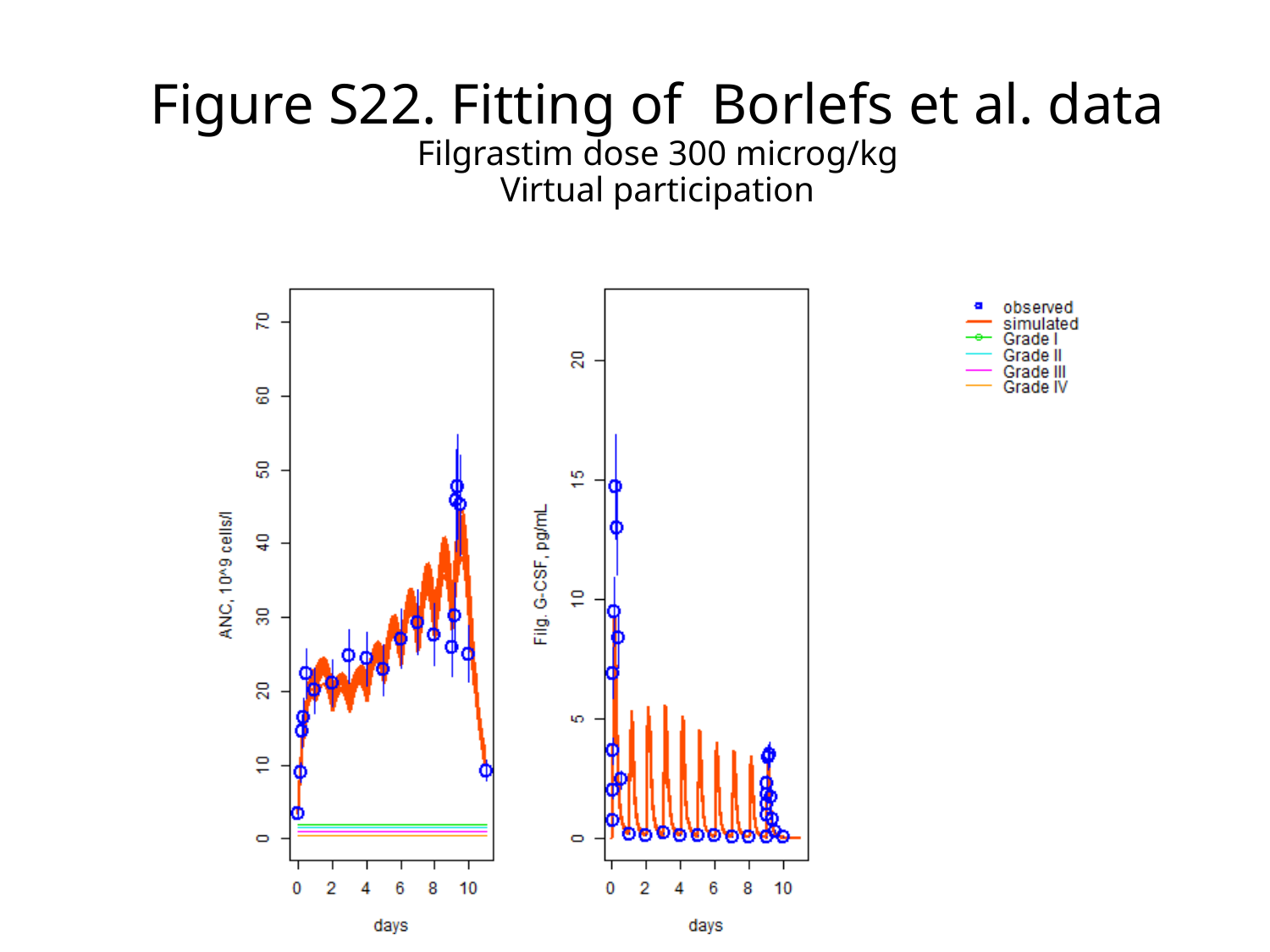

# Figure S22. Fitting of Borlefs et al. data Filgrastim dose 300 microg/kg Virtual participation

## Slide 23
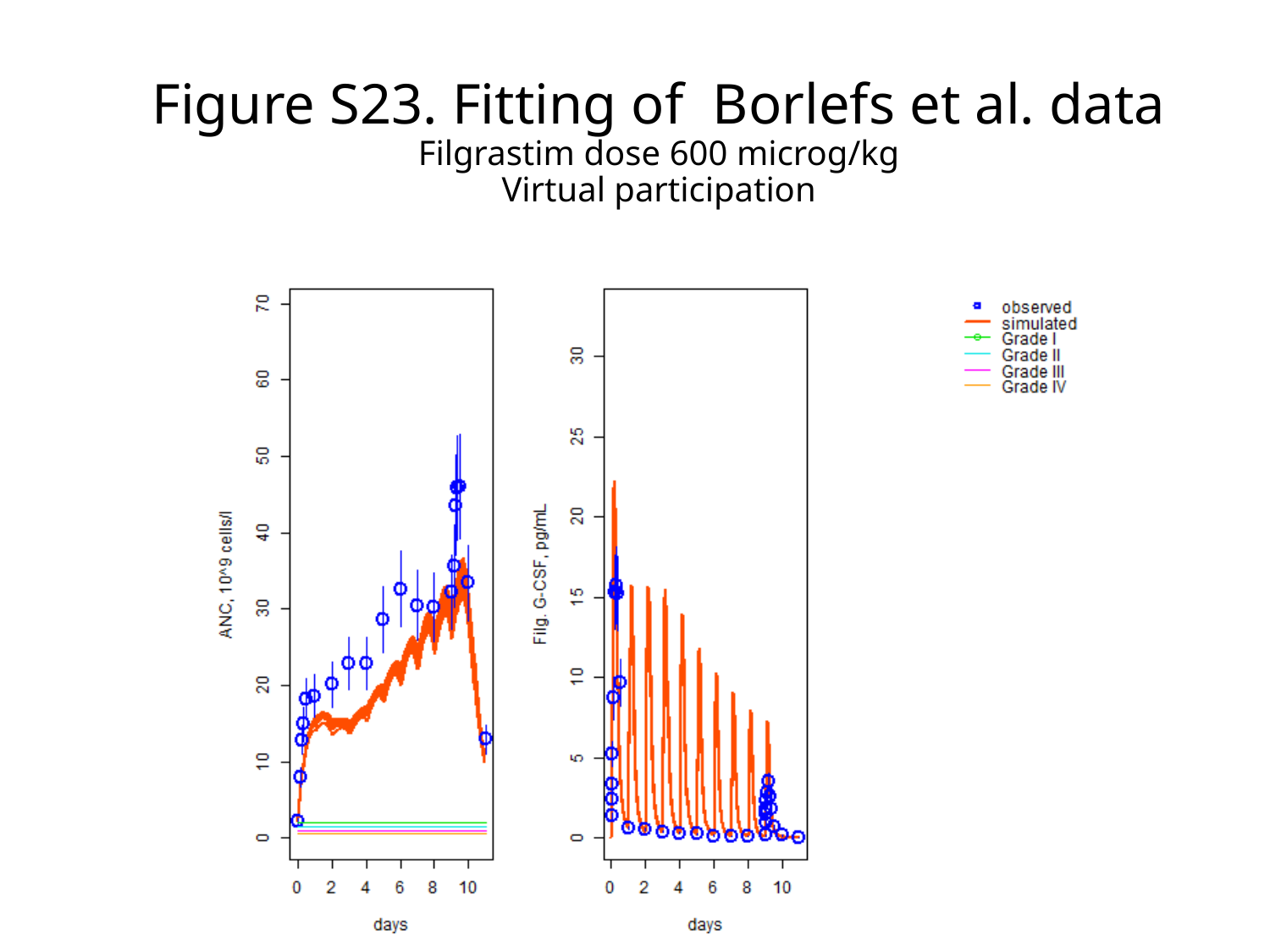

# Figure S23. Fitting of Borlefs et al. data Filgrastim dose 600 microg/kg Virtual participation
