# Supplementary material for: Individual hematotoxicity prediction of further chemotherapy cycles by dynamic mathematical models in patients with gastrointestinal tumors
Source: J Cancer Res Clin Oncol. 2023 Feb 28;149(10):6989–98. doi: 10.1007/s00432-023-04601-9 (PMC10374676; doi:10.1007/s00432-023-04601-9)
Supplement: Supplementary file 2 — Supplementary file2 (PPTX 628 KB) [file 432_2023_4601_MOESM2_ESM.pptx]

## Slide 1
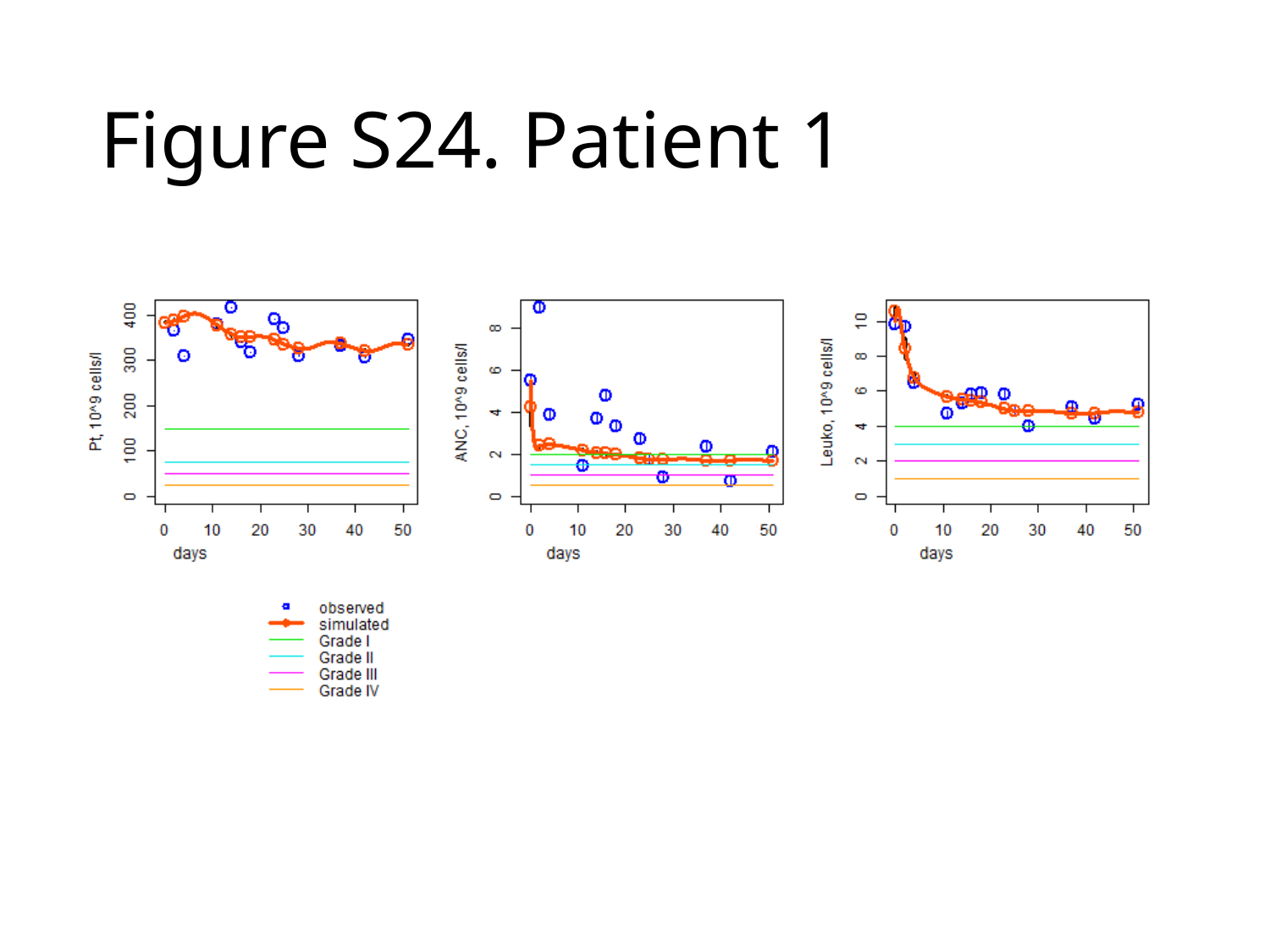

# Figure S24. Patient 1

## Slide 2
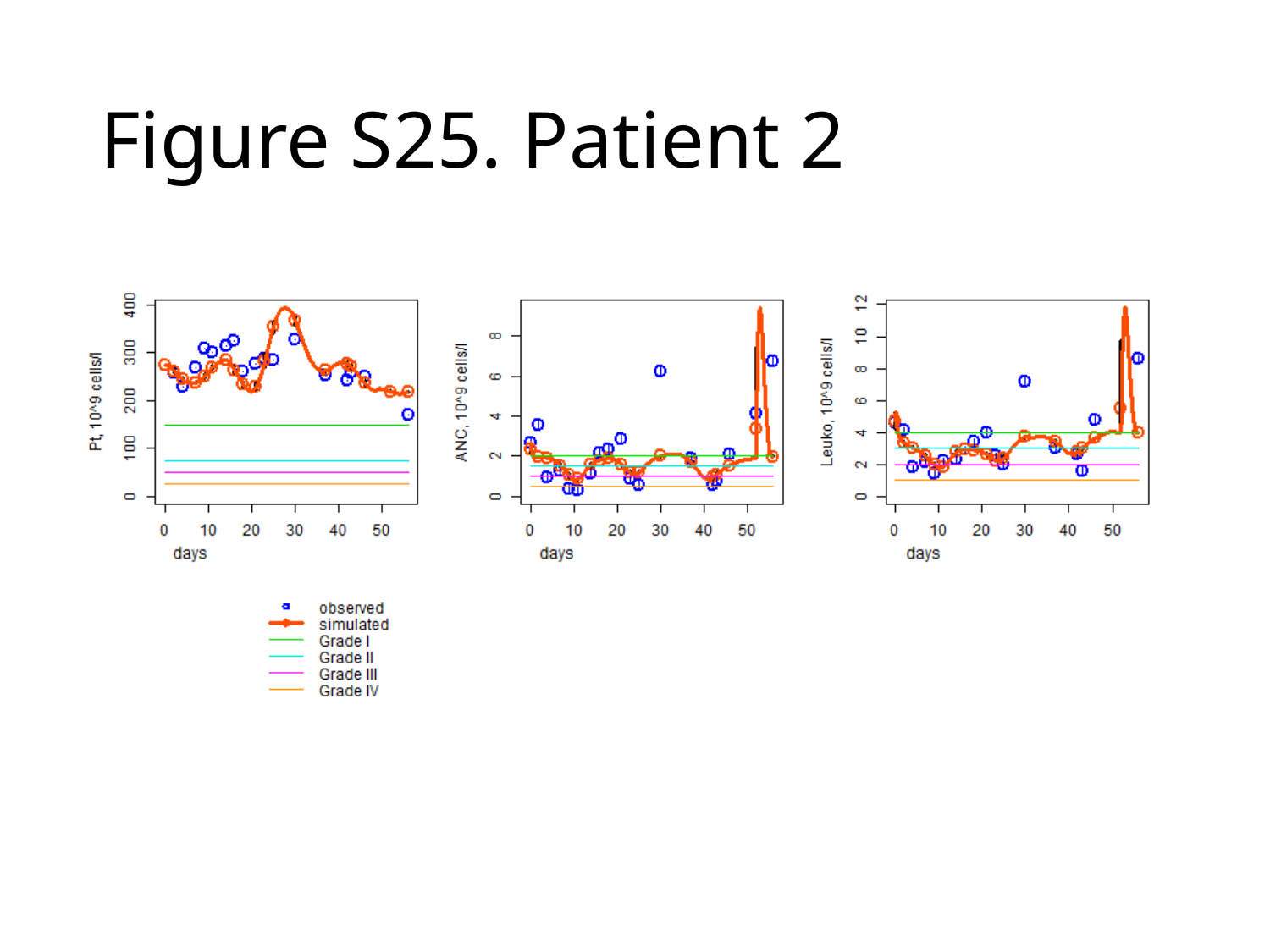

# Figure S25. Patient 2

## Slide 3
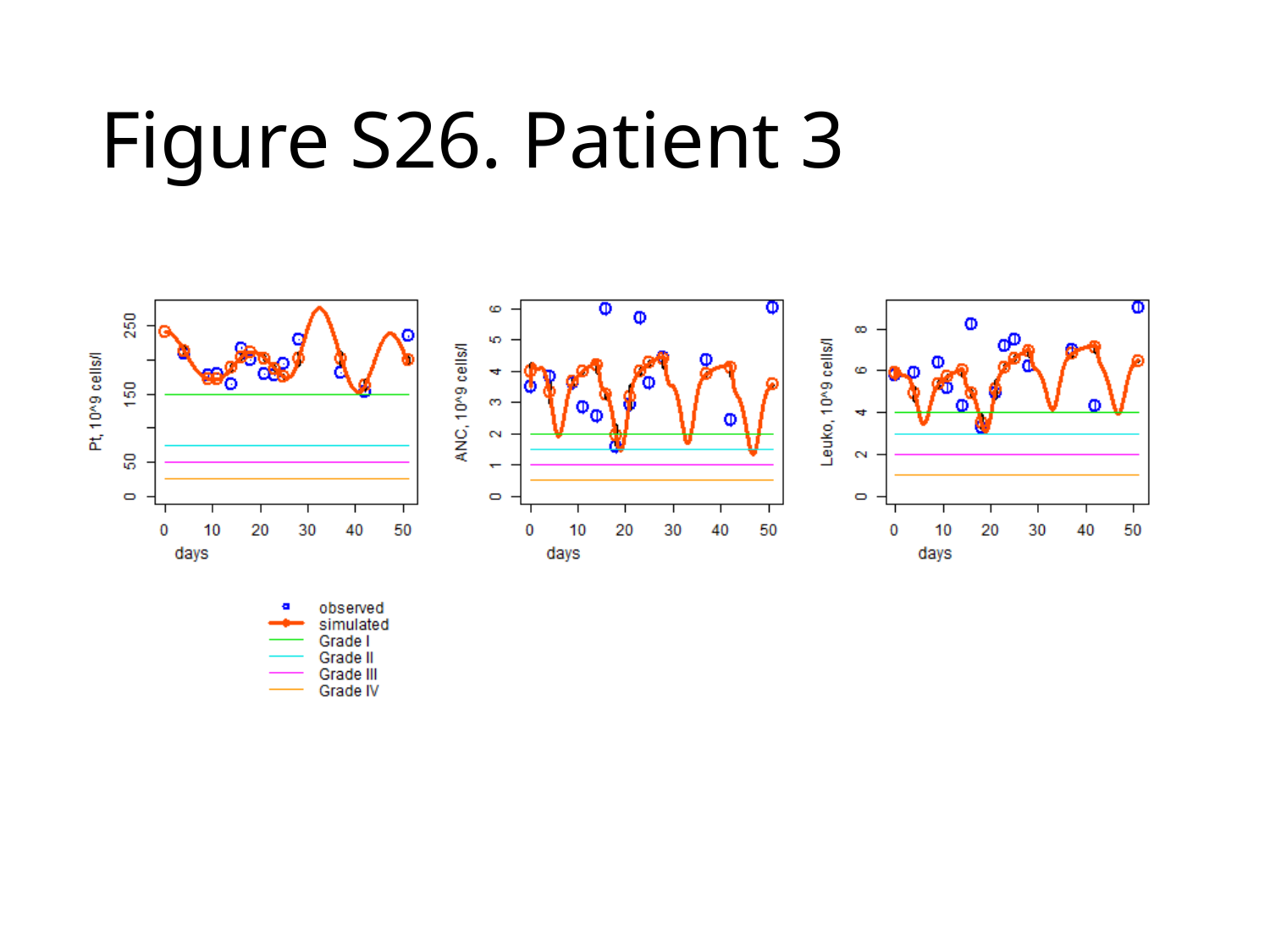

# Figure S26. Patient 3

## Slide 4
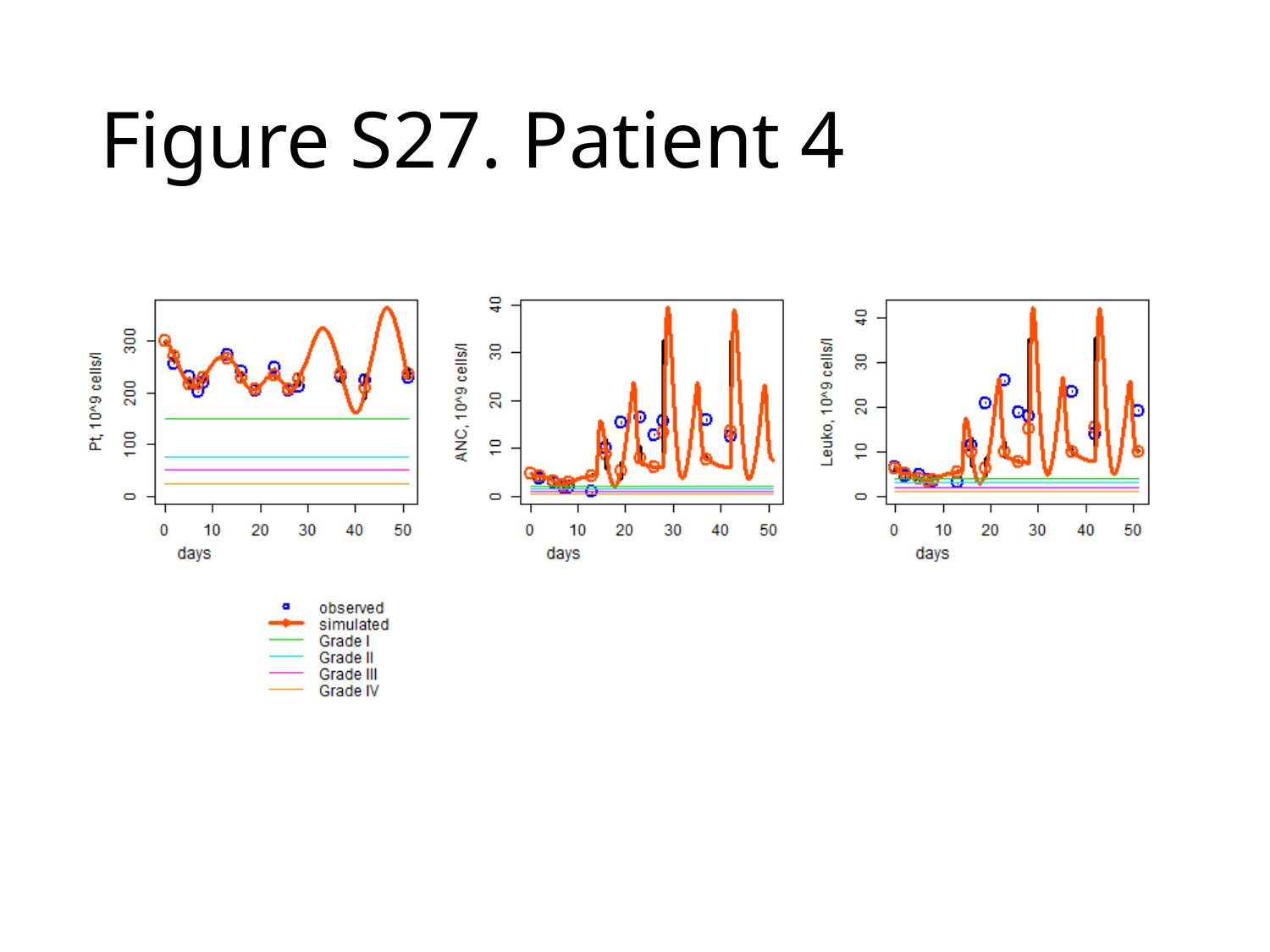

# Figure S27. Patient 4

## Slide 5
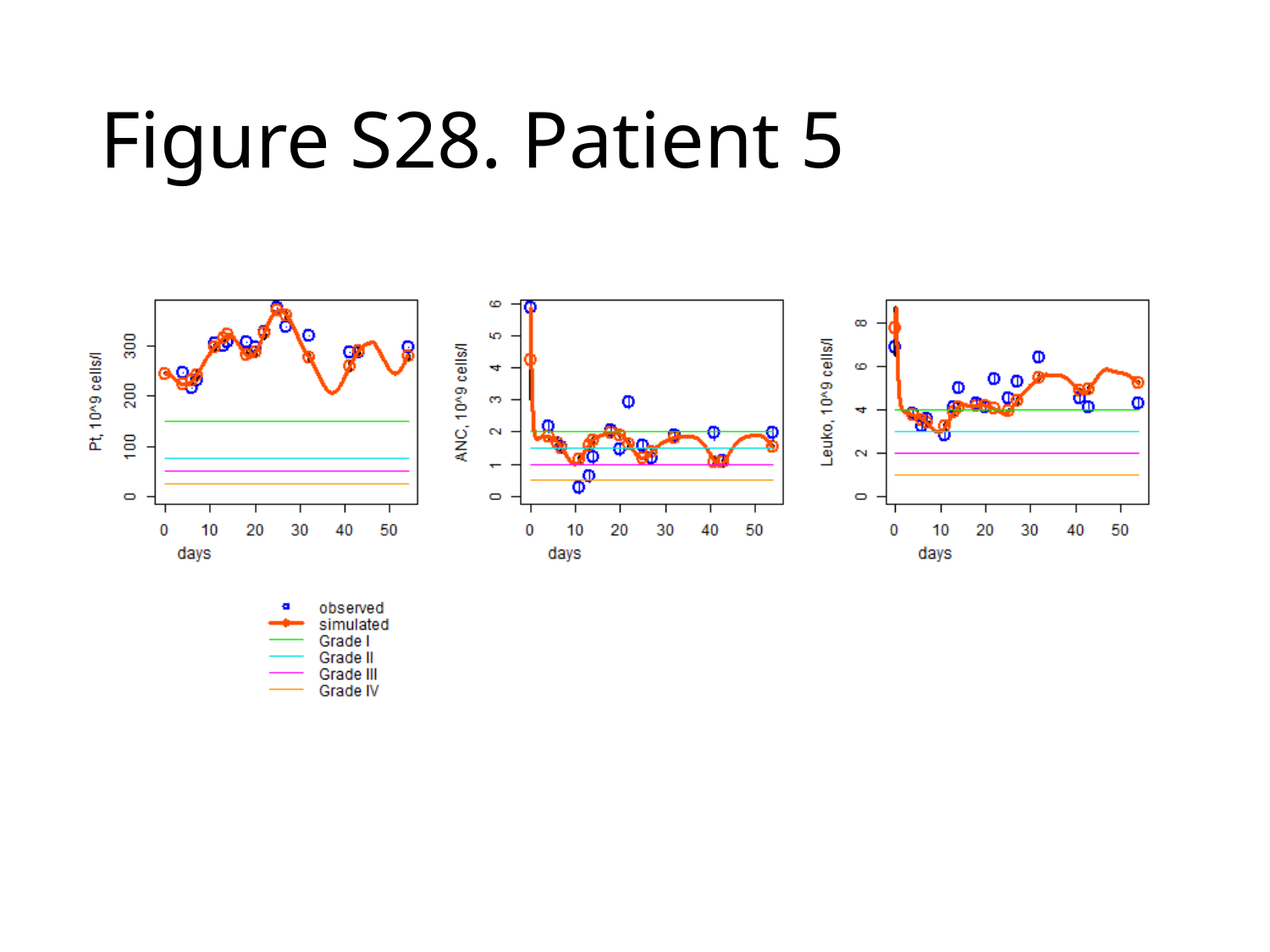

# Figure S28. Patient 5

## Slide 6
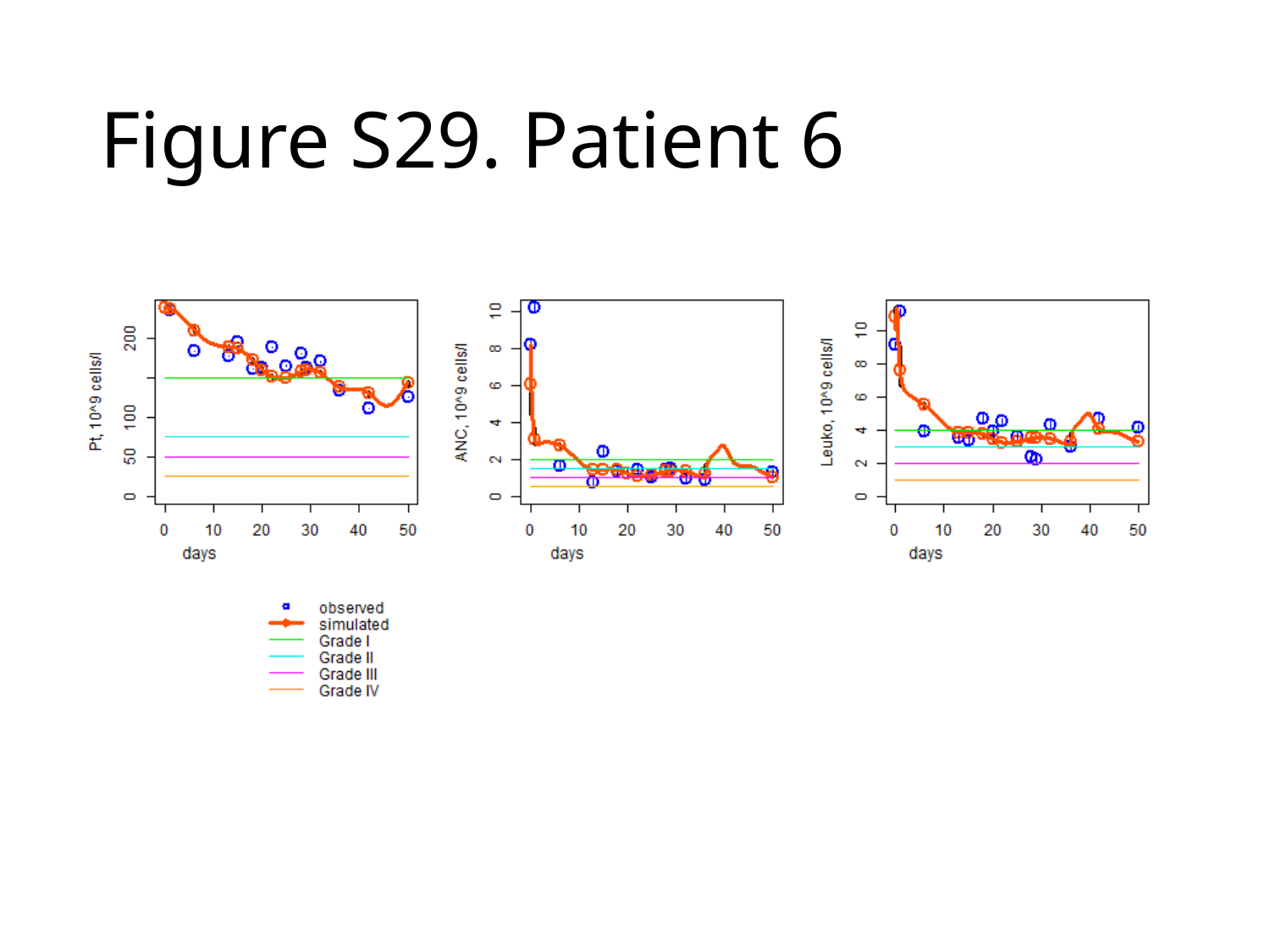

# Figure S29. Patient 6

## Slide 7
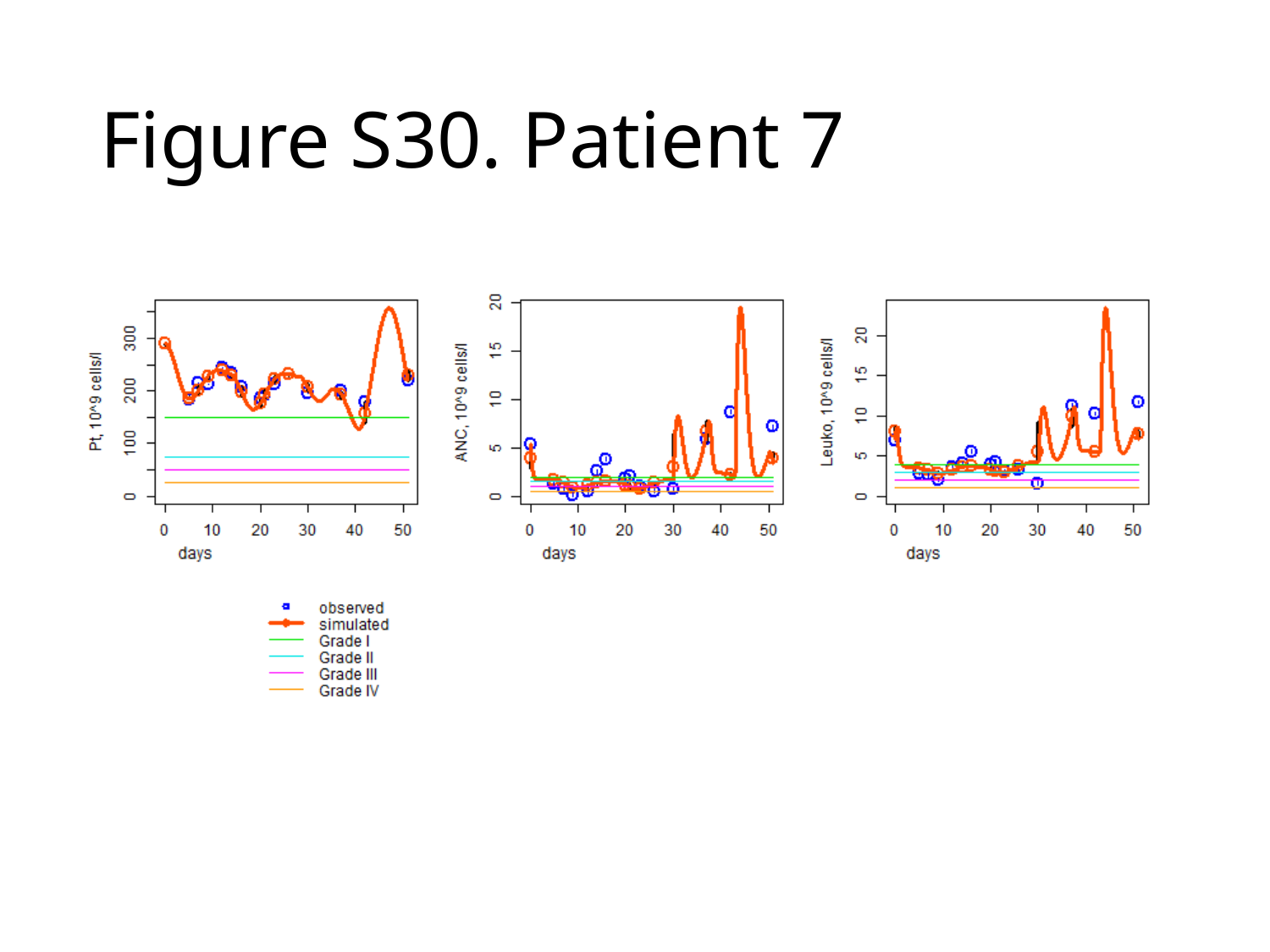

# Figure S30. Patient 7

## Slide 8
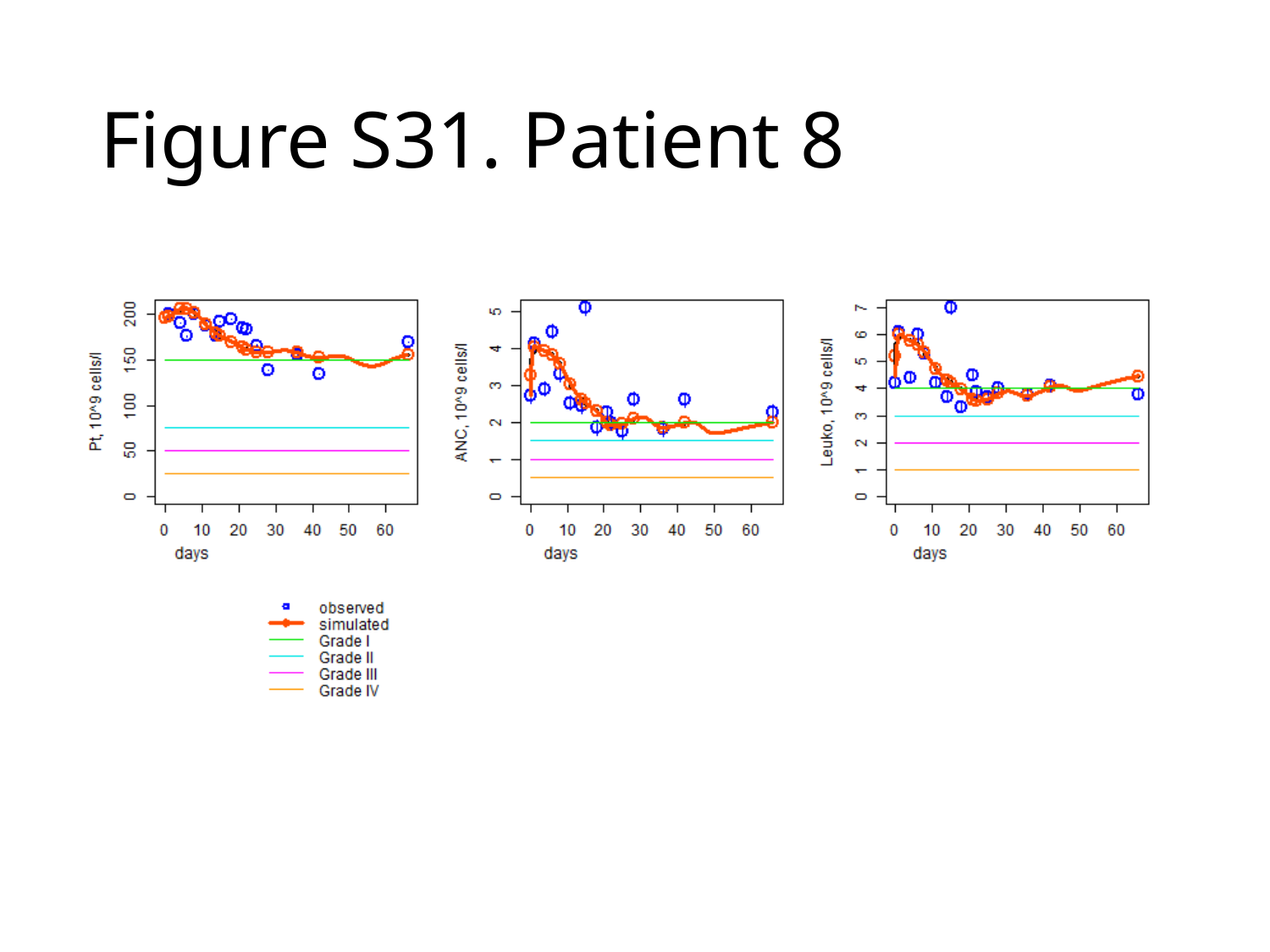

# Figure S31. Patient 8

## Slide 9
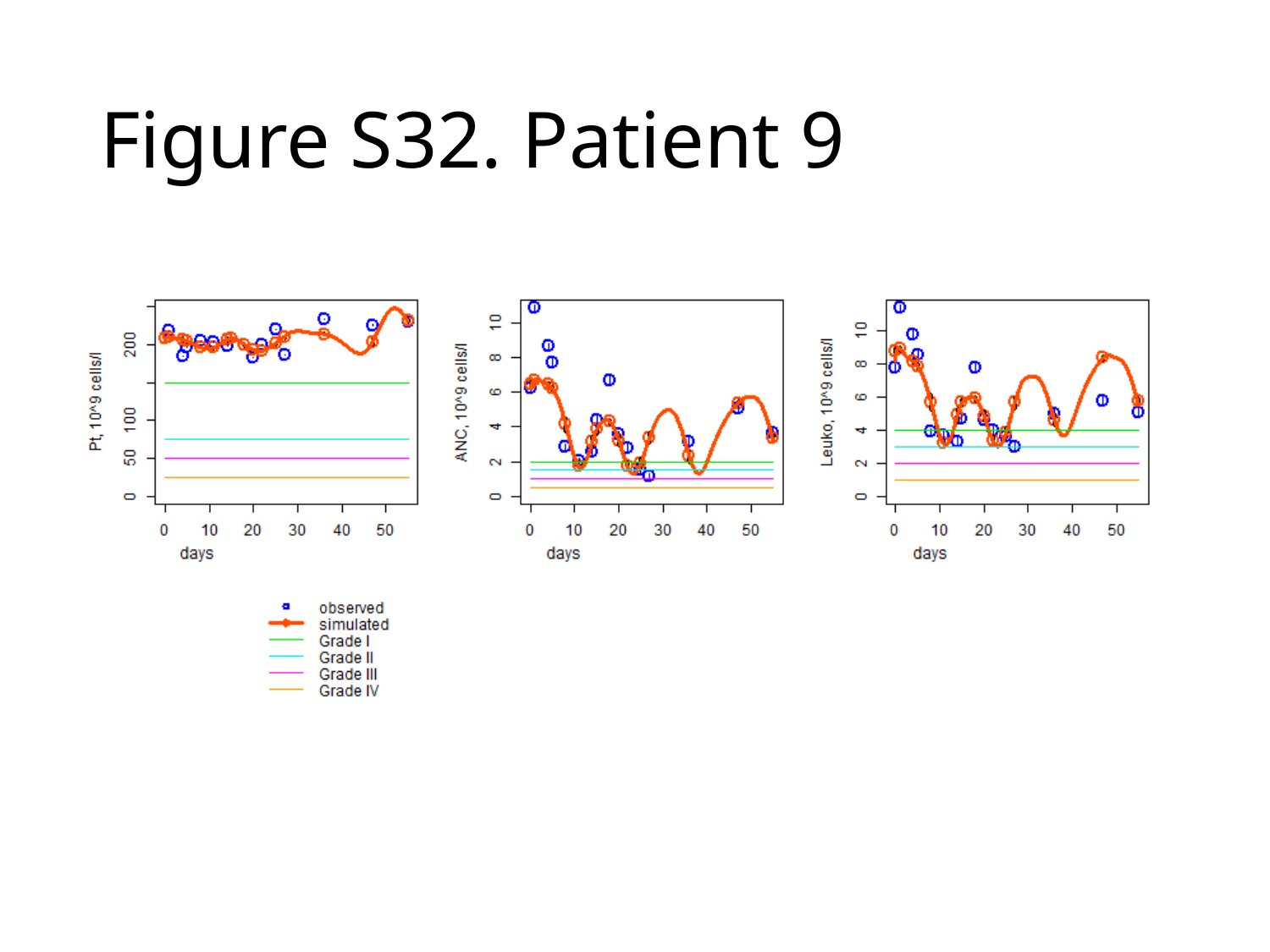

# Figure S32. Patient 9

## Slide 10
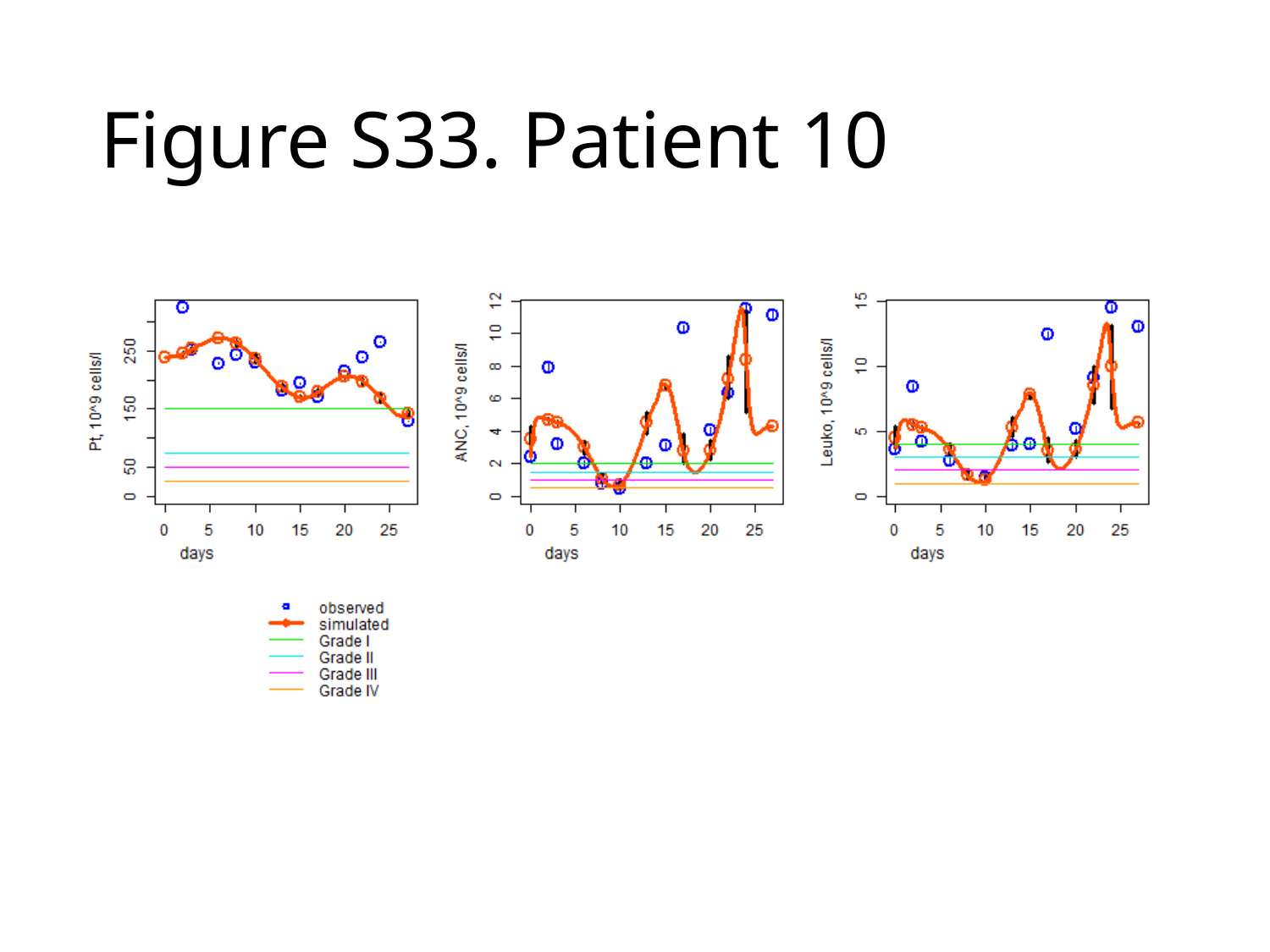

# Figure S33. Patient 10

## Slide 11
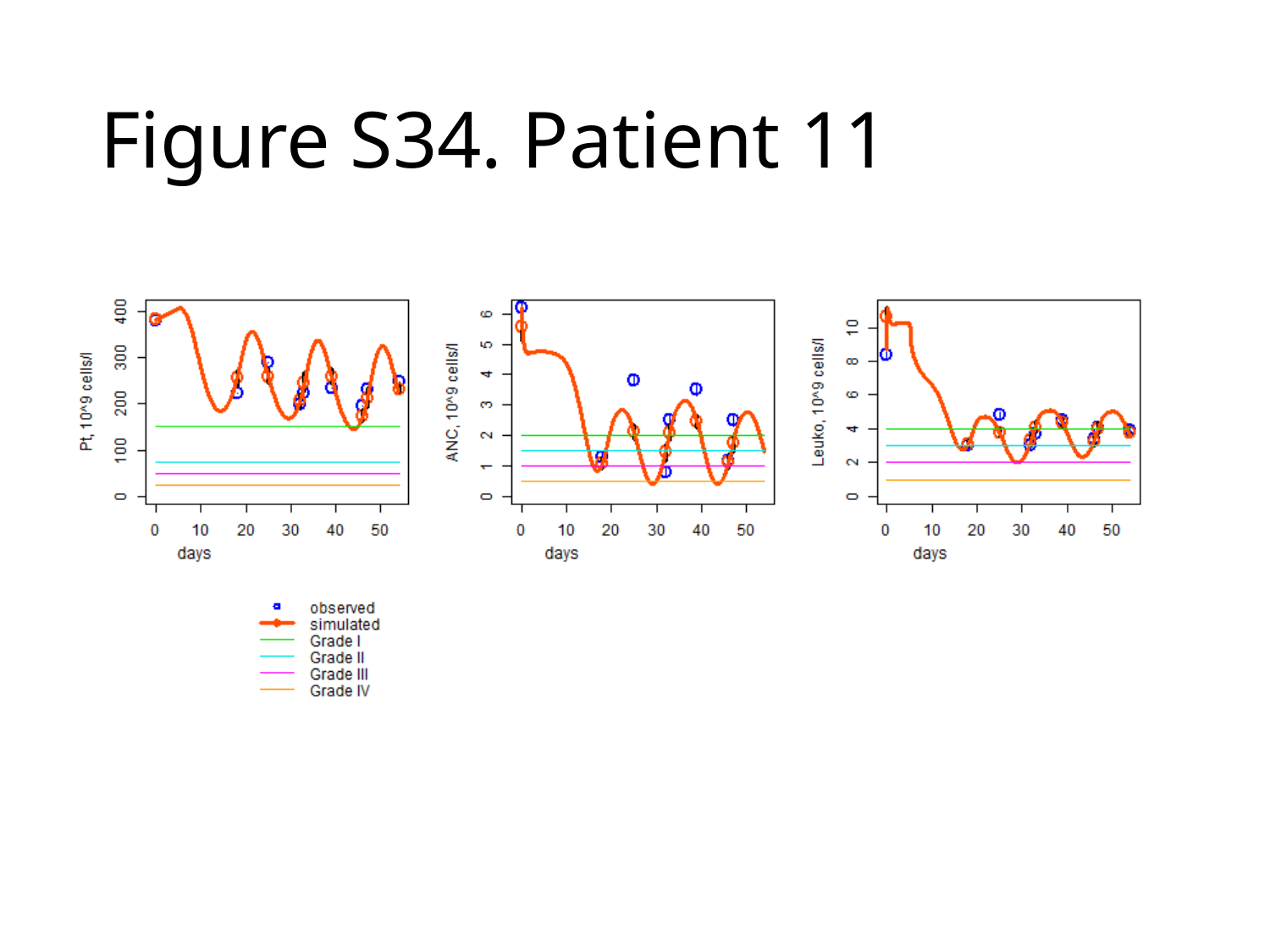

# Figure S34. Patient 11

## Slide 12
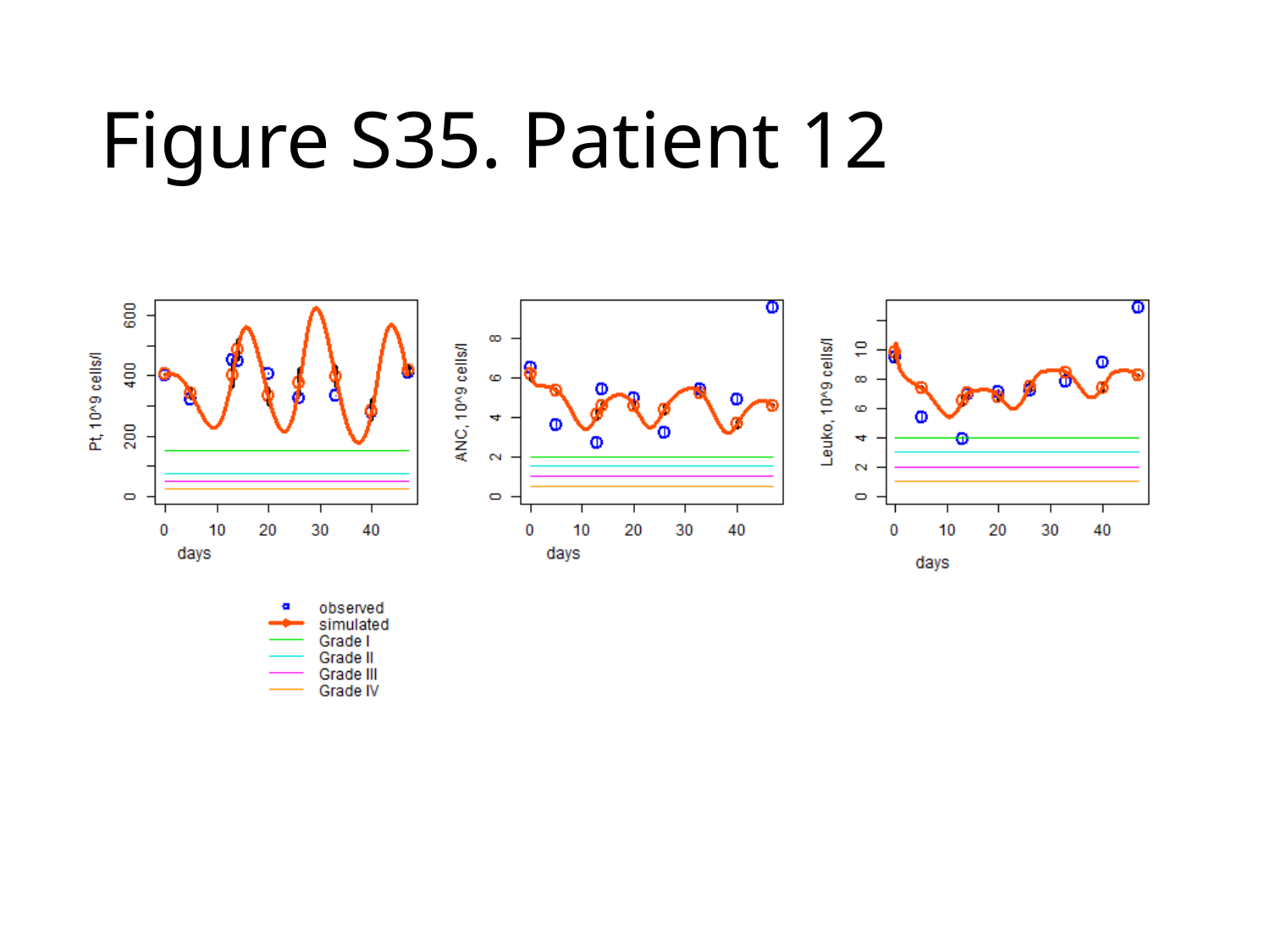

# Figure S35. Patient 12

## Slide 13
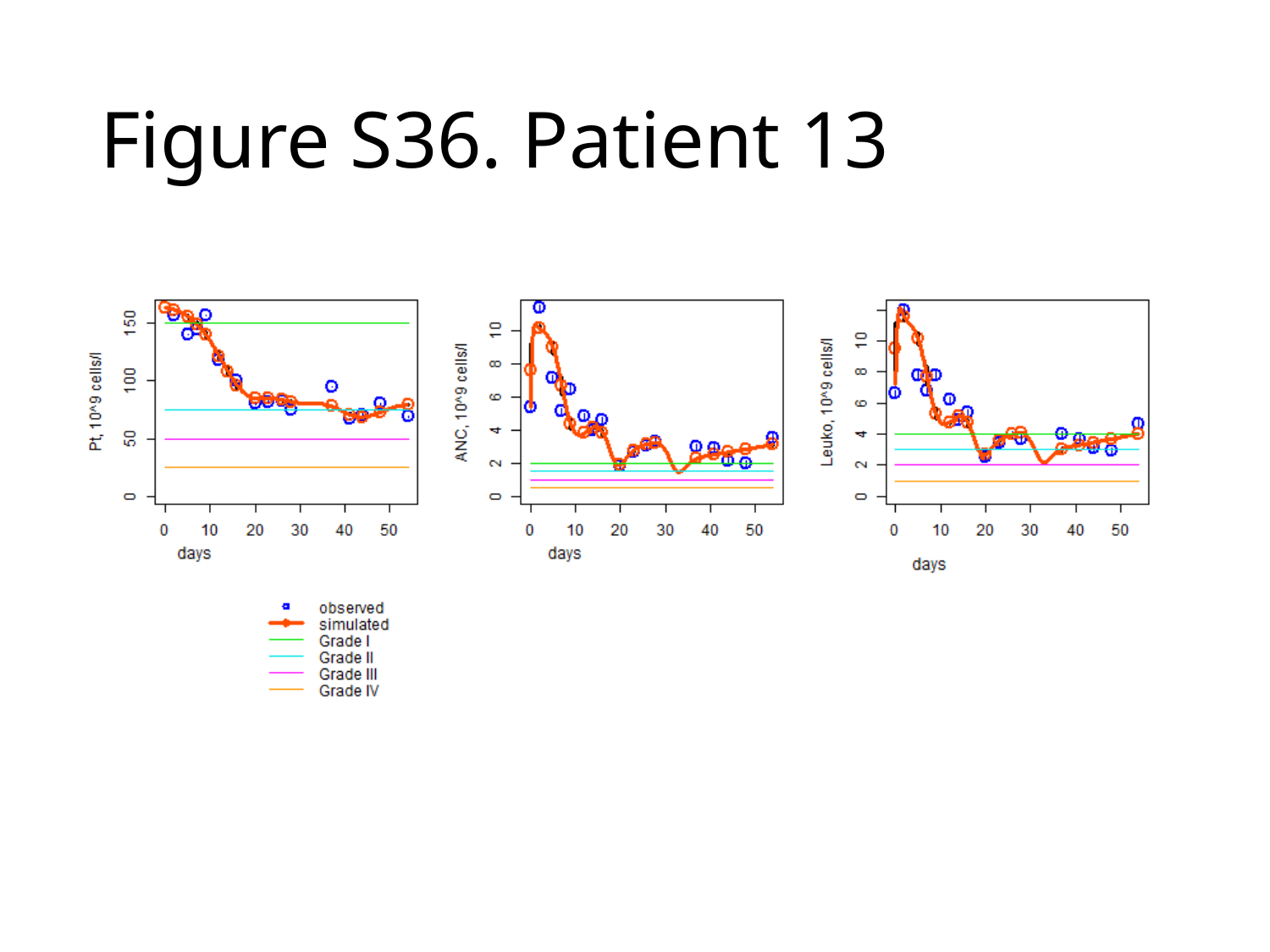

# Figure S36. Patient 13

## Slide 14
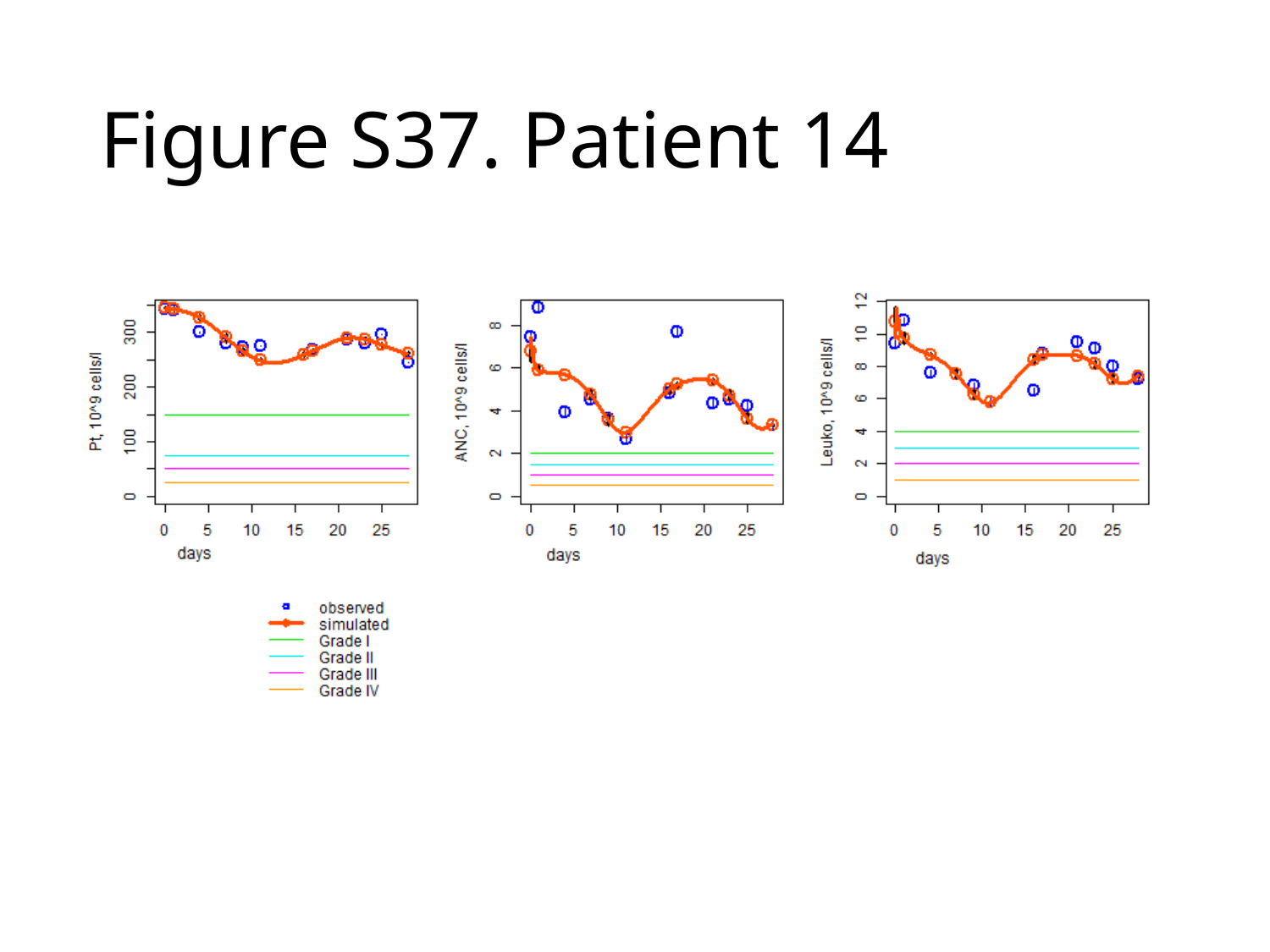

# Figure S37. Patient 14

## Slide 15
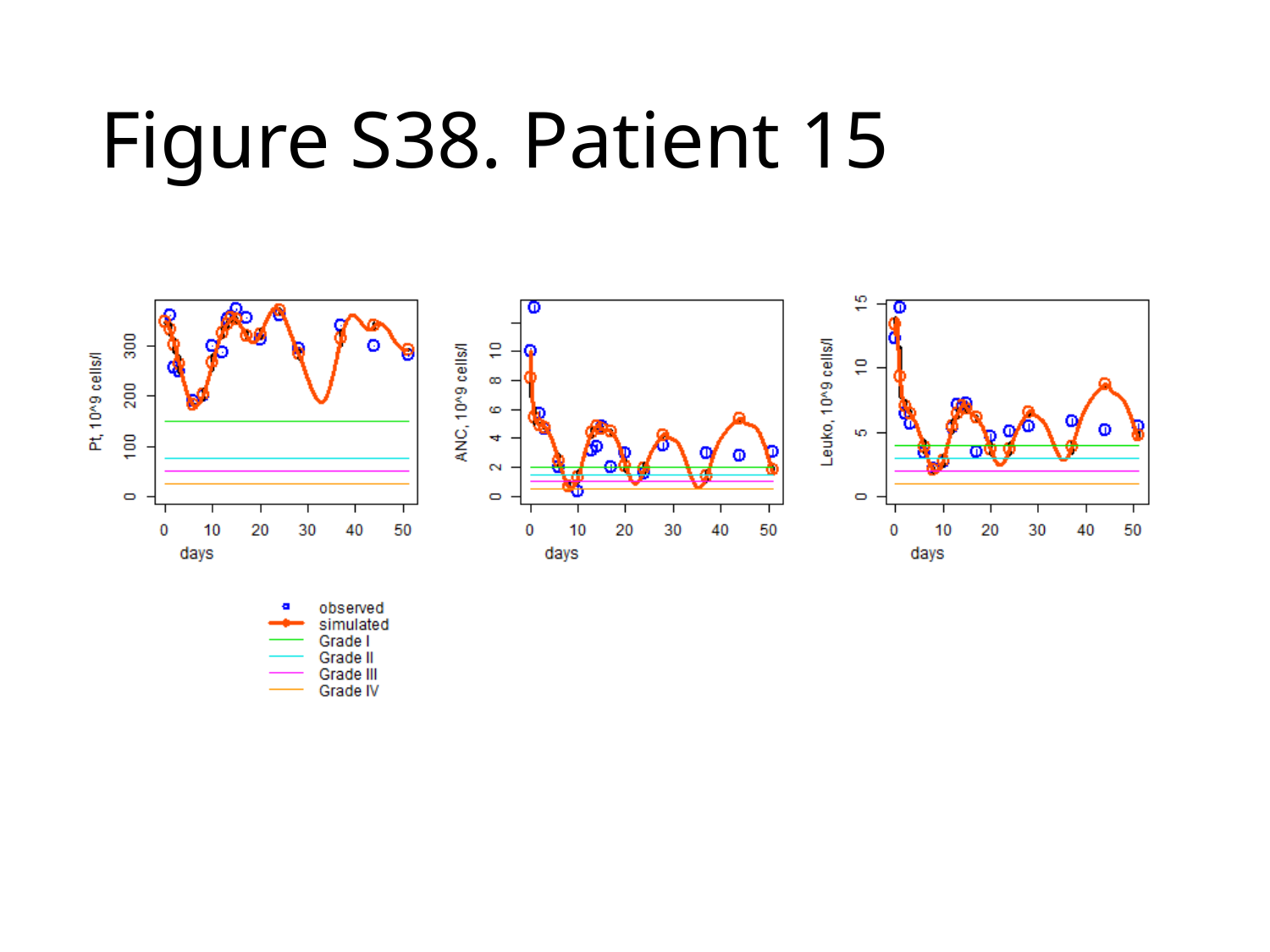

# Figure S38. Patient 15

## Slide 16
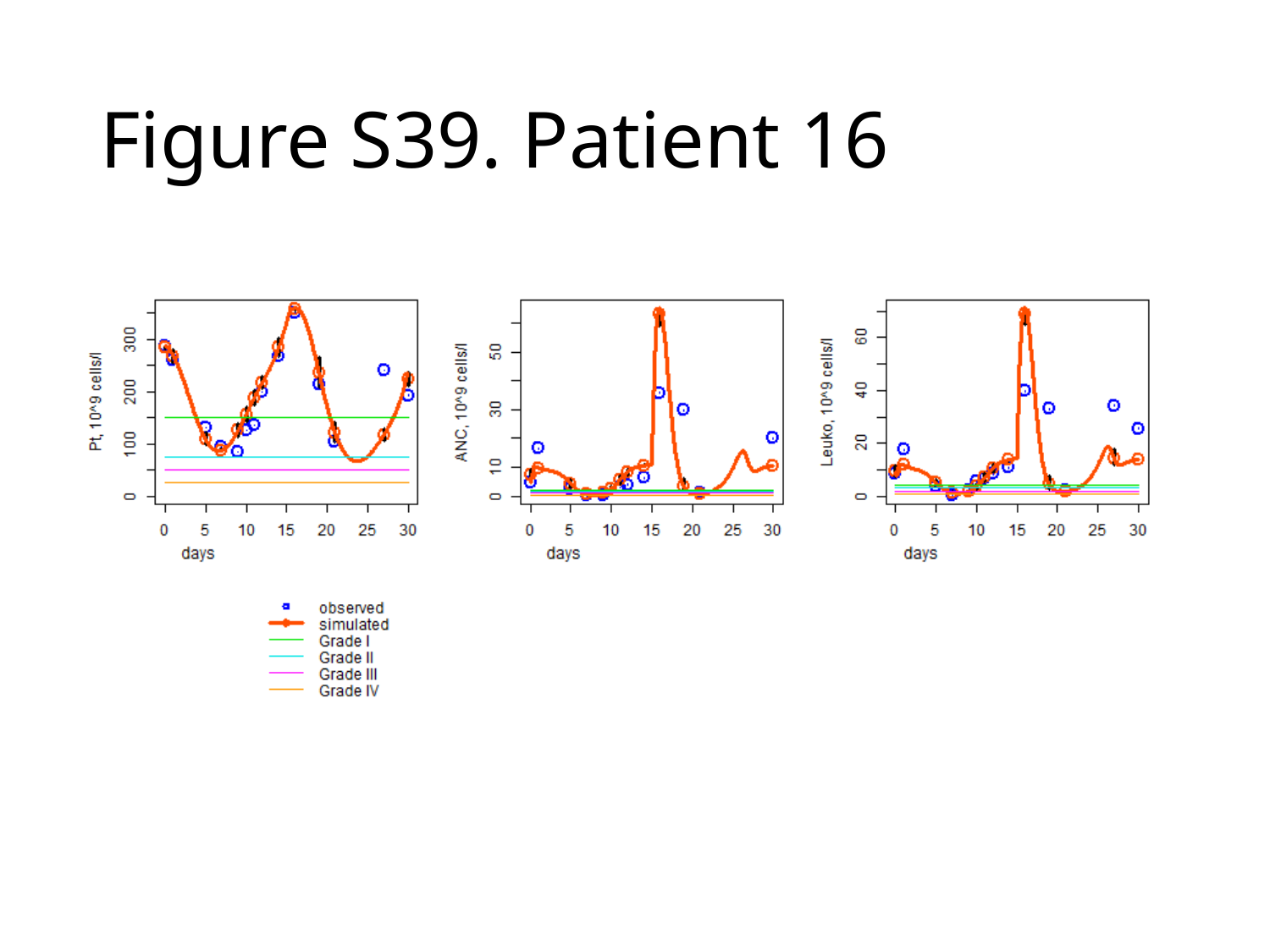

# Figure S39. Patient 16

## Slide 17
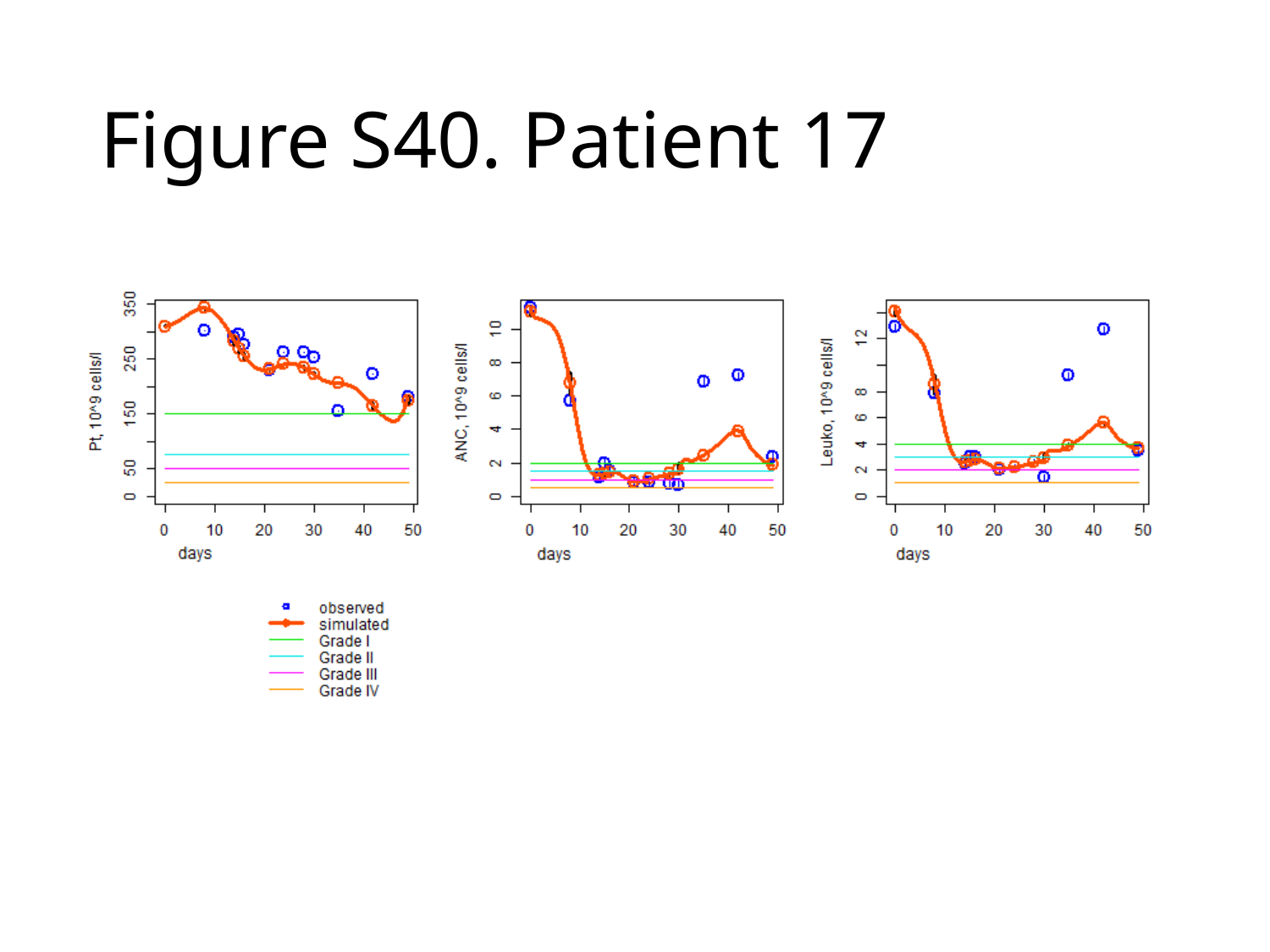

# Figure S40. Patient 17

## Slide 18
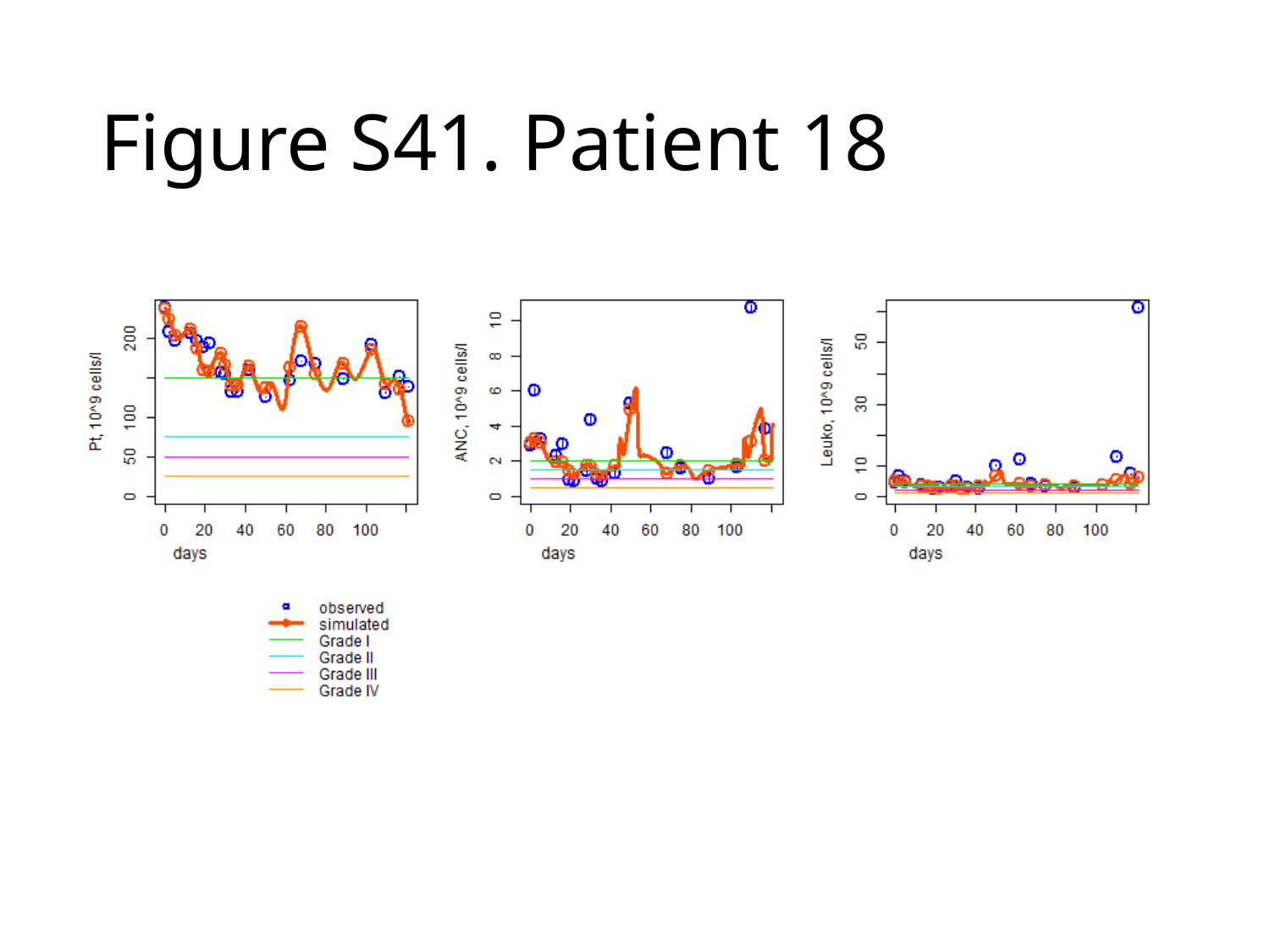

# Figure S41. Patient 18

## Slide 19
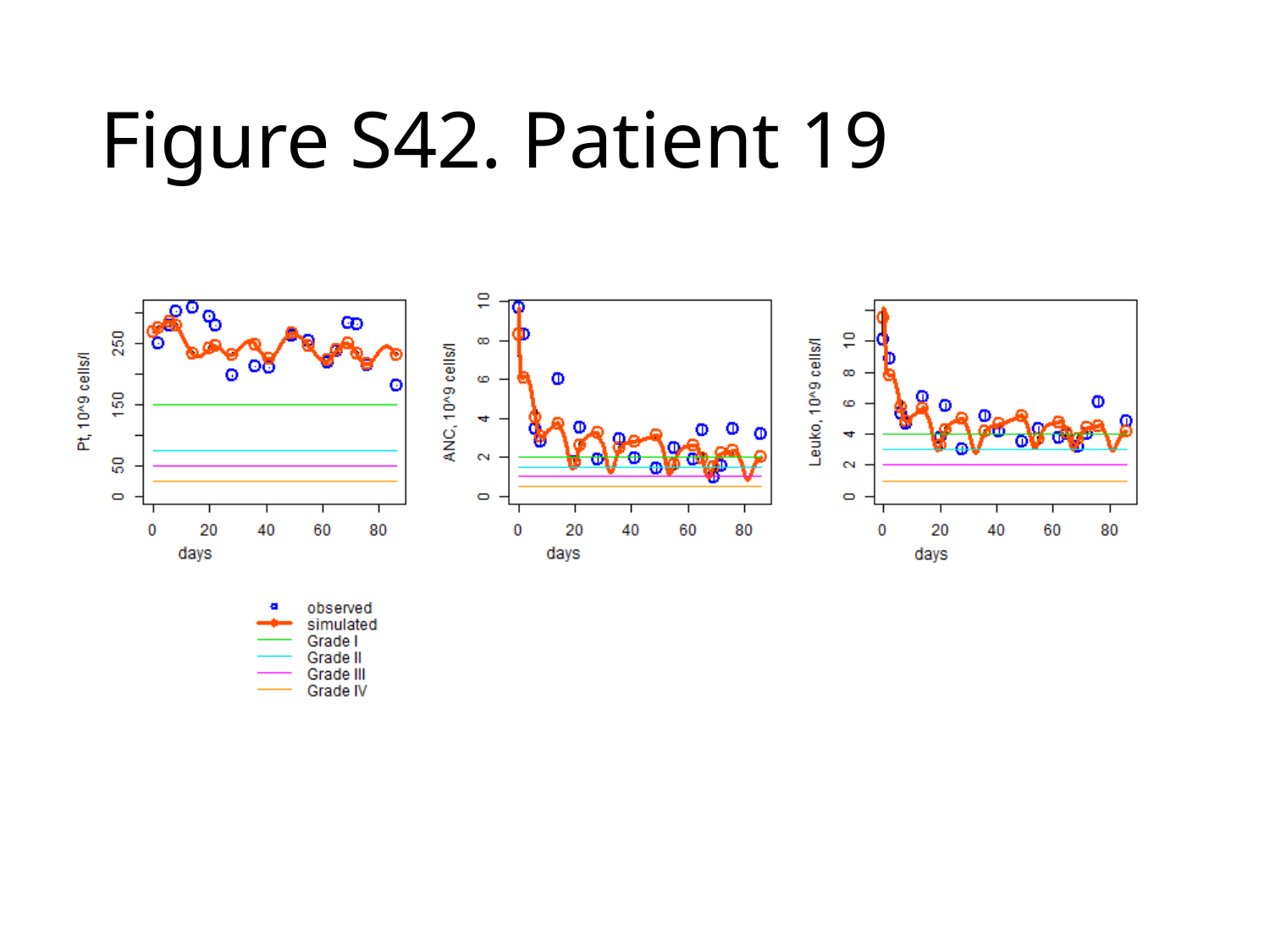

# Figure S42. Patient 19

## Slide 20
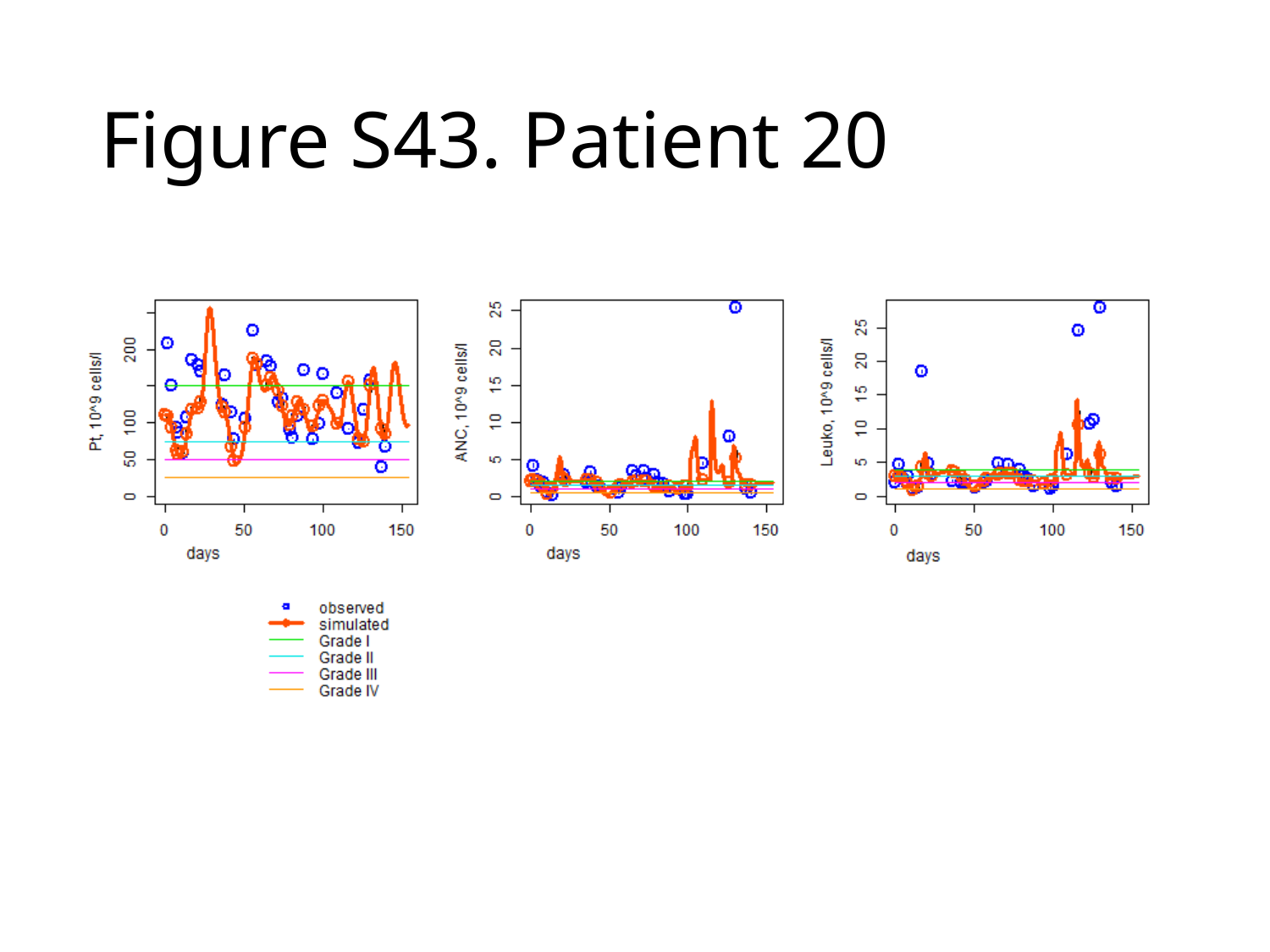

# Figure S43. Patient 20

## Slide 21
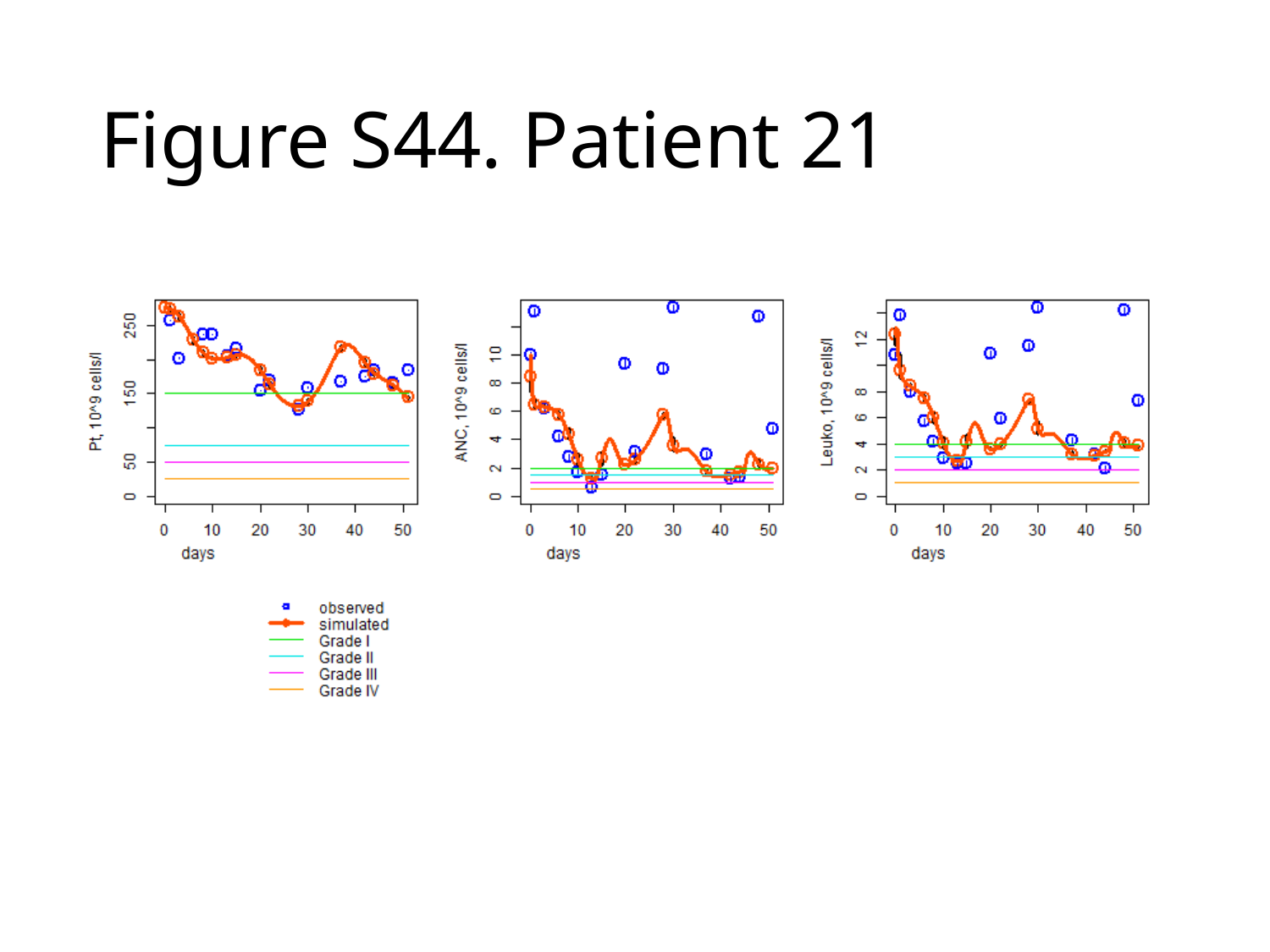

# Figure S44. Patient 21

## Slide 22
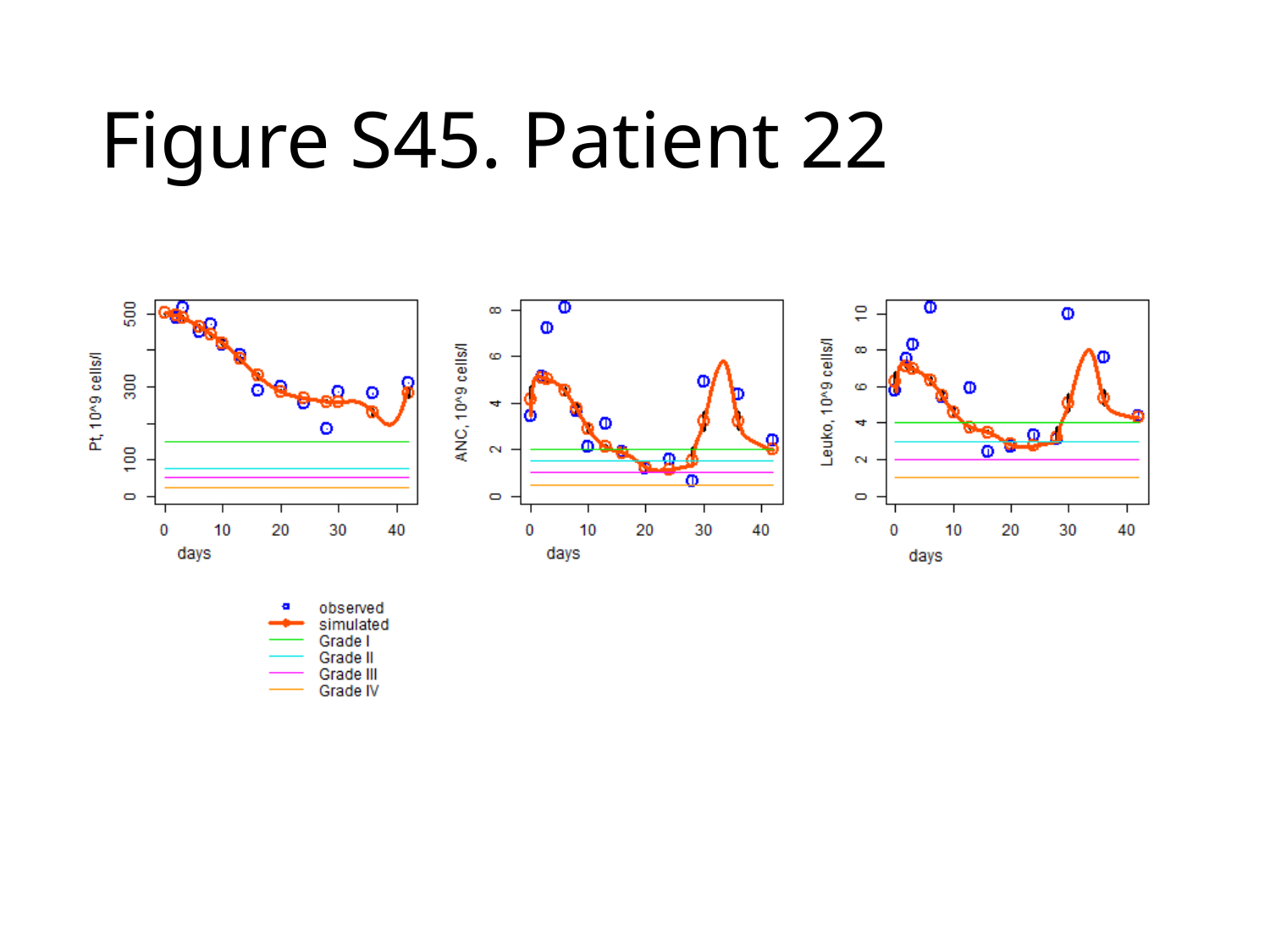

# Figure S45. Patient 22

## Slide 23
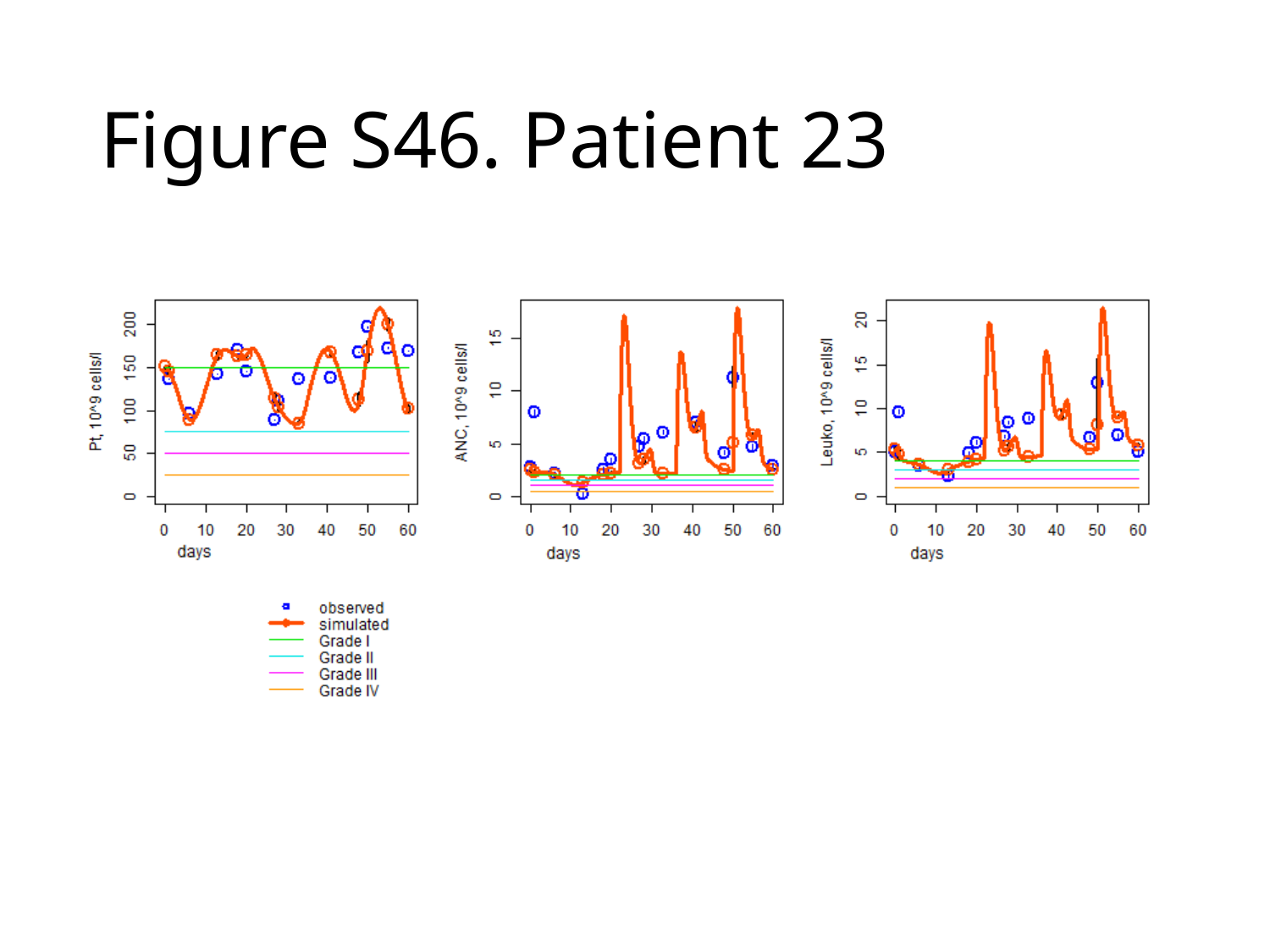

# Figure S46. Patient 23

## Slide 24
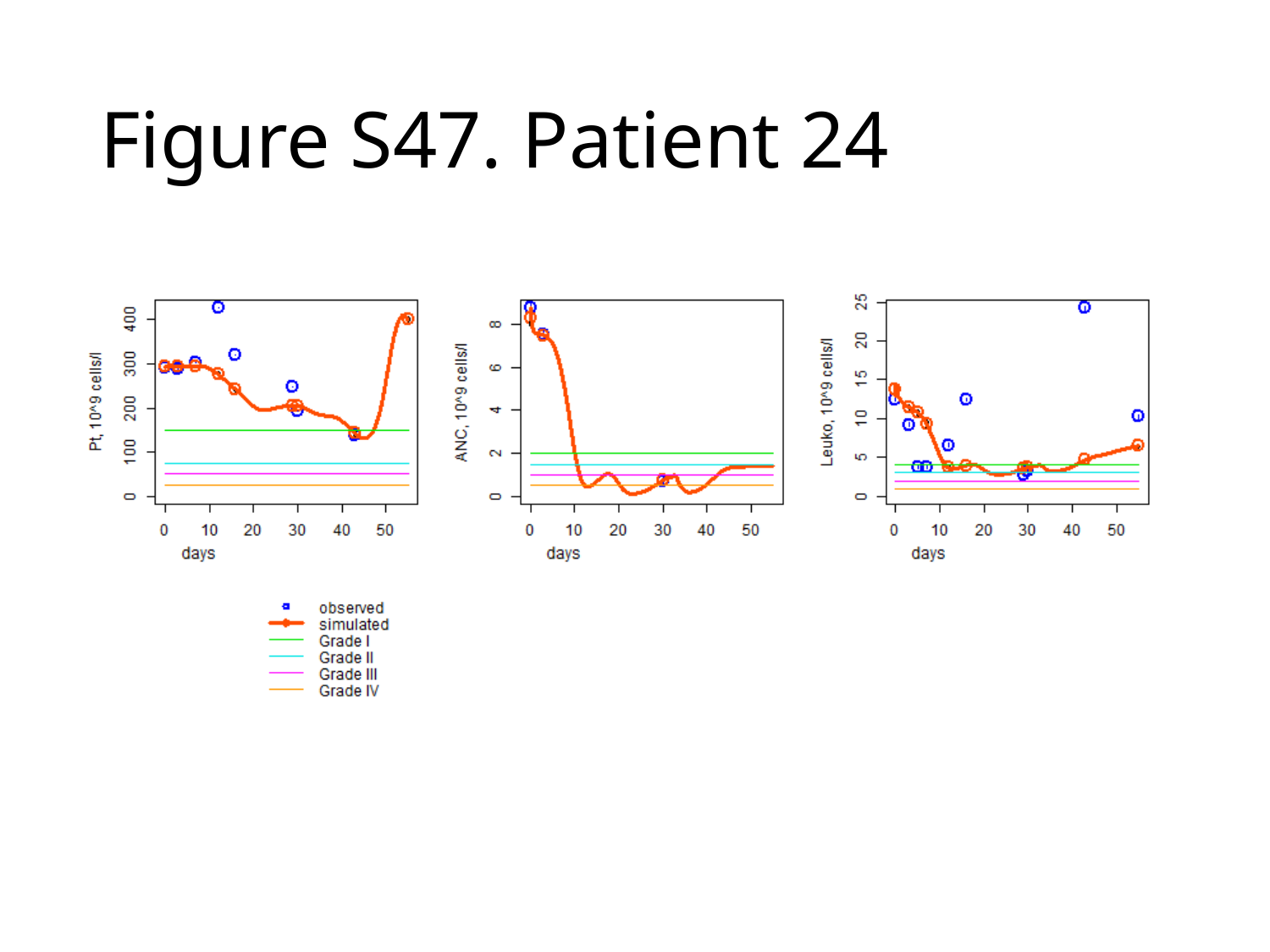

# Figure S47. Patient 24

## Slide 25
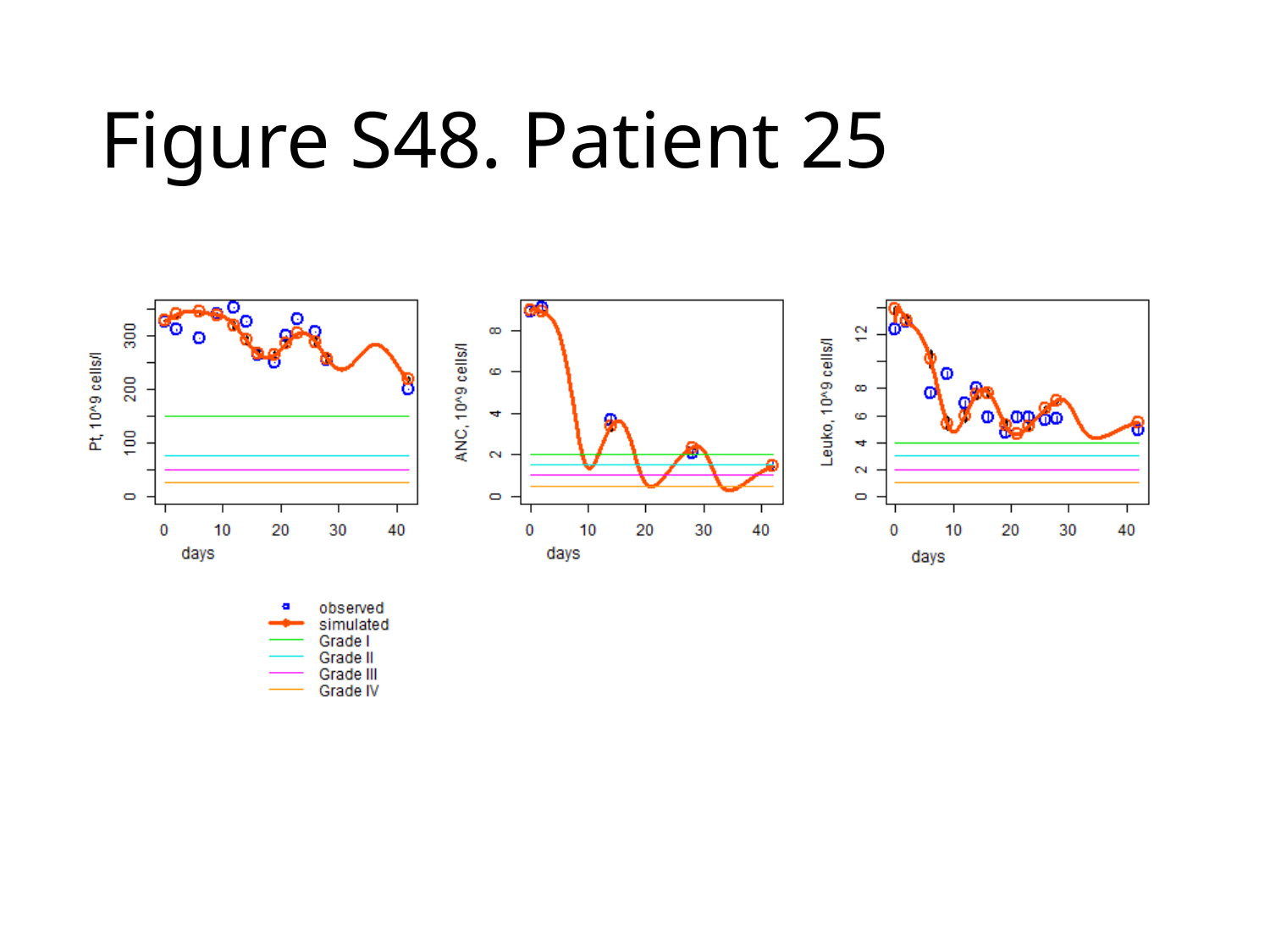

# Figure S48. Patient 25

## Slide 26
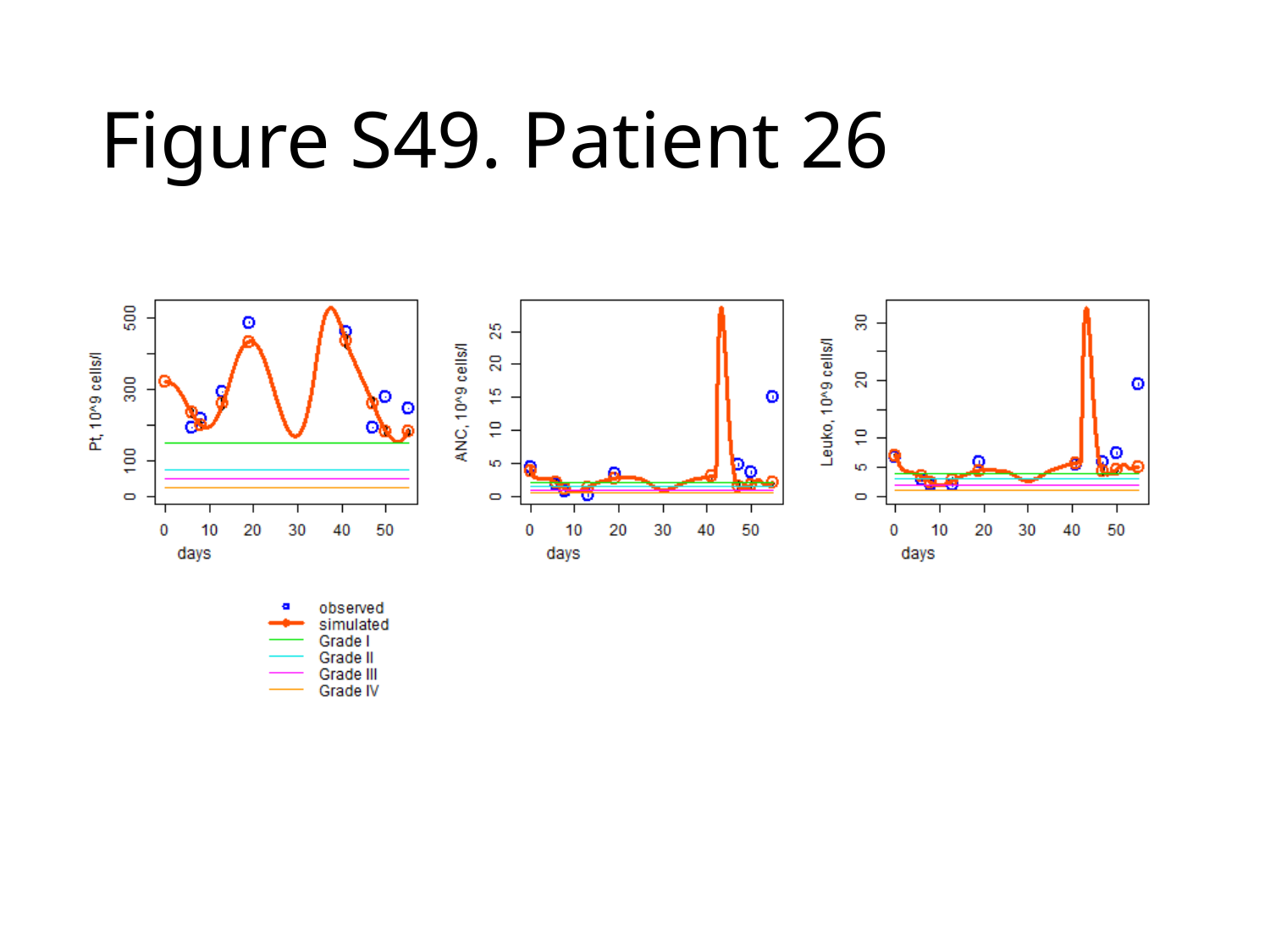

# Figure S49. Patient 26

## Slide 27
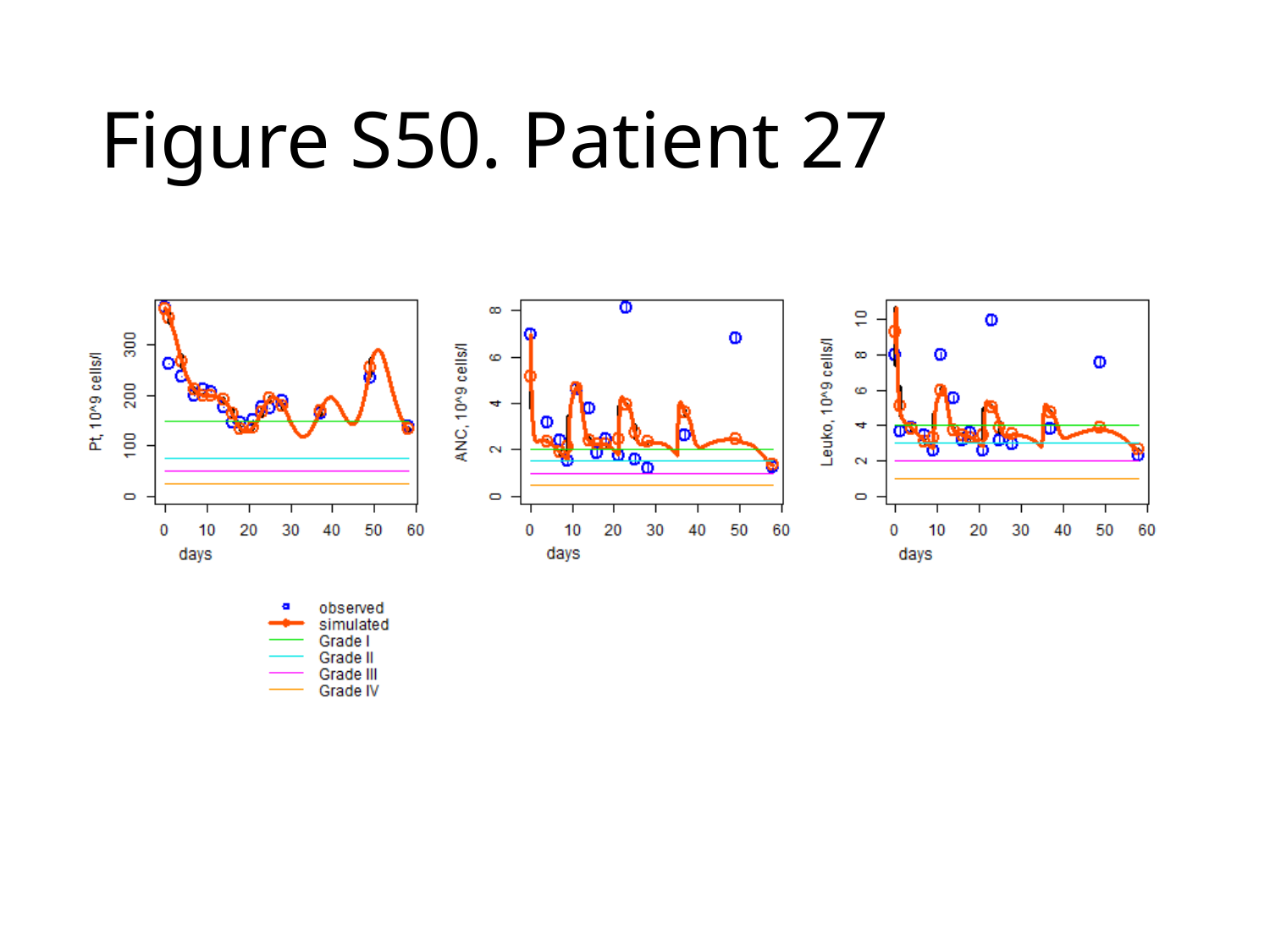

# Figure S50. Patient 27

## Slide 28
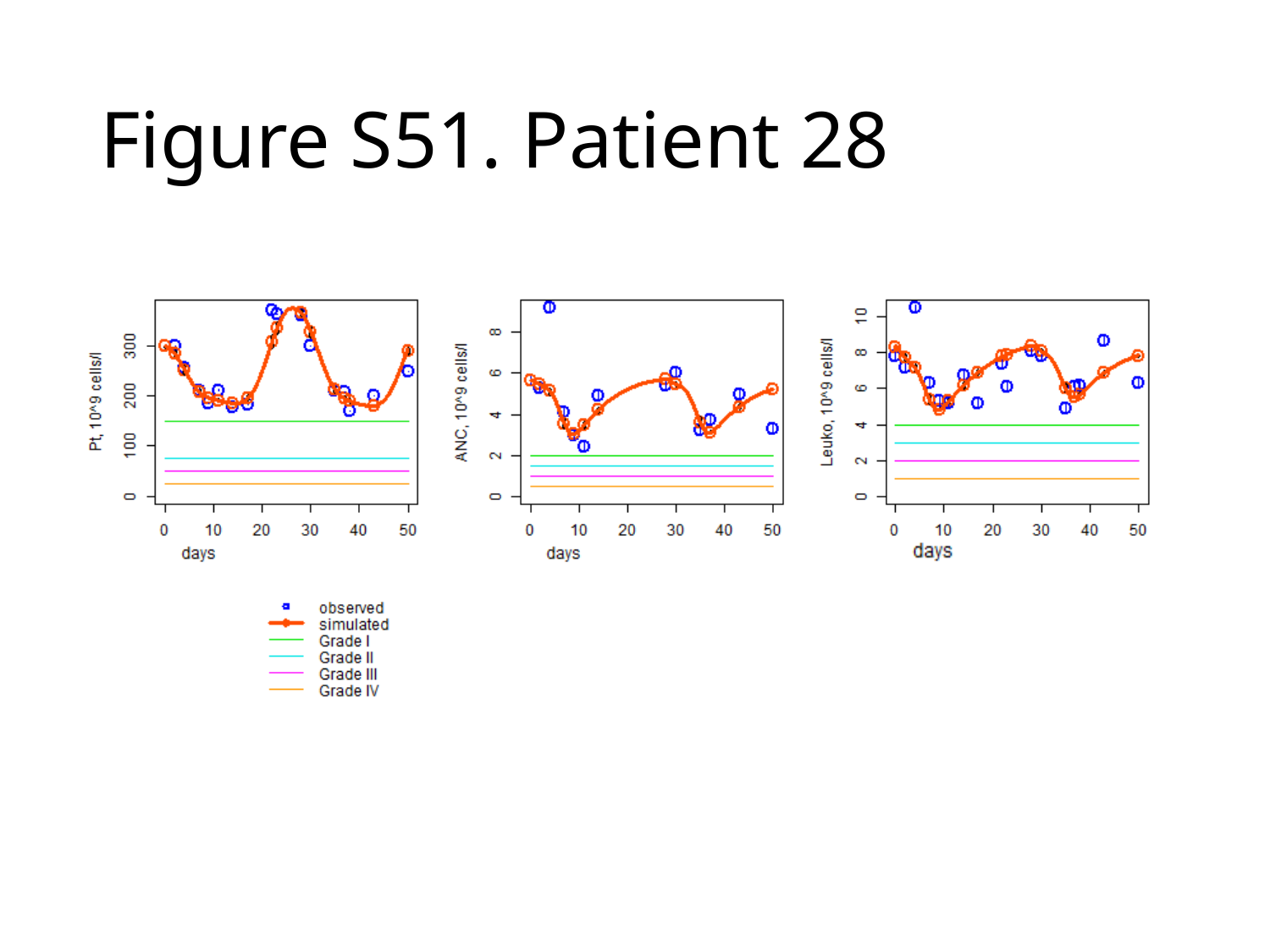

# Figure S51. Patient 28
