# Supplementary material for: Individual hematotoxicity prediction of further chemotherapy cycles by dynamic mathematical models in patients with gastrointestinal tumors
Source: J Cancer Res Clin Oncol. 2023 Feb 28;149(10):6989–98. doi: 10.1007/s00432-023-04601-9 (PMC10374676; doi:10.1007/s00432-023-04601-9)
Supplement: Supplementary file 3 — Supplementary file3 (DOCX 68 KB) [file 432_2023_4601_MOESM3_ESM.docx]

Supporting information

# S1. Additional data used for individual parameter fitting

## Biological data

**Biological data**

Here we briefly present the literature data used for our model development. Mostly, only averaged data are available. Description of Harker et al., Hanson & Slichter and Li et al. data is borrowed from our previous work (Kheifetz and Scholz 2019). Descriptions of other studies are borrowed from our recent work (Kheifetz and Scholz 7/1/2022).

Individual fitting results are shown in figures S1-S23 in Supporting Figures S1-S23 Virtual fits. Red lines show fits for all 28 patients of each measured entity in each virtual experiment. Standard deviations of data are shown as vertical blue tabs. If not available, it is not shown, but assumed to be 30% of the observation. Time axis for all figures are in days.

**Harker et al. (Harker et al. 2000)** Single doses of 3 µg/kg of pegylated TPO were injected subcutaneously into 16 healthy subjects. Total TPO concentrations and platelet counts were measured daily between day 0 and day 28. MKC counts and percentages of MKC ploidies 2-128 were determined at days 0, 7, 11 and 17 after TPO injection. MKC counts of ploidy 16 and 32 showed delayed decrease implying possible mobilization of dormant cells. The fits have good agreement with the data and are shown in Figure S1.

**Hanson and Slichter 1985 (Hanson and Slichter 1985)** Autologous ^51^Cr-labeled platelets were transfused to 16 normal subjects and 27 patients with stable, untreated thrombocytopenia secondary to bone marrow failure. Platelet basic counts range between 12,000 and 70,000/µL. Dynamics of labeled platelets were determined during 5 days after injection. Compared to normal subjects, platelet life span was slightly reduced in patients with platelet counts in between 50,000 to 100,000/µL but was markedly reduced for patients with platelet counts below 50,000/µL. This study demonstrates strong dependence of circulating platelets life span on the basic platelets count implying possibility of constant platelets uptake (Hersh et al. 1998). The fits demonstrate significantly shorter platelets life span, implying possible influence of the gastric cancer on the platelets turn over (Figures S2-S5.)

**Li et al. (Li et al. 2013)** 21 healthy donors undergoing mobilization for allogeneic transplantation before (steady state) or 4–6 h after administration (mobilized) of recombinant human G-CSF (5 mg/kg/day, twice a day, Filgrastim, Japan) for 3–5 days. Their bone marrow niches have been examined before and after treatment. The number of osteoblasts per bone surface was markedly reduced after G-CSF application, implying destruction of osteoblasts and possibly stem/precursor cells niches. The fits have very good agreement as shown in Figure S6.

**Lord et al. (Lord et al. 1989)** Two patients with metastatic breast cancer were treated with rhG-CSF (Amgen) continuously infused for 3 and 5 days, respectively. On the seventh day after starting rhG-CSF therapy, they received doxorubicin (125 mg/m^2^) followed 24 hr later by further rhG-CSF for 11 days to reduce the period of neutropenia. Relative counts of different bone marrow cells as well as their proliferative fraction have been measured just before, 48 and 73 hours after labeling. Dynamics of proliferating granulocytic blasts (PGB) is retrieved from these data by summing up dynamics of myeloblasts, promyelocytes, myelocytes. Dynamics of neutrophils have been monitored as well. Since the patients received different duration of G-CSF transfusion, we considered them as two different population data sources. We used dynamics of PGB, proliferating fraction of PGB and neutrophils dynamics before start of chemotherapy treatment in order to assess the impact of G-CSF on PGB development and mature neutrophils dynamics.

Fits (figures S7, S8) showed good agreements for PGB cells (myeloblasts, promyelocytes and myelocytes) but larger deviations for neutrophils possibly because of fitting individual rather than population virtual data, whose dynamics can differ significantly from that of similar experiment of Borleffs et al. (Borleffs et al. 1998).

**Price et al. (Price et al. 1996)** 13 young healthy patients were treated with a single dose of filgrastim. Relative counts of bone marrow cells (blasts cells, promyelocytes, myelocytes, metamyelocytes, band and segmented neutrophils) have been measured before and 5 days after the treatment. Figure S9 shows good agreement of fits with the observations.

**Morstyn et al. (Morstyn et al. 1989)** Three patients with metastatic malignancies received G-CSF at a dose of 10µg/kg/d by continuous subcutaneous infusion during the first five days, nine days before melphalan treatment. Circulating cell counts of neutrophils, monocytes, lymphocytes, myelocytes and reticulocytes were monitored daily during d0-d10. G-CSF strongly stimulated neutrophils and monocytes without any considerable effect on lymphocytes. We considered averaged values of neutrophils, monocytes and lymphocytes of these three patients. Figure S10 shows good agreements of fits with the observations.

**Johnston et al. (Johnston et al. 2000)** To explore the use of SD/01 (a polyethylene glycol-conjugated filgrastim with prolonged half-life) thirteen patients with non-small-cell lung cancer were randomized to daily receive five doses of filgrastim (5 µg/kg/d) or a single injection of SD/01 (30, 100, or 300 µg/kg) 2 weeks before chemotherapy (cycle 0), and again, 24 hours after administration of carboplatin and paclitaxel (cycle 1). Neutrophils, platelets and G-CSF concentrations have been measured daily during cycle 0 and 1. Here we considered only data from cycle 0 for platelets in order to calibrate inhibiting effects of G-CSF on thrombopoiesis in the branching between thrombopoiesis and myelopoiesis. Figures S11-S14 show fitting results for the respective doses of filgrastim and SD/01. Data has unexplainable high variability in the delay of the effect.

**Roskos et al. (Roskos et al. 2006)** Healthy subjects (8 subjects/dose group) received a single subcutaneous dose of 30, 60, 100, or 300 µg/kg pegfilgrastim.  Pegfilgrastim serum concentration data, banded and segmented cell counts were sampled at least one time daily during 15 days for each dose group. The fits are good and shown in figures S15-S18, correspondingly to the given doses.

**Magee et al. (Magee et al. 2001)** Prednisolone pharmacokinetics (PK) and pharmacodynamics (PD) were investigated in relation to sex and race in white males, black males, white females, and black females (n = 8/group) after a single oral dose (0.27 mg/kg) of prednisone. Prednisone concentration, counts of lymphocytes and neutrophils were sample within 32h. Prednisone increased neutrophil counts and decreased lymphocytes counts. The fits (figure S.19) are good for neutrophils but have a larger than observed delay for lymphocytes.

**Borleffs et al. (Borleffs et al. 1998)** Healthy subjects received subcutaneous injections of filgrastim 75 µg (n = 8), 150 µg (n = 4), 300 µg (n = 4), 600 µg (n = 8), or placebo (n = 6) daily for 10 consecutive days. Blood samples of G-CSF concentration as well as of neutrophil counts were drawn immediately before the first injection and during d1-d10. The fits showed good agreement with the data as depicted in figures S19-S23 according to the dosing group of filgrastim.

# S2. Estimated model parameters

Table S.1. We present population distributions of fitted individual parameters

| Names | units | Population  mean | Population SD | Description |
| --- | --- | --- | --- | --- |
| $b_{Sw,TPO}$ | - | 0.735 | 0.299 | Sensitivity parameter for TPO influence on the lineage commitment of granulopoiesis and thrombopoiesis |
| $b_{Sw,GCSF}$ | - | 0.879 | 0.450 | Sensitivity parameter for G-CSF influence on the lineage commitment |
| *R_Leu,0,nor_* | - | 1.10 | 0.133 | Ratio of initial and steady-state leucocyte count |
| *R_Neu,0,nor_* | - | 1.04 | 0.542 | Ratio of initial and steady-state neutrophil count |
| *c_PD,Osteo_* | - | 0.0249 | 0.0375 | Relative chemotherapy effect on the bone marrow niche |
| *r_PL,0,nor_* | - | 1.07 | 0.0836 | Ratio of initial and steady-state platelet count |
| $b_{A_{CM}}$ | - | 1.50 | 1.20 | Sensitivity parameter of G-CSF action on CM precursors cells |
| $b_{S\_act}$ | - | 0.920 | 0.846 | Sensitivity parameter of G-CSF action on stem cells |
| $b_{rev\_dorm,16}$ | - | 0.266 | 0.189 | Sensitivity parameter of TPO action on transition of inactive (dormant) MKC sub-compartments of ploidy 16 to the active sub-compartment of same ploidy. |
| $b_{rev\_dorm,32}$ | - | 3.36 | 1.35 | Sensitivity parameter of TPO action on transition of inactive (dormant) MKC sub-compartments of ploidy 32 to the active sub-compartment of same ploidy |
| $b_{{MKC}_{p,1}}$ | - | 0.507 | 0.292 | Sensitivity parameter of TPO action on the transition from MKC sub-compartments of ploidies 8, 16, and 32 to the proplatelet compartment |
| *k_m,TPO_* | - | 0.365 | 0.0943 | TPO saturation of specific elimination (Michaelis-Menten constant) |
| *w_PLC_* | cells^-1^ h^-1^ | 1.16 | 0.435 | Maximum TPO elimination rate by circulating platelets |
| *T_PL_* | h | 145 | 21.8 | Transit time of platelets |
| $d_{{Osteo}_{loss}}$ | h^-1^ | 0.0198 | 0.0202 | Elimination rate of dormant precursor (other than megakaryocytes) cells due to lack of osteoblast support |
| $n_{CM}^{unreg}$ | - | 13.1 | 1.74 | Number of divisions of granulopoietic progenitors |
| $b_{{MKC}_{p,64,1}}$ | - | 0.432 | 0.248 | Sensitivity parameter of TPO action on transition from MKC sub-compartment of ploidy 64 to the proplatelet compartment |
| $b_{TG4}$ | - | 0.410 | 0.0673 | Sensitivity parameter of G-CSF action on meta-myelocytes |
| $b_{TG5}$ | - | 0.381 | 0.0479 | Sensitivity parameter of G-CSF action on transition time of banded granulocytes in bone marrow |
| $b_{TG6}$ | - | 0.493 | 0.151 | Sensitivity parameter of G-CSF action on transition time of segmented granulocytes in bone marrow |
| ${rPD}_{MGB4}$ | - | 34.1 | 56.0 | Relative chemotherapy effect on the compartment of meta-myelocytes, compared to those of banded and segmented granulocytes in bone marrow |
| ${rPD}_{Myel}$ | - | 79.2 | 101.6 | Relative chemotherapy effect on myeloblasts |
| ${rPD}_{MGB}$ | - | 0.161 | 0.202 | Relative chemotherapy effect on banded and segmented granulocytes in bone marrow |
| ${rPD}_{Lymph}$ | - | 0.115 | 0.0856 | Relative chemotherapy PD effect on lymphocyte progenitors |
| $D_{\Psi,1}$ | h^-1^ | 0.0249 | 0.0290 | Reverse of waning time of the cytotoxic effects |
| $V_{SD}$ | l | 28.8 | 19.3 | Volume of distribution of the G-CSF derivative SD derivative used in (Johnston et al. 2000) |
| ${rPD}_{SD}$ |  | 0.856 | 0.619 | PD effect of the G-CSF derivative SD relative to Filgrastim |
| $k_{Del,L}$ | - | 108 | 74.9 | Slope parameter of delayed PD effect of prednisolone on lymphopoiesis used for biological (virtual) data only |
| ${PD}_{docet}$ | L· (h·mg)^-1^ | 0.357 | 0.218 | Cytotoxic effect of docetaxel |
| *pd_MKC_* | - | 1.36 | 1.51 | Relative cytotoxic effect (compared to CM) for mature megakaryocytes |
| *pd_MKCimm_* | - | 1.02 | 1.28 | Relative cytotoxic effect (compared to CM) for immature megakaryocytes (ploidy up to 4) |
| $d_{{Osteo,MKC}_{loss}}$ | h^-1^ | 0.143 | 0.343 | Elimination rate of dormant megakaryocytes due to lack of osteoblast support |
| $C_{Pred,50}$ | mg· L^-1^ | 49.7 | 0.302 | Michaelis-Menten constant of prednisolone effect on granulopoiesis used for biological (virtual) data only |
| ${PD}_{Pred,lymph}$ | mg· L^-1^ | 6.12 | 7.15 | Michaelis-Menten constant of prednisolone effect on lymphopiesis for the biological (virtual) data |
| $k_{LCpcf}$ | h^-1^ | 1.43 | 1.23 | Transit rates from peripheral to circulating and from circulating to peripheral compartment of fast lymphocytes |
| $b_{n,Myel}$ | - | 0.612 | 0.0430 | Sensitivity of the number of cell divisions of myeloblasts on G-CSF |
| ${PD}_{oxali}$ | L· (h·mg)^-1^ | 1.18E-05 | 1.18E-05 | Cytotoxic effect of Oxaliplatin |
| ${PD}_{5FU}$ | L· (h·mg)^-1^ | 1.78E-03 | 1.40E-03 | Cytotoxic effect of 5-Fluoruracil |
| ${PD}_{irino}$ | L· (h·mg)^-1^ | 0.184 | 0.442 | Cytotoxic effect of Irinothecan |

# Fitting results: clinical data

Table S.2. Patients’ treatment information

Number of cycles considered in the present analyses. We used data prior to surgery if possible, otherwise post-surgery data were used (patients 22 and 81). G-CSF was applied as pegylated or non-pegylated derivative.

| ID | Number of cycles | Schema | Oxaliplatin | Docetaxel | 5-Fluoruracil (5-FU) | Irinotecan | G-CSF |
| --- | --- | --- | --- | --- | --- | --- | --- |
| 1 | 4 | FLOT | + | + | + | - | - |
| 2 | 4 | FLOT | + | + | + | - | + |
| 3 | 4 | Modified FOLFOX 6 | + | - | + | - | + |
| 4 | 4 | FLOT | + | + | + | - | - |
| 5 | 4 | FLOT | + | + | + | - | + |
| 6 | 4 | FLOT | + | + | + | - | + |
| 7 | 4 | FLOT | + | + | + | - | + |
| 8 | 4 | FLOT | + | + | + | - | - |
| 9 | 4 | FLOT (25% 5-FU) | + | + | + | - | - |
| 10 | 2 | FLOT | + | + | + | - | + |
| 11 | 4 | FLOT | + | + | + | - | - |
| 12 | 4 | FLOT | + | + | + | - | - |
| 13 | 3 | FLO | + | - | + | - | - |
| 14 | 2 | FLOT | + | + | + | - | - |
| 15 | 4 | FLOT | + | + | + | - | - |
| 16 | 2 | FLOT | + | + | + | - | + |
| 17 | 4 | FLOT | + | + | + | - | + |
| 18 | 8 | FOLFIRINOX | + | - | + | + | + |
| 19 | 6 | FOLFIRINOX | + | - | + | + | + |
| 20 | 7 | FOLFIRINOX | + | - | + | + | + |
| 21 | 4 | FLOT | + | + | + | - | + |
| 22 | 5 | FOLFIRINOX | + | - | + | + | + |
| 23 | 4 | FLOT | + | + | + | - | + |
| 24 | 3 | FLOT | + | + | + | - | + |
| 25 | 4 | FLOT | + | + | + | - | - |
| 26 | 4 | FLOT | + | + | + | - | - |
| 27 | 2 | FOLFIRINOX | + | - | + | + | - |
| 28 | 4 | FLOT | + | + | + | - | + |

Figures S.24-S.50 in Supporting Figures S24 S25 RealFits.pptx show the resulted individual fits for all patients. Time scale is in days.

Publication bibliography

Borleffs JC, Bosschaert M, Vrehen HM, Schneider MM, van Strijp J, Small MK, Borkett KM (1998): Effect of escalating doses of recombinant human granulocyte colony—stimulating factor (filgrastim) on circulating neutrophils in healthy subjects. In *Clinical Therapeutics* 20 (4), pp. 722–736. DOI: 10.1016/s0149-2918(98)80135-5.

Hanson SR, Slichter SJ (1985): Platelet kinetics in patients with bone marrow hypoplasia: evidence for a fixed platelet requirement. In *Blood* 66 (5), pp. 1105–1109.

Harker LA, Roskos LK, Marzec UM, Carter RA, Cherry JK, Sundell B, Cheung EN, Terry D, Sheridan W (2000): Effects of megakaryocyte growth and development factor on platelet production, platelet life span, and platelet function in healthy human volunteers. In *Blood* 95 (8), pp. 2514–2522.

Hersh JK, Hom EG, Brecher ME (1998): Mathematical modeling of platelet survival with implications for optimal transfusion practice in the chronically platelet transfusion-dependent patient. In *Transfusion* 38 (7), pp. 637–644. DOI: 10.1046/j.1537-2995.1998.38798346631.x.

Johnston E, Crawford J, Blackwell S, Bjurstrom T, Lockbaum P, Roskos L, Yang BB, Gardner S, Miller-Messana MA, Shoemaker D, Garst J, Schwab G (2000): Randomized, dose-escalation study of SD/01 compared with daily filgrastim in patients receiving chemotherapy. In *Journal of clinical oncology : official journal of the American Society of Clinical Oncology* 18 (13), pp. 2522–2528. DOI: 10.1200/JCO.2000.18.13.2522.

Kheifetz Y, Scholz M (7/1/2022): Individualized mechanistic integral model of human hematopoiesis. Archieve.

Kheifetz Y, Scholz M (2019): Modeling individual time courses of thrombopoiesis during multi-cyclic chemotherapy. In *PLoS computational biology* 15 (3), e1006775. DOI: 10.1371/journal.pcbi.1006775.

Li S, Zhai Q, Zou D, Meng H, Xie Z, Li C, Wang Y, Qi J, Cheng T, Qiu L (2013): A pivotal role of bone remodeling in granulocyte colony stimulating factor induced hematopoietic stem/progenitor cells mobilization. In *Journal of cellular physiology* 228 (5), pp. 1002–1009. DOI: 10.1002/jcp.24246.

Lord BI, Bronchud MH, Owens S, Chang J, Howell A, Souza L, Dexter TM (1989): The kinetics of human granulopoiesis following treatment with granulocyte colony-stimulating factor in vivo. In *Proceedings of the National Academy of Sciences of the United States of America* 86 (23), pp. 9499–9503. DOI: 10.1073/pnas.86.23.9499.

Magee MH, Blum RA, Lates CD, Jusko WJ (2001): Prednisolone pharmacokinetics and pharmacodynamics in relation to sex and race. In *Journal of clinical pharmacology* 41 (11), pp. 1180–1194. DOI: 10.1177/00912700122012733.

Morstyn G, Campbell L, Lieschke G, Layton JE, Maher D, O'Connor M, Green M, Sheridan W, Vincent M, Alton K et al. (1989): Treatment of chemotherapy-induced neutropenia by subcutaneously administered granulocyte colony-stimulating factor with optimization of dose and duration of therapy. In *Journal of clinical oncology : official journal of the American Society of Clinical Oncology* 7 (10), pp. 1554–1562. DOI: 10.1200/JCO.1989.7.10.1554.

Price TH, Chatta GS, Dale DC (1996): Effect of recombinant granulocyte colony-stimulating factor on neutrophil kinetics in normal young and elderly humans. In *Blood* 88 (1), pp. 335–340.

Roskos LK, Lum P, Lockbaum P, Schwab G, Yang BB (2006): Pharmacokinetic/pharmacodynamic modeling of pegfilgrastim in healthy subjects. In *Journal of clinical pharmacology* 46 (7), pp. 747–757. DOI: 10.1177/0091270006288731.
